# Generic Protocols for the Analytical Validation of Next-Generation Sequencing-Based ctDNA Assays: A Joint Consensus Recommendation of the BloodPAC’s Analytical Variables Working Group

**DOI:** 10.1093/clinchem/hvaa164

**Published:** 2020-09-01

**Authors:** James H Godsey, Angela Silvestro, J Carl Barrett, Kelli Bramlett, Darya Chudova, Ina Deras, Jennifer Dickey, James Hicks, Donald J Johann, Rebecca Leary, Jerry S H Lee, Joe McMullen, Lisa McShane, Katherine Nakamura, Aaron O Richardson, Matthew Ryder, John Simmons, Kelli Tanzella, Laura Yee, Lauren C Leiman

**Affiliations:** h1 Illumina, San Diego, CA; h2 Novartis Pharmaceuticals, Cambridge, MA; h3 AstraZeneca, Waltham, MA; h4 Thermo Fisher Scientific, Austin, TX; h5 Guardant Health, Redwood City, CA; h6 Personal Genome Diagnostics, Baltimore, MD; h7 University of Southern California, Los Angeles, CA; h8 University of Arkansas, Little Rock, AR; h9 National Cancer Institute at the National Institutes of Health (NIH/NCI), Rockville, MD; h10 Thermo Fisher Scientific, Carlsbad, CA; h11 Sysmex Inostics, Baltimore, MD; h12 Thermo Fisher Scientific, Grand Island, NY; h13 BloodPAC, Chicago, IL

**Keywords:** ctDNA, cfDNA, NGS, plasma, liquid biopsy, analytical validation

## Abstract

Liquid biopsy, particularly the analysis of circulating tumor DNA (ctDNA), has demonstrated considerable promise for numerous clinical intended uses. Successful validation and commercialization of novel ctDNA tests have the potential to improve the outcomes of patients with cancer. The goal of the Blood Profiling Atlas Consortium (BloodPAC) is to accelerate the development and validation of liquid biopsy assays that will be introduced into the clinic. To accomplish this goal, the BloodPAC conducts research in the following areas: Data Collection and Analysis within the BloodPAC Data Commons; Preanalytical Variables; Analytical Variables; Patient Context Variables; and Reimbursement. In this document, the BloodPAC’s Analytical Variables Working Group (AV WG) attempts to define a set of generic analytical validation protocols tailored for ctDNA-based Next-Generation Sequencing (NGS) assays. Analytical validation of ctDNA assays poses several unique challenges that primarily arise from the fact that very few tumor-derived DNA molecules may be present in circulation relative to the amount of nontumor-derived cell-free DNA (cfDNA). These challenges include the exquisite level of sensitivity and specificity needed to detect ctDNA, the potential for false negatives in detecting these rare molecules, and the increased reliance on contrived samples to attain sufficient ctDNA for analytical validation. By addressing these unique challenges, the BloodPAC hopes to expedite sponsors’ presubmission discussions with the Food and Drug Administration (FDA) with the protocols presented herein. By sharing best practices with the broader community, this work may also save the time and capacity of FDA reviewers through increased efficiency.

## Introduction

Circulating tumor DNA (ctDNA) is genomic material shed by apoptotic and necrotic tumors into peripheral circulation *(*1*)*. It typically represents only a small portion of cell-free DNA (cfDNA) present in the blood, which can originate from many different sources such as infectious organisms, fetal DNA during pregnancy, and genomic DNA from white blood cells *(*2*)*. The presence of tumor-associated mutations allows highly specific discrimination of ctDNA from normal DNA. However, the relatively low abundance of ctDNA compared to cfDNA means that there are few mutant molecules present, necessitating well-characterized and analytically robust ctDNA assays to ensure the reliability of molecular information.

The use of ctDNA to obtain tumor genomic information via a minimally invasive blood draw has attracted a significant amount of attention in the era of precision medicine. In addition to the ease of accessibility and reduced risk to the patient compared to tissue biopsy, several other characteristics of ctDNA make it an attractive tool for use in the clinic. First, ctDNA analysis can be performed very quickly compared to tissue analysis that has a greater number of preanalytic steps that result in increased turnaround time and can significantly delay the administration of appropriate therapy *(*3, 4*)*. As invasive tissue biopsy carries with it risks inherent for any surgical procedure *(*5, 6*)*, molecular analysis of tissue is usually limited to a single sample collected at a single time-point, which may not be sufficient to capture the dynamic nature of a tumor that is constantly evolving under selective pressure from different clinical interventions. Moreover, the heterogeneous nature of the complete population of tumor cells present in a patient means that molecular analysis across different sections of a tissue specimen may be subject to biological variation. Biopsy of a single anatomic location may also fail to capture a global picture of disease, thus missing the genomic profiles of cells present at other sites such as distant metastases. In contrast, ctDNA is easily obtainable through collection of multiple specimens corresponding to clinically important time points, such as at baseline diagnosis, after surgical resection of a tumor, at progression, or other clinically relevant time points and across the course of different therapeutic regimens. It is also hypothesized that ctDNA may better capture disease heterogeneity since tumor-derived mutant molecules that originate from all disease sites across the body can be sampled via peripheral blood draw *(*7*)*.

Despite these advantages, evidence generation for use of ctDNA in clinical practice has progressed at a measured pace and there are currently only a few applications where ctDNA results are routinely used to inform a clinical decision. Specifically, it has been demonstrated that the use of liquid biopsy in treatment selection for previously untreated patients with nonsmall cell lung carcinoma leads to discovery of guideline-recommended biomarkers at a rate that is similar to that of standard-of-care tissue genotyping, with high tissue concordance and more complete genotyping *(*8*)*. Moreover, patients identified for targeted treatment using liquid biopsy have demonstrated similar therapeutic response rates to patients identified with tissue testing *(*5*)*.

Clinical practice guidelines now recognize the utility of ctDNA analysis in advance of intrathoracic biopsy and tissue analysis for patients with nonsmall cell lung cancer who are potentially eligible for an *EGFR* tyrosine kinase inhibitor *(*9–12*)*. Additionally, a real-time PCR-based ctDNA test was recently approved contemporaneously with the first approved therapy targeting *PIK3CA* for patients with estrogen receptor-positive breast cancer who progress on adjuvant hormone therapy and are in need of a second-line option *(*13*)*. However, in both cases, guidelines recommend that if a negative ctDNA result is obtained, patients should be reflexed to tissue testing. This is particularly relevant in the context of hotspot testing using quantitative PCR, which has limited sensitivity compared to technologies such as digital PCR and NGS, since a negative result does not differentiate absence of the mutation of interest from lack of sufficient ctDNA in the sample. In contrast, with NGS-based tests, tumor profiling information obtained panel-wide can often differentiate these 2 scenarios by identifying tumor-derived alterations at higher allele frequencies and suggesting true absence of mutation in a background of detectable ctDNA levels. Therefore, it follows that to minimize the number of patients who undergo invasive tissue biopsy (or potentially receive no conclusive molecular testing results at all); ctDNA assays must be well designed and capable of demonstrating robust performance for their clinical intended uses.

In addition to the selection of targeted therapies, ctDNA presents an attractive option that may have potential use as a minimally invasive biomarker to monitor disease status. For example, Kruger et al. *(*14*)* recently demonstrated that serial monitoring of ctDNA as identified by the presence of a *KRAS* gene mutation for patients with advanced pancreatic cancer receiving chemotherapy might be useful for early response prediction and therapeutic monitoring. The persistence of ctDNA postsurgical resection of a primary tumor may also be indicative of microscopic occult disease, which may drive disease recurrence *(*9, 15*)*. Liquid biopsy also has been successfully used to predict therapeutic response based on early sampling of ctDNA levels 4–6 weeks posttherapeutic intervention in lung and bladder cancer *(*9*)*. However, these and other potential clinical uses of ctDNA testing are still under study and will likely require diagnostic tools, which have demonstrated unique performance characteristics. For instance, a ctDNA test used for therapeutic response monitoring will need to demonstrate reliable quantitative detection of the absolute number of mutant ctDNA molecules per unit of volume of plasma to provide the highest resolution information on dynamic changes in disease status as compared to a qualitative “mutation detected” versus “no mutation detected” readout sufficient for a targeted therapy selection test.

As ctDNA tests, particularly those based on NGS, continue to advance toward adoption into routine clinical practice, there will be a need for a standardized approach to assay validation to ensure the legitimacy of information on which clinical decisions increasingly will be based. Historically, the role of standards, guidelines, and best practices are prominent in US healthcare and in technology industries globally, and can function as vital and stabilizing entities, especially during times of disruptive change. For instance, the National Comprehensive Cancer Network^®^ (NCCN^®^) guidelines have helped to establish national standards-of-care, clinical best practices, along with rank-ordered category recommendations for a broad range of cancers. In the field of medical testing, the Clinical and Laboratory Standards Institute (CLSI) has developed a range of global standards to harmonize the clinical laboratory practices and the development of diagnostic devices *(*16*)*. While these guidelines are informative to the practice of ctDNA testing and validation, they do not entirely address the level of stringency or the workflow challenges presented in the development of highly sensitive, ctDNA-based NGS tests, as recognized by BloodPAC in the drafting of these generic protocols.

With the release of the FDA’s finalized guidance around the Breakthrough Devices Program, the BloodPAC is encouraged to see several members receiving breakthrough designation of their NGS-based ctDNA tests. However, the BloodPAC recognizes that despite this move toward innovation in the field, few ctDNA-based tests have received FDA approval to date *(*17*)*. In addition, there is currently uncertainty in the oncology community about the reliability of ctDNA testing, which has been fueled by studies showing discordance with tissue genotyping *(*18*)* as well as studies showing discordance among plasma-based tests, especially for detection of low-frequency mutations *(*19, 20*)*. These studies highlight the necessary caution as these technologies advance toward FDA approval and more widespread clinical practice. Guidelines and best practices are crucial for the successful development of robust and accurate liquid biopsy clinical assays.

The BloodPAC Consortium also recognizes that, although analytical validation studies for tissue-based tests are, in many ways, similar to those that should be performed for ctDNA assays, several unique biological considerations must be taken into account. These considerations include the low concentration of analyte (down to several mutant molecules per 10-mL blood collection tube (BCT)), which both challenges the detection capabilities of assays as well as necessitates an increased use of contrived specimens for validation, for which functional equivalence with native ctDNA must be demonstrated. Another consideration is that normal cfDNA will also be present in abundance relative to the concentration of ctDNA and may fluctuate for a variety of biological and clinical reasons unrelated to disease status. In addition, the presence of nontumor-derived mutations in the blood as an endogenous analyte, i.e., germline or clonal hematopoiesis of indeterminate potential (CHIP) pose another challenge. Novel technology solutions have been developed for ctDNA analysis, such as the use of unique molecular identifiers to increase the sensitivity and specificity of NGS ctDNA assays *(*18*)*. Preanalytical procedures must also be optimized to ensure not only the stability of the ctDNA analyte, but also to prevent the release of large quantities of wild type (WT) genomic DNA from white blood cells that could dilute the detectable signal. Finally, analysis methodologies must be optimized to enable accurate detection of signal in the background of PCR and sequencing errors.

Under this context, the Analytical Variables Working Group (AV WG), within the larger BloodPAC Consortium, approached the FDA to propose collaboration on a set of generic analytical validation protocols designed to ensure the robustness and standardization of assay validation while specifically addressing the unique challenges of ctDNA testing. Utilizing the collective participation among all BloodPAC participants including members from biotech and pharmaceutical industries, academia, and US Government scientific and regulatory sectors, the AV WG developed a series of generic analytical validation protocols ((21), see the online Data Supplement for Material 1). These generic protocols are intended to help guide the analytical validation of NGS-based ctDNA tests that yield information that can help inform treatment decision-making. Our goal is to create a standardized generic starting point for all analytical validation protocol discussions such that methods described can be adapted and applied for any NGS ctDNA assay, irrespective of test design, enrichment technology, or any other workflow component. Through this guidance, the BloodPAC seeks to accelerate and streamline the submission process for both test sponsors as well as the FDA and, most importantly, to safeguard the quality of molecular information that will be used to enable therapeutic decision-making for patients. In addition, studies described are primarily specific for treatment decision-making in patients who have already been diagnosed with late-stage solid tumors. Due to the rapid advancement of ctDNA technologies and clinical applications and the corresponding evolution of the regulatory landscape, the AV WG views this exercise as a balance between conveying current information and anticipating future practices. To date, we have created 11 generic analytical validation protocols along with 4 standard methods, which are described next.

## Materials and Methods

During Q4 of 2017, the BloodPAC AV WG was formed, with leadership by James Godsey and Angela Silvestro to ensure balanced inputs from diagnostic and therapeutic sectors. The kickoff meeting for this new working group took place at the BloodPAC quarterly meeting held in December 2017. The initial step to forming this working group was to gather a group of key BloodPAC members who represented in vitro diagnostic manufacturers, and pharmaceutical, biotechnology, academic, and government organizations. This working group included 20 members and 2 cochairs and represented 11 companies and organizations.

Once the membership was established, the AV WG aligned on a common objective to provide a complete set of generic analytical validation protocols designed to provide test developers/manufacturers with a core baseline of standardized analytical validation protocols with which to document a ctDNA assay’s analytical performance.

Given this objective, the AV WG also aligned on a set of key assumptions to focus the scope of the effort:

1. The protocols will be used for assays with a generic intended use. Namely, these protocols are intended for the validation of NGS-based ctDNA assays that yield information to aid treatment decision-making in patients who have already been diagnosed with advanced solid tumors. These protocols are not intended for the validation of screening assays or assays for early detection.
2. The protocols are designed for the validation of assays with a locked assay design that has been developed under design control.
3. These protocols aim to produce validation data intended to be submitted for regulatory review.
4. These protocols are designed to be technology and design independent (i.e., are not specific to one chemistry, instrument, or bioinformatics pipeline).

Following alignment on objectives and assumptions, the members met to define study designs based on best practices in the field, existing assay validation guidance documents (such as from CLSI, New York State Department of Health, and the Association for Molecular Pathology), and the unique performance challenges for NGS-based ctDNA assays. The AV WG decided to follow a standard format, proposed by the FDA’s Center for Devices and Radiological Health (CDRH), in which each protocol contains an introduction, experimental design, a statistical analysis section, and an example data presentation format. In addition, a set of standard methods around sample collection and preparation were also included in the document. Biostatisticians were consulted to define the minimal test requirements, sample size, and to give guidance on the appropriate statistical analysis.

Throughout the drafting process, the AV WG regularly consulted with the FDA’s CDRH to align on the approach and direction of these generic protocols. Revision 1 of the protocol document was submitted to CDRH in the form of a presubmission in August 2018. Based on the FDA’s feedback, the AV WG made additional revisions and submitted Revisions 2 and 3 of the document as supplemental presubmissions to CDRH in February and July of 2019, respectively. After iterative discussions, the FDA’s inputs were incorporated into the following set of protocols (see Supplemental Material 1).

## Results

### Overview of Generic Analytical Validation Protocols and Standard Methods

A summary outline of the experimental designs for the analytical validation studies is found in Table 1 and includes general guidance on minimal sample size. A set of standard methods is also summarized in Table 2. Detailed protocols and methods, including statistical analyses methods are found in Supplemental Material 1. It is expected that assay developers will determine an appropriate sample size for demonstration of performance to support claims for each unique assay. For some studies (e.g., accuracy), the minimal sample size must be determined by the lower bound of the acceptance criteria. For other studies that establish analytical performance based on point estimates or the performance goal of the assay [e.g., limit of blank (LoB) or limit of detection (LoD)], the minimal sample size should sufficiently address the uncertainty of the performance estimates to support the claims. Unless specified, the number of lots, sites, and/or equipment, and operators per study are to be determined by the assay developer. In addition to these performance factors, new assay developers should also note that BCTs are an important component of any ctDNA assay and therefore should be selected and evaluated appropriately for each assay’s intended use. The selection of an appropriate BCT should be made prior to the start of analytical validation.

**Table 1:** Summary of analytical validation protocols.

Protocol name	Sample types	Experimental design	Statistical analysis	Data presentation format
Reference Interval (quantitative claims only)	Age- and risk-matched normal donors.	Test 120 reference donors, using the CHIP subtraction/filtering method that will be used in the final assay design.	Estimate the lower and upper reference limits as the 2.5th and 97.5th percentiles of the distribution of test results for the reference population, respectively, for each variant in the panel with a quantitative claim.	Present the reference interval for each variant with a quantitative claim.
Limit of Blank (LoB)	Age- and risk-matched normal donors and mutation-negative patient samples.	Test at least 60 total blank samples with each of 2 reagent lots, at an input that is at the high end of the assay’s input requirements. Use the CHIP subtraction/filtering method that will be used in the final assay design.	**Variants with qualitative claims:** use the nonparametric method, per CLSI EP17. **Variants with quantitative claims:** depending upon the distributions of the blank sample results, a nonparametric, or more rarely, a parametric data analysis option is selected (CLSI EP17).	LoB estimates may be set as zero, and then blank samples may be tested to confirm the LoB. Developers should report the false positive results at the LoB cutoff observed on a per-variant basis, both for hotspots and panel-wide variants, and on a per-sample basis.
Contrived Sample Functional Characterization Study (CSFCS)	At least 1 paired set of 1 clinical sample and 1 contrived sample.	The top-level dilution of each sample should be paired in level and quantified using an orthogonal method. At least 5 dilutions of each starting sample should be prepared and at least 1 dilution tested between LoB and LoD. Test 20 replicates per level. CSFC study may be combined with the LoD or linearity study, as appropriate.	**Variants with qualitative claims:** for each mutation type, plot the value determined by the orthogonal method at the top level and the dilution scheme at lower levels (*x*) vs. % positive calls by the assay (*y*) for the clinical and contrived samples. Fit a logit or probit regression model as described in the Supplemental Material 1. **Variants with quantitative claims:** use the method comparison approach to estimate the agreement between the 2 sample types (contrived and clinical) for that variant, as described in Supplemental Material 1.	**Variants with qualitative claims:** tabulate results to include the coefficients, LoD, C75, C50, C25, and C5, as point estimates and 95% CIs for clinical samples, contrived samples, and the difference between the 2. **Variants with quantitative claims:** plot the regression model in which *x* axis denotes observed values from clinical samples and *y* axis denotes observed values for contrived samples. A Bland–Altman plot may also be used to plot of the difference (*x*–*y*) on the vertical axis versus the average (*x*+*y*)/2 on the horizontal axis.
Limit of Detection (LoD)	Contrived samples or pooled clinical samples for confirmation.	Create a panel of at least 5 dilutions around the targeted LoD of an appropriate number of low-level positive samples or specimen blends, as needed to represent all variant classes to be detected in the assay. Test at least 10 replicates of each sample using 2 reagent lots.	**Variants with qualitative claims:** use the probit regression model. **Variants with quantitative claims:** parametric analysis may be used to calculate the LoD estimates. Nonparametric method and probability models could also be used (CLSI EP17 and see Supplemental Material 1). **Confirmation of LoD:** determine the hit rate for each variant with clinical samples if contrived samples were used to establish LoD.	LoD should be reported in the same unit(s) as the clinical cutoff (e.g., MAF^a^ for SNVs^b^ and indels^c^; copy number for CNVs^d^, fusion reads for rearrangements, and fusions, etc.). Present the Probit regression model and hit rates for each dilution for each relevant variant type.
Analytical Accuracy	Clinical samples.	**Variant negative samples:** Sequence a minimum of 100 samples known to be negative for cancer-relevant mutations across all regions interrogated by the assay. **Variant positive samples:** Test 10 to 20 positive samples for each type of variant included in the assay’s Level 1 claims with both the assay and an orthogonal method.	**Variants with qualitative claims:** concordance (agreement) of the assay with the orthogonal method is used to calculate accuracy. See the Supplemental Material 1 for guidance on calculating PPA^e^, NPA^f^, and OPA^g^. **Variants with quantitative claims:** accuracy should be assessed by comparing the quantitative analyte (e.g., MAF) measured by both the new ctDNA assay under development and by an appropriate orthogonal (reference) method. The method comparison approach may be used.	**Variants with qualitative claims:** present data as concordance tables that include CIs^h^. Results must include no calls (i.e., samples that failed), and results excluding no calls may be presented. **Variants with quantitative claims:** Present plots as described before and tabulate point estimates of coefficients and regression lines at medical decision points, along with their associated two-sided 95% CIs.
Linearity (Quantitative claims only)	Contrived samples or pooled clinical samples.	If using contrived samples, they should have demonstrated equivalence to clinical samples in the CSFC study. Test at least 11 levels that cover the entire anticipated measuring range of the assay. It is recommended to test over a range that is 20 to 30% wider than the anticipated measuring range. Test at least 4 replicates at each level.	A polynomial evaluation should be completed to determine if the fit is linear. If a nonlinear polynomial fits the data better than a linear one, then the difference between the best-fitting nonlinear and linear polynomial should be assessed against the allowable (predefined) bias for the method.	Present the data in a graphical plot of observed allele frequency on the *y* axis, displaying individual replicate results, versus expected allele frequency on the *x* axis. The regression line should include pointwise 95% CIs.
Limit of Quantitation (LoQ) (Quantitative claims only)	Contrived samples or pooled clinical samples.	Panels should be comprised of at least 4 independent pools of contrived samples that have demonstrated equivalence to clinical samples in the CSFC study. Select a target concentration (expected LoQ) and prepare 4 replicates at this level, as needed to represent all variant classes to be detected by the assay, by diluting into extracted wild type (WT) ctDNA. Test at least 36 replicates with each of 2 manufacturer’s lots over at least 3 days (runs).	For each reagent lot, select the sample with the lowest concentration that met the accuracy specifications as the LoQ for the lot. The greatest LoQ across all lots or the LoQ from the combined dataset is taken as the LoQ for the measurement procedure. A variant approach may also be appropriate that enables evaluation of the LoQ as part of a LoD evaluation using the precision profile approach.	State the determined LoQ.
Reproduci bility/ Repeatability	Clinical samples preferred, may use contrived samples, if appropriate.	**Reproducibility:** test 3 manufacturer’s lots and at least 2 replicates per sample per run, tested over the course of 20 days (runs) per CLSI EP05. For 1 instrument at each of 3 sites, 2 operators per site will perform testing. For a single site using 3 instruments, 2 operators at the site will perform testing on the 3 instruments. Repeatability/**Intermediate Precision:** at 1 site with at least 3 instruments, 1 operator will test at least 10 replicates per sample on each instrument, over the course of 20 days. The repeatability study may be combined with the reproducibility study described before.	**Variants with qualitative claims:** perform agreement analysis based on the assay’s binary output (variant detected vs. not detected). Calculate the two-sided 95% CIs for the agreements. See Supplemental Material 1 for further details. **Variants with quantitative claims:** use an analysis of variance (ANOVA) or a similar model for random effects or components of variance model (typically with multiple factors) to identify the sources of variation.	Tabulate the overall mean and standard deviation observed across conditions.
Interfering Substances	Contrived samples or pooled clinical samples plus normal donor.	Include at least 1 variant positive specimen (at 1× to 1.5× LoD) and 1 WT sample (derived from normal donors); contrived samples may be used. The minimal sample size should be determined for each assay based on its performance goal. Test all specimens with and without (control group) each interferent present, at “worst case” levels. Interfering substances may be tested individually or pooled to reduce the total number of samples to be tested.	Visually inspect the plotted data and assess whether there is a systematic bias (difference) between the selected specimens with interferent and the control group. Determine average positive agreement (APA) and average negative agreement (ANA) statistics at the variant level, with and without no calls (potential assay failures). APA and ANA are weighted averages of PPA and NPA.	Tabulate the descriptive statistics (by variant and condition, the number of observations, mean, and standard deviation) and difference of the condition from control, expressed as a percentage. Tabulate the APA and ANA results.
Guard banding	Contrived samples or pooled clinical samples plus normal donor.	Identify the most critical steps in their specimen collection and/or NGS test process to alter. Test at least 1 variant positive specimen (at 1× to 1.5× LoD) and 1 WT sample (derived from normal donors) under the standard and altered test conditions.	Perform an ANOVA to compare the assay performance run under the standard conditions and among the altered conditions tested to assess robustness. Also determine the mean and standard deviation (SD) of each condition.	Tabulate results and include for each guard banding condition and level tested the number of replicates tested, mean value, SD, and upper and lower CIs. Graphically, plot the replicates as box plots as response versus guard banding conditions.
Prepared Specimen Stability	Contrived samples or pooled clinical samples plus normal donor.	Test at least 3 prepared variant positive specimens (clinical samples at 1–1.5× LoD) and 1 WT specimen (derived from normal donors). Test from baseline (0 months) in 3-month increments up to the desired stability period (typically a minimum of 6 months and up to 24 months).	A linear least squares regression analysis may be used to evaluate the stability of prepared specimens.	Present the data in a graphical plot of observed MAF versus time, displaying individual replicate results. The regression line should include pointwise 95% CIs, and the acceptance criteria curves should also be displayed. Provide the regression parameters (intercept, slope, slope *P* value).

a Mutant allelic fraction.

b Single nucleotide variant.

c Insertion/deletion.

d Copy number variation.

e Positive percentage agreement.

f Negative percentage agreement.

g Overall percentage agreement.

h Confidence interval.

**Table 2:** Summary of standard methods.

Standard method	Overview
Collection of Normal Human Plasma	Minimize pooling of plasma from multiple donors; single donors are preferred. Collect blood in the appropriate blood collection tube (BCT) for the assay’s intended use. Process the plasma within the recommended stability window for that BCT. See also the Supplemental Material 1.
Preparation of Patient Sample Pools	Quantify the variant of interest in a patient’s total ctDNA at the DNA molecule level. Calculate the appropriate volume of each patient ctDNA and wild type (WT) ctDNA to add to pool in order to achieve the targeted MAF.^a^ Confirm that the targeted MAFs have been reached through an orthogonal quantification step.
Preparation of Contrived Samples using ctDNA from Cell Culture Media	Extract mutant-positive ctDNA from cell culture of the appropriate cell line. Culture the cell lines and collect cell culture media as specified in Bronkhorst et al. 2016 *(22)*. Quantify ctDNA using an orthogonal method and dilute into WT ctDNA derived from plasma from normal donors. Confirm that the targeted MAFs have been reached through an orthogonal quantification step. See also the Supplemental Material 1.
Preparation of Contrived Samples using Fragmented Cell Line Genomic DNA	Extract ctDNA from cell line cells and fragment. Quantify fragmented ctDNA and assess fragment size. Dilute into fragmented WT ctDNA extracted from plasma from normal donors. Confirm that the targeted MAFs have been reached through an orthogonal quantification step. See also the Supplemental Material 1.

a Mutant allelic fraction.

The AV WG considers the work that follows as a baseline that ctDNA assay developers/manufacturers can utilize in their initial presubmission discussions with the FDA. As stated in the Methods section, these protocols are designed for the validation of assays with a locked assay design that has been developed under design control.

CHIP, which refers to the clonal expansion of white blood cells derived from a hematopoietic stem or progenitor cell that have acquired one or more somatic mutations *(*23*)*, poses a unique challenge for NGS-based ctDNA assays since it can cause the apparent detection of tumor-associated somatic mutations in the plasma of healthy, aging individuals. In fact, these apparent somatic mutations are known to be the result of mutations affecting white blood cells, which may be considerably expanded in individuals of advanced age. As CHIP results in the true presence of mutant DNA molecules in circulation, it does not cause analytical false positives; however, because these mutations are not tumor-derived, detection of CHIP by a ctDNA test results in a clinical false positive liquid biopsy result *(*24*)*. Therefore, clonal hematopoiesis is a confounding factor for liquid biopsy assays that necessitates differentiation between somatic and CHIP variants. In addition, these CHIP variants detected in plasma may contribute to the discordance observed between tissue and plasma-based assays *(*24*)*.

One recommended approach for exclusion of nontumor-associated CHIP mutations is to sequence matched white blood cells at the same depth as plasma. Developers may also choose to address CHIP bioinformatically; as each laboratory has different methods for delineation of CHIP mutations, CHIP-filtering approaches are generally proprietary and specific to each developer’s platform. One example approach is the use of computational methods to distinguish between germline and somatic variants by modeling the expected allelic frequencies of germline, somatic, and subclonal mutations using the mutant allelic fraction and sample purity *(*25*)*. Developers should consider the benefits and disadvantages of each method to address CHIP carefully and weigh each in light of the assay’s intended use and performance goal. For example, filtering CHIP by sequencing matched normal in parallel with ctDNA may be confounded if circulating tumor cells, which harbor tumor variants, are coisolated with white blood cells *(*26*)*. Another potential difficulty of this approach is that assays optimized for ctDNA analysis may not demonstrate sufficient performance for the identification of CHIP via matched normal sequencing. Alternatively, developers who use algorithmic approaches to address CHIP must demonstrate that the specificity and sensitivity goals of the test’s intended use are met.

Since CHIP may affect the false positive rate of the assay, developers should test samples used in the LoB and Reference Interval studies using the final device’s CHIP-filtering method. All subsequent analytical validation studies should also be completed using the final device’s method for filtering CHIP.

### A Note on Laboratory Developed Tests

The following protocols were drafted with input from the FDA and may be useful for the generation of validation data for the regulatory approval of new in vitro diagnostic assays. Developers of laboratory developed tests (LDTs) that utilize plasma-based NGS assays may also find these best practices informative. All developers should consult with a statistician to determine the sample number and study size appropriate for each assay’s intended use and performance goal. Developers of LDTs may reference the following guidance, as well as those from New York State, the College of American Pathologists, and CLIA *(*27*)*.

### Reference Materials

Reference material samples should be analyzed regularly at defined run intervals to detect possible sources of error and to monitor assay performance *(*28*)*. These materials should be chosen to reflect the size distribution and yield (for full-process materials that require extraction) of ctDNA derived from patient samples and should be functionally comparable to patient samples. Reference materials should be prepared carrying variants at levels that can confirm the performance of the assay for its intended use as demonstrated by the assay’s analytical validation studies. Reference materials may include commercially available materials (see Supplemental Materials 2) or may be prepared as described in Table 2. Blank or “No-Template Control” samples such as water or Tris-EDTA buffer should also be included to identify possible contamination affecting the assay’s laboratory workflow.

## Discussion

In an effort to accelerate the approval and commercialization of ctDNA assays in the liquid biopsy field, the BloodPAC Consortium has developed, via the formal FDA presubmission process, a document defining a set of generic analytical validation protocols and standard methods customized explicitly for NGS-based ctDNA assays. Utilizing its diverse membership, which includes the FDA, the BloodPAC’s AV WG’s generic analytical validation protocols allow ctDNA test developers to initiate presubmission discussions with regard to analytical validation test protocols specific to their test’s intended use, at a much greater level of protocol maturity and completeness. As a result, this minimizes the time spent in presubmission review (each cycle is ∼90 days), with the end result being the acceleration of the approval of new ctDNA assays. The BloodPAC’s generic analytical validation protocols also minimize the time the FDA reviewers need to invest in the multiple rounds of reviews of presubmissions focused on analytical validation for the same reasons. This collaboration results in a win-win situation for both sides; test developers come to the FDA with protocols that are already compliant to the FDA’s guidance and, in turn, the test developer gains months and/or quarters in bringing a new ctDNA to market.

The 11 protocols presented herein fulfill the need for standards and best practices by providing guidance on the execution and analysis of validation studies for NGS-based ctDNA assays, including recommended sample types, suggested sample size, and frameworks for statistical methods and data presentation. In addition, the AV WG has also provided 4 standard methods for the collection and preparation of specimens and for the production of contrived cfDNA samples in support of these protocols. These sets of protocols should be viewed as a starting point of a “living document.” The protocols may need to evolve as our scientific understanding of cancer continues to improve and technology advances.

Though the primary focus of this work has been validation of ctDNA assays, which may be used to guide administration of targeted therapies and for response monitoring, the BloodPAC’s AV WG will also consider assay performance characterization necessary to support novel uses for ctDNA analysis in the future. In particular, as evidence continues to increase in support of the validity of molecular measurable residual disease assessment to inform clinical decisions, the need for ultra-sensitive ctDNA detection is now paramount. Additionally, the BloodPAC Consortium is uniquely positioned to further advance the development and implementation of liquid biopsies by combining development of best practices with our expertise in data collection and management via the the BloodPAC Data Commons, an open repository for liquid biopsy scientific and clinical data *(*21, 29*)*. This will allow us to address additional challenges to the implementation of liquid biopsy in a clinical setting such as discordance between liquid biopsy and tissue biopsy, challenges to ctDNA detection in low tumor burden disease, and difficulties associated with use of liquid biopsy for disease monitoring. An additional area of focus also includes the evaluation of reference materials for use in cfDNA assay development and validation. The BloodPAC will continue to work with organizations in the field such as the American Association for Clinical Chemistry, Association for Molecular Pathology, and CLSI to advance these areas and develop community supported guidelines and frameworks.

## Supplemental Material

Supplemental material is available at *Clinical Chemistry* online.

## Supplementary Material

hvaa164_Supplementary_Data

## Employment or Leadership

A. Silvestro, Novartis; J.C. Barrett, AstraZeneca; K. Bramlett, Thermo Fisher Scientific; D. Chudova, Guardant Health; I. Deras, Illumina; J. Dickey, Personal Genome Diagnostics; R. Leary, Novartis Pharmaceuticals; A. Richardson, Guardant Health; M. Ryder, Sysmex Inostics, Inc.; J. Simmons, Personal Genome Diagnostics; L.C. Leiman, Blood Profiling Atlas in Cancer (BloodPAC); Jim Godsey, Illumina; James Hicks, University of Southern California; Donald J. Johann, University of Arkansas; Jerry S.H. Lee, University of Southern California; Joe McMullen, Illumina; Lisa McShane, National Cancer Institute at the National Institutes of Health (NIH/NCI); Katherine Nakamura, Thermo Fisher Scientific; Matthew Ryder, Sysmex Inostics; Kelli Tanzella, Thermo Fisher Scientific; Laura Yee, National Cancer Institute at the National Institutes of Health (NIH/NCI).

## Stock Ownership

A. Silvestro, Novartis; J.C. Barrett, AstraZeneca; K. Bramlett, Thermo Fisher Scientific; D. Chudova, Guardant Health; I. Deras, Illumina (ILMN); J. Hicks, Epic Sciences, Inc.; R. Leary, Novartis; A. Richardson, Guardant Health; J. Simmons, Personal Genome Diagnostics.

## Honoraria

None declared.

## Expert Testimony

None declared.

## Patents

R. Leary, 20180230550.
