# Supplementary material for: Generic Protocols for the Analytical Validation of Next-Generation Sequencing-Based ctDNA Assays: A Joint Consensus Recommendation of the BloodPAC’s Analytical Variables Working Group
Source: Clin Chem. 2020 Sep 1;66(9):1156–66. doi: 10.1093/clinchem/hvaa164 (PMC7462123; doi:10.1093/clinchem/hvaa164)
Supplement: hvaa164_Supplementary_Data [file hvaa164_supplementary_data.zip › hvaa164-suppl_data/Supplemental Material 1.pdf]

***Generic Analytical Validation Protocols for Cell Free DNA Assay Performance Verification* – Version 1.0**

Submission Number: Q181500

This document provides a core set of generic protocols that will serve as the starting point for analytical validation studies designed to establish the analytical performance of a newly developed cell free assay for use in the clinical setting.

BloodPAC
info@bloodpac.org | © 2017 BloodPAC
BloodPAC is a consortium managed by the Center for Computational Science Research, Inc., which is an Illinois based not-for-profit corporation

BloodPAC
BLOOD PROFILING ATLAS IN CANCER

The Blood Profiling Atlas in Cancer (Blood PAC) Consortium was launched on October 17, 2016 to accelerate the development and validation of liquid biopsy assays to improve the outcomes of patients with cancer.

To do so Blood PAC will develop a collaborative infrastructure that enables sharing of information between stakeholders in industry, academia, and regulatory agencies.

Blood PAC is a consortium managed by the Center for Computational Science Research, Inc. (CCSR), which is an Illinois based not-for-profit corporation.

Blood PAC Goals

The goals of Blood PAC are: to aggregate, make freely available, and harmonize for further analysis: i) data from CTC (circulating tumor cells), ctDNA (circulating tumor DNA), proteins including tumor associated autoantibodies, and exosome assays, ii) associated clinical data, such as clinical diagnosis, treatment history and outcomes and iii) sample collection, preparation and handling protocols.

The BloodPAC Consortium includes representatives from academia, private foundations, industry and the government that are working to accelerate the exploration, implementation and assessment of potential clinical utility of liquid biopsies with the aim to understand the temporal evolution of a patient's disease.

BloodPAC Mandate

Our mandate is to accelerate the development and validation of liquid biopsy assays to improve the outcomes of patients with cancer. To do so we will develop a collaborative infrastructure that enables sharing of information between stakeholders in industry, academia, and regulatory agencies.

Get Involved

The BloodPAC consortium is committed to open and active membership and considers new applications on a rolling basis. If you would like more information, please contact us. If you are interested in joining the BloodPAC, you can find membership related materials here.

BloodPAC Leadership

Lauren Leiman; Executive Director, BloodPAC Consortium

BloodPAC Co-Chairs

- Darya Chudova – Guardant Health
- Ryan Dittamore – Epic Sciences
- Jim Godsey – Illumina, Inc.
- Robert L. Grossman - University of Chicago, Open Commons Consortium
- Peter Kuhn - University of Southern California
- Jerry S.H. Lee - University of Southern California, Ellison Institute for Transformative Medicine
- Anne-Marie Martin – Novartis
- Howard Scher - Memorial Sloan Kettering Cancer Center
- John Simmons - Personal Genome Diagnostics
- Jake Vinson - Prostate Cancer Clinical Trials Consortium
- Hakan Sakul - Pfizer, Inc.

BloodPAC
info@bloodpac.org | © 2017 BloodPAC
BloodPAC is a consortium managed by the Center for Computational Science Research, Inc., which is an Illinois based not-for-profit corporation
.

Proposed: Generic Analytical Validation Protocols for Cell Free Assay Performance Verification – Version 1.0

1. Abstract

BloodPAC' s Version 1.0- Generic Analytical Validation Protocols for Cell Free Assay Performance Verification provides a complete set of generic protocols designed to provide test developers/manufacturers with a core baseline of standardized analytical validation protocols with which to document a cell free assay's analytical performance. Revision 3.0 is intended for use by developers/manufacturers of NGS-based ctDNA diagnostic tests for oncology, regulatory bodies and clinical laboratories.

BloodPAC's revision 1.0, 2.0, 3.0, and 4.0 - Generic Analytical Validation Protocols for Cell Free Assay Performance Verification will be made available upon request by visiting the www.bloodpac.org website.

Copyright 2019 BloodPAC. Except as stated below, any reproduction of content from a BloodPAC copyrighted document requires express written consent from BloodPAC. All rights reserved. Interested parties may send permission request to permissions@bloodpac.org.

Proposed Guideline

February 2020

BloodPAC
info@bloodpac.org | © 2017 BloodPAC
BloodPAC is a consortium managed by the Center for Computational Science Research, Inc., which is an Illinois based not-for-profit corporation
.

2. Analytical Variables Working Group Membership

James H. Godsey
Vice President,
Assay Development
Illumina, Inc.
Chairholder

Angela Silvestro
Principle Scientist
Precision Medicine
Novartis
Co-Chairholder

J. Carl Barrett
Vice President,
Oncology Translational Sciences
AstraZeneca

Kelli Bramlett
Director, R&D
Clinical Sequencing Division
Life Science Solutions
Thermo Fisher Scientific

Darya Chudova
VP, Technology
Guardant Health

Ina Deras
Staff Scientist
Illumina

Jennifer Dickey
Vice President
Regulatory Affairs
PGDx

Ryon Graf
Senior Biomarker Scientist
Translational Research
EPIC Sciences

James Hicks
Research Professor
Department of Biological Sciences
Dornsife College of Letters, Arts, and Sciences
USC

Jennifer Jackson
Associate Director
Clinical NGS
PGDx

Donald Johann
Associate Professor
Biomedical Informatics, Hematology/Oncology
Scientific Director of the UAMS Genomics Facility
University of Arkansas for Medical Science

Rebecca Leary
Lab Head/Senior Investigator
Next Generation Diagnostics at Novartis Institutes for BioMedical Research (NIBR)
Novartis

Jerry S.H. Lee
Associate Professor of Clinical Medicine and Chemical Engineering
Chief Scientific and Innovation Officer
Ellison Institute
USC

Lauren Leiman
Executive Director
Blood PAC

Joe McMullen
Senior Director
Regulatory Affairs
Illumina

Lisa McShane
Associate Director
Division of Cancer Treatment & Diagnosis
Biometric Research Program
NIH/NCI

Katherine Nakamura
Principle Scientist
Biostatistics
Thermo Fisher Scientific

BloodPAC
info@bloodpac.org | © 2017 BloodPAC
BloodPAC is a consortium managed by the Center for Computational Science Research, Inc., which is an Illinois based not-for-profit corporation
.

Aaron Richardson
Staff Bioinformatics Scientist
Guardant Health

Kelli Tanzella
Senior Director, Global Regulatory Affairs, Clinical & Compliance
Life Sciences Solutions
Thermo Fisher Scientific

Matthew Ryder
Associate Director
Companion Diagnostics
Sysmex-Inostics

Laura Yee
Mathematical Statistician
Biometric Research Program, DCTD
NIH/NCI

John Simmons
VP, Translational Medicine
PGDx

BloodPAC
info@bloodpac.org | © 2017 BloodPAC
BloodPAC is a consortium managed by the Center for Computational Science Research, Inc., which is an Illinois based not-for-profit corporation
.

CONTENTS

1. Abstract 4

2. Analytical Variables Working Group Membership 5

Section I: INTRODUCTORY REMARKS 11

1. FOREWORD: Why Create a Set of Generic Analytical Validation Protocols for cfDNA Assays? 11

2. SCOPE: 15

3. Standard Precautions: 15

4. TERMINOLOGY: 15

5. BACKGROUND 17

5.1. Historical perspectives 17

5.2. Overview of NGS Detection Capability 17

5.3. Importance of high analytical sensitivity for clinical ctDNA testing 17

5.4. Clonal Hematopoiesis of Indeterminate potential (CHIP) 18

5.5. Specimen Collection Tubes 19

5.6. 21st Century Cures Act 20

Section II: ANALYTICAL VALIDATION PROTOCOLS 21

1. Protocols for the Evaluation of A Reference Interval 21

1.1. Introduction 21

1.2. Experimental Protocols 22

1.3. Minimal Experimental Design (For Variants with Quantitative Claims) 22

1.4. Statistical Analysis 22

1.5. Data presentation Format 22

2. Protocols for Evaluation of Limit of Blank (LoB) 23

2.1. Introduction 23

2.2. Experimental Protocols 24

2.2.1. Option A: Establish LoB (for Variants with Quantitative Claims) 24

2.2.2. Option B: Set LoB to Zero (for Variants with Qualitative Claims) 24

2.3. Minimal Experimental Design 25

2.4. Statistical Analysis 25

info@bloodpac.org | © 2017 BloodPAC
BloodPAC is a consortium managed by the Center for Computational Science Research, Inc., which is an Illinois based not-for-profit corporation

Non-parametric Method (FOR VARIANTS WITH QUANTITATIVE CLAIMS) 25
Parametric Method (for Variants with Quantitative Claims) 25
Non-Parametric Method (for Variants with Qualitative Claims) 25
Other considerations 26
2.5. Data Presentation Format 26
3. Protocols for Contrived Sample Functional Characterization Studies 26
3.1. Introduction 26
3.2. Experimental PROTOCOLS (FOR VARIANTS WITH Qualitative CLAIMS) 28
3.3. Statistical ANALYSIS (FOR VARIANTS WITH Qualitative CLAIMS) 29
4. Protocols for EVALUATION OF LIMIT OF DETECTION (LOD) 32
4.1. Introduction 32
4.2. Experimental Protocols: Preparation of Contrived Samples 33
4.3. Experimental Protocols: Evaluation of Limit of Detection (LoD) 34
4.4. Minimal Experimental Design 34
4.5. Minimal Experimental Design: probit Regression Model 34
4.6. Statistical Analysis using the Parametric and Non-Parametric Models for Variants with Quantitative Claims 35
Probit model LoD calculation for variants with Qualitative Claims 35
4.7. Data Presentation Format 35
4.8. Experimental Protocols: Confirmation of LoD 36
4.9. Statistical Analysis: Confirmation of LoD 37
4.10. Data Presentation Format: Confirmation of LoD 37
5. Protocols for Evaluation of Analytical Accuracy 37
5.1. Introduction 37
5.2. Statistical Analysis for Variants with Quantitative Claims 39
5.3. Statistical Analysis for Variants with Qualitative Claims 39
5.4. Data Presentation Format 40
6. Protocols for Evaluation of Linearity 40
6.1. Introduction 41
6.2. Experimental Protocols: Preparation of Contrived DNA 41
6.3. Experimental Protocols: Evaluation of Linearity 41

BloodPAC
info@bloodpac.org | © 2017 BloodPAC
BloodPAC is a consortium managed by the Center for Computational Science Research, Inc., which is an Illinois based not-for-profit corporation
.

6.4. Statistical Analysis 42
6.5. Data Presentation Format 42
7. Protocols for Evaluation of Limit of Quantitation (LoQ) 43
7.1. Introduction 43
7.2. Minimal Experimental Design: preparation of Contrived samples 43
7.3. Minimal Experimental Design: Evaluation of LoQ 44
7.4. Statistical Analysis 44
7.5. Data Presentation Format 44
8. Protocols for Evaluation of Repeatability, Precision and Reproducibility 44
8.1. Introduction 44
8.2. Experimental Protocols: REPRODUCIBILITY 45
8.3. Experimental Protocols: INTERMEDIATE PRECISION 46
8.4. Statistical Analysis for Variants with Quantitative Claims 46
8.5. Statistical Analysis for Variants with Qualitative Claims 47
11.5. Data Presentation Format 47
9. Protocols for Evaluation of Interfering Substances 47
9.1. Introduction 47
9.2. Experimental Protocols: Sources of Interference 48
Endogenous and Exogenous Interfering substances 48
9.3. Minimal Experimental Design 48
9.4. Statistical Analysis 48
9.5. Data Presentation Format 49
10. Protocols for Guardbanding Class III Medical Devices 50
10.1. Introduction 50
10.2. Experimental Protocols: Identifying Variables 50
10.3. Minimal Experimental Design 51
10.4. Statistical Analysis 51
10.5. Data Presentation Format 51
11. Protocols for Evaluation of Prepared Specimen Stability 52
11.1. Introduction 52
11.2. Experimental Protocols 53

BloodPAC
info@bloodpac.org | © 2017 BloodPAC
BloodPAC is a consortium managed by the Center for Computational Science Research, Inc., which is an Illinois based not-for-profit corporation
.

11.3. Statistical Analysis 53
11.4. Data Presentation Format 53
1. Preparation of Contrived samples using cfDNA from cell culture media 55
1.1. Objective of this Standard Method 55
2. Preparation of Contrived DNA Samples using Fragmented Cell line Genomic DNA 56
2.1. Objective of this Standard Method 56
3. Preparation of AcroMetrix™ Oncology Hotspot Control Reference material 58
3.1. Objective of this Standard Method 58
3.2. Standard Protocol: Preparation of Contrived Samples for Lung cfDNA Assay Development | SNVs 58
3.3. Standard Protocol: Preparation of Contrived Samples for lung cfDNA Assay Development | FUSIONS 59
3.4. Standard Protocol: Preparation of Contrived Samples for lung cfDNA Assay Development | CNVs 59
4. Standard Method for Collecting Normal Human Plasma 59
4.1. Objective(s) for this Standard Method 59
4.2. Standard Protocols 60
5. Standard Method for the Preparation of Patient Sample Pools 60
SECTION IV: REFERENCES CITED 62
Appendix A: Worked Example: 65
Appendix B: Summary Table of Example experimental Designs across protocols 68
Appendix C: Targets Included in AcroMetrix® Oncology Hotspot Control 72

BloodPAC
info@bloodpac.org | © 2017 BloodPAC
BloodPAC is a consortium managed by the Center for Computational Science Research, Inc., which is an Illinois based not-for-profit corporation

SECTION I: INTRODUCTORY REMARKS

1. FOREWORD: WHY CREATE A SET OF GENERIC ANALYTICAL VALIDATION PROTOCOLS FOR CFDNA ASSAYS?

One of the central objectives of the BloodPAC is the standardization and streamlining of Pre-analytical, Analytical and Clinical studies required for new cfDNA Assays, to enable FDA clearance/approval. In this document, the Analytical Variables Working Group takes on the challenge of defining a set of generic protocols for the *Analytical Validation of cfDNA Assays*.

In developing a set of ***generic*** *protocols, one must accept the fact that these were written in the absence of a specific intended use and clinical context for the assay.* ***Agreement on this assumption is critical for establishing a mindset of review of the subsequent material found in this document***.

The ***"Generic Intended Use"*** of these protocols is toward assays for patients previously diagnosed with solid tumor cancers. Specifically, these protocols were designed for assays intended for treatment decision making; that is, detection of relevant biomarkers for treatment and/or monitoring. This excludes assays for **screening** or early cancer diagnosis. The intent is for use by qualified health care professionals in accordance with professional guidelines. Developers using these protocols should determine the appropriate level of analytical and clinical validation that is consistent with the Intended Use of their specific assay or device.

Developers who seek a quantitative claim for any variants for MAF reporting and/or monitoring may receive such claims for specific variant types and/or biomarkers, and not for the entire panel. In addition, for a monitoring claim, assay developers should demonstrate not only high sensitivity (LoD), but will also need to establish the assay's lower limit of quantitation (LoQ) to demonstrate the assay's ability to distinguish biological changes in the level of ctDNA in circulation from technical variation and measure the ctDNA level with sufficient accuracy. A prospective clinical validation study with the final device in the appropriate intended use population will be required to demonstrate a monitoring claim for your device. Please also note that CDRH will consult with CDER for acceptability of the clinical protocol(s).

It is acknowledged that highly multiplexed NGS panels may include variants with a range of clinical evidence supporting each. New test developers should refer to the FDA's current thinking on Level 1, 2, 3, and 4 claims to determine the evidence requirements for the different levels of claims for ctDNA-based biomarkers. These

BloodPAC
info@bloodpac.org | © 2017 BloodPAC
BloodPAC is a consortium managed by the Center for Computational Science Research, Inc., which is an Illinois based not-for-profit corporation

protocols were developed based on current thinking in the liquid biopsy community, which is evolving rapidly. The sponsor is strongly encouraged to work with FDA on finalizing the protocols including sample numbers and types, variant types, acceptance criteria, and statistical analysis plan to support the device Intended Use.

Secondly, one must understand that the intent of Analytical Validation is to determine the performance limits and robustness of an assay that has been developed under *design control* and has completed the process of design lock, assay verification and validation and is now going through final "clinical validation testing" which includes both a set of specific analytical validation protocols and clinical protocols. The data from these studies make up the data set submitted to the FDA for review of regulatory approval.

Having stated as much, it is important to mention that there is a myriad of technologies (e.g. extraction chemistries, blood collection tubes, sequencing), and assay components/raw materials, concentrations, formulations and bioinformatics algorithms/pipelines that are available to researchers and developers to design and develop a liquid biopsy assay. It is understood that finalizing the selection of these technologies/components is an action completed at the "design output" step of *design control*, and thus well before encountering these proposed "generic analytical protocols". A key component of any NGS assay, that is in many ways unique to NGS, is the bioinformatics (BxF) *"analytics pipeline"* which consists of software (SW) code and embedded BxF algorithms that ensure the accuracy of nucleotide base calling and variant calling. In addition, there are typically algorithms to generate "quality" scores, Quantity Not Sufficient (QNS) cutoffs or Assay Validity/Invalidity scoring to determine if an NGS final result should in fact be reported as valid and appropriate for the end users' purpose, including patient result reporting. It is understood that this SW code and BxF algorithms are also finalized prior to the assay being challenged by the generic analytical validation protocols proposed by this guidance document.

Therefore, the intent of creating the generic set of analytical validation protocols that follows is solely for the purpose of demonstrating and documenting assay performance, independent of technology or design.

BloodPAC
info@bloodpac.org | © 2017 BloodPAC
BloodPAC is a consortium managed by the Center for Computational Science Research, Inc., which is an Illinois based not-for-profit corporation

A. Finalization of Preanalytical MTDE's– *completed*.

B. Formal Assay Development under Design Control

1. BCT
2. cfDNA Extraction Kits & Techniques
3. Library Building Approaches
4. Sequencing Instrumentation Platforms
5. Bioinformatics Pipelines & Analyses

C. Generic Analytical Validation

D. Formal Clinical Validation of cfDNA Assay

Figure 1 The Role of the Generic Analytical Validation Protocol, the Salient Steps, and Context towards Clinical Utility for cfDNA Assays. Adapted from: Merker, Jason D., et al. "Circulating Tumor DNA Analysis in Patients with Cancer: American1.a.i.30 Society of Clinical Oncology and College of American Pathologists Joint Review." Archives of pathology & laboratory medicine (2018) [32]. "MTDE" indicates "Minimal Technical Data Element", a term used by the BloodPAC Data Commons.

Finally, the analytical validation of cell free assays poses a number of unique challenges, many of which stem from the fact that the amount of target DNA or RNA being detected by a cell free assay may be as small as 2-3 molecules, each 170 bp in length. Due to biological and technical differences, the use of tissue specimens for the validation of cfDNA-based assays is not appropriate. Because obtaining a sufficient quantity of patient-derived cfDNA may be difficult, the practice and use of ***contrived samples*** is far more common in analytical validation protocols involving cell free assays than for solid tumor assays. The AV WG has made ample use of contrived samples in its generic protocols that follow this *Foreword* but also clearly understands the need to arrive at an extremely solid foundational method by which all cell free assay developers can demonstrate the functional comparability of their contrived samples in a straight forward and reasonable manner to the FDA, as part of demonstrating their assay's overall performance and robustness. However, clinical samples from the intended use population should be used for key analytical validation studies (e.g., LoD confirmation, accuracy, precision, some stability studies, etc.).

BloodPAC
info@bloodpac.org | © 2017 BloodPAC
BloodPAC is a consortium managed by the Center for Computational Science Research, Inc., which is an Illinois based not-for-profit corporation

An additional unique challenge posed to NGS-based, cell free DNA assays discussed below is the issue of the clonal hematopoiesis of indeterminate potential (CHIP), which may lead to the detection of mutations in the plasma of healthy, aging individuals. Although CHIP is especially impactful in the reporting of screening assays used for early cancer detection (beyond the scope of this document), it may affect the false positive rate of any plasma-based assay [44]. Therefore, CHIP should be considered when planning the Reference Interval and LoB studies, as highlighted in the methods that follow, as well as the design of secondary analysis pipelines used to filter out such results.

The protocols presented in this document are divided into two sections: a) *Analytical Validation protocols*, and b) *Standards Methods*.

- *The Analytical Validation protocols have been written in a standard format:* i) introduction (definition and description of protocol's purpose), ii) generic analytical validation protocol, iii) statistical method, iv) presentation of data format.
- *The Standard Methods have been written in a standard format:* i) objective(s) of standard method, ii) Standard protocol, iii) footnotes.

The AV WG considers the work that follows as a baseline, which cfDNA assay developers/manufacturers can utilize in their initial pre-submission discussions with the FDA. The benefit is expediting the path to a set of agreed protocols in pre-submission discussions between test developers and FDA. The work also benefits the FDA in that it ***standardizes*** conversations with cfDNA Assay developers/manufacturers and thus makes the interaction between agency and manufacturer significantly more efficient, thus saving time and capacity for the limited number of FDA reviewers.

Standard NGS assay developers/manufacturers and academic centers have participated in the development of the platform and tools available in precisionFDA (https://precision.fda.gov/), which is a community-oriented research platform for NGS software evaluation. While these tools can be used to benchmark the performance of a bioinformatics pipeline using a known reference sample, cfDNA NGS assay developers typically encounter two additional challenges: (a) the introduction of proprietary barcoding or other assay methods optimized for cfDNA necessitates custom pipelines that may or may not be compatible across data sets generated by different platforms and (b) the definition of a gold standard for reference material has not yet been developed to the level that was achieved in other applications. Thus, these guidelines streamline validation methods that allow combined assessment of assay preparation and bioinformatics analysis within a single diagnostic test.

BloodPAC
info@bloodpac.org | © 2017 BloodPAC
BloodPAC is a consortium managed by the Center for Computational Science Research, Inc., which is an Illinois based not-for-profit corporation

2. SCOPE:

This document provides a framework for *generic* analytical validation protocols and standard methods that relate specifically to the evaluation and verification of assay performance claims of clinical laboratory measurement protocols (e.g. Limit of Blank [LoB], Limit of Detection [LoD], Precision [Repeatability and Reproducibility], Guardbanding studies for Class III Medical Devices, Interfering Substances etc.). This document is suitable for IVDs which are run in single or multiple laboratories controlled by the same company and for distributable kits, based on ***Next Generation Sequencing*** technology.

3. STANDARD PRECAUTIONS:

Since it is often impossible to know what isolates or specimens might be infectious, all patient and laboratory specimens are treated as infectious and handled according to "standard precautions." Standard precautions are guidelines that combine the major feature of "universal precautions and body substance isolation" practices. Standard precautions cover the transmission of all known infectious agents and thus are more comprehensive than universal precautions, which are intended to apply only to transmission of blood-borne pathogens. The Centers for Disease Control and Prevention address this topic in published guidelines that focus on the daily operations of diagnostic medicine in human and animal medicine while encouraging a culture of safety in the laboratory [34]. For specific precautions for preventing the laboratory recommendations for the management of exposure to all infectious diseases, refer to CLSI document M29 [2].

4. TERMINOLOGY:

a. Note on Terminology
   Unless otherwise noted, this document follows standard CLSI terminology.
b. Definitions:
   Note: Unless clarified, the definitions utilized in this document follow standard CLSI terminology.
c. Acronyms and Abbreviations

AF	Allelic Fraction
AMP	Association for Molecular Pathology
APA	Average Positive Agreement
ANA	Average Negative Agreement
AV WG	Analytical Variables Working Group

BloodPAC
info@bloodpac.org | © 2017 BloodPAC
BloodPAC is a consortium managed by the Center for Computational Science Research, Inc., which is an Illinois based not-for-profit corporation

BCT	Blood Collection Tube
bp	Base Pair
BxF	Bioinformatics
CAP	College of American Pathologists
CCSR	Center for Computational Science Research, Inc.
cfDNA	Cell Free DNA
cfRNA	Cell Free RNA
CHIP	Clonal Hematopoiesis of Indeterminate Potential
CLSI	Clinical and Laboratory Standards Institute
CNV	Copy Number Variant
cp	Copy
CRC	Colorectal Cancer
ctDNA	Circulating Tumor DNA
dPCR	Digital PCR
Dx	Diagnostic
FFPE	Formalin-Fixed Paraffin Embedded
FNR	False Negative Rate
FP	False Positive
FPR	False Positive Rate
IASLC	International Association for the Study of Lung Cancer
Indel	Insertion or Deletion
IVD	In Vitro Diagnostic Device
K2EDTA	Dipotassium Ethylene Diamine Tetraacetic Acid
LoB	Limit of Blank
LoD	Limit of Detection
LoQ	Limit of Quantitation
LBx	Liquid Biopsy
MAF	Mutant Allelic Fraction
MBL	Monoclonal B-cell lymphocytosis
MGUS	Monoclonal gammopathy of undetermined significance
MNV	Multinucleotide Variants
MRD	Minimal Residual Disease
MTDE	Minimal Technical Data Element
MUT	Mutant
N	Number
NCCN	National Comprehensive Cancer Network
ng	Nanogram
NGS	Next Generation Sequencing
NPA	Negative Percent Agreement
NSCLC	Non-Small Cell Lung Cancer

BloodPAC
info@bloodpac.org | © 2017 BloodPAC
BloodPAC is a consortium managed by the Center for Computational Science Research, Inc., which is an Illinois based not-for-profit corporation
.

Pos	Positive
PPA	Positive Percent Agreement
QNS	Quantity Not Sufficient
SD	Standard Deviation
SNV	Single Nucleotide Variant
SW	Software
TKI	Tyrosine Kinase Inhibitor
UMI	Unique Molecular Identifier
VAF	Variant Allele Frequency
WT	Wild Type

5. BACKGROUND

5.1. Historical perspectives

Tracing the history of cell free DNA (cfDNA), it was initially identified in the blood of healthy subjects by Mandel and Metais in 1948 [3]. There was not much interest in this finding until about 30 years later when it was reported that increased amounts of cfDNA were found in individuals with cancer [4]. A decade later, the presence of neoplastic characteristics in the circulation was reported [5]. Studies relating burden of disease and cfDNA first appeared in 2001, where it was reported that as the tumor size increases, the cellular replacement processes and the quantity of cellular debris increases [6]. Then, in landmark studies conducted in 2008, investigators at Johns Hopkins showed that ctDNA levels in patients with colorectal cancer change in response to changes in tumor burden [7].

5.2. Overview of NGS Detection Capability

Basic science and clinical medicine are being transformed by NGS through its unique capabilities for highly multiplexed detection of low-frequency variants in heterogeneous tumors [8, 32, and 33]. However, biological and technical variables can render reliable detection at low allele frequencies challenging for NGS methods that have not been optimized for this purpose. This may pose a challenge, since, for example, a significant proportion of patients may have variants present <0.1%: e.g., 13% of RAS MAFs between 0.02-0.1% for mCRC, 15% EGFR MAFs are between 0.02-0.1% for NSCLC, and 22% PIK3CA MAFs are observed between 0.02-0.1% for HR+/HER- recurrent breast cancer [21, 22, 23, 24]. In clinical situations involving liquid biopsies for biomarker applications in cancer, robust analytical performance is essential for NGS-based diagnostics and to ensure the quality and reliability of data, which may be used for medical decision-making.

5.3. Importance of high analytical sensitivity for clinical ctDNA testing

BloodPAC
info@bloodpac.org | © 2017 BloodPAC
BloodPAC is a consortium managed by the Center for Computational Science Research, Inc., which is an Illinois based not-for-profit corporation

Robust clinical evidence has now been generated for ctDNA analysis alongside, and in certain situations in place of, tissue analysis for advanced non-small cell lung cancer (NSCLC) patients who have progressed on first-line *EGFR* tyrosine kinase inhibitor (TKI) therapy. Resistance in this setting is known to be driven by a secondary mutation in *EGFR*, T790M, which presents in up to 60% of patients [9]. Although tissue is the preferred specimen type for *EGFR* molecular analysis for NSCLC, tissue testing in this context presents significant challenges ranging from complications associated with intrathoracic biopsy [10, 11] to failure of genomic profiling at a single local biopsy site to capture T790M-positive cells that are driving resistance/ progression and the turn-around time often associated with tissue testing. Because ctDNA analysis has the potential to overcome these challenges, it would be ideally suited for *EGFR* T790M detection; however, a test with this intended use must demonstrate high analytical sensitivity since a substantial proportion of patients exhibit T790M at very low allele frequency (0.1-0.5%) in the blood.

Based on data generated by Oxnard *et al.* [12], which demonstrate equivalent outcomes for NSCLC patients treated with osimertinib who are plasma-positive for T790M and patients who are tissue-positive, NCCN guidelines now recommend plasma testing for T790M for NSCLC patients who have progressed on a first or second-generation *EGFR* TKI [13]. The recent update to joint CAP/ AMP/ IASLC clinical practice guidelines for NSCLC also includes this recommendation [14]. Both sets of guidelines recommend reflex to tissue testing if T790M is not detected via plasma analysis; therefore, use of a well-validated, accurate ctDNA assay with high analytical sensitivity and specificity are necessary to decrease risk and cost associated with tissue biopsy for the greatest number of patients.

Clinical intended uses for ctDNA with emerging evidence include assessment of minimum residual disease (MRD) following a therapeutic regimen and/or surgical intervention, as well as monitoring response to various targeted and systemic therapies across different types of cancer. For these, as well as other potential clinical uses for ctDNA analysis, robust analytical performance, including both high sensitivity and specificity, is the first step towards reliable liquid biopsy tests that can demonstrate health economic value. In addition, as stated above, developers who seek monitoring claims should demonstrate high sensitivity (LoD) and will also need to establish the LoQ for their assay. Mutation calling/ reporting thresholds should be set based on an assay's demonstrated limit of detection to ensure reproducibility of biomarker data across various clinical intended use populations. "Sub-threshold" calling is generally not considered an acceptable practice due to the elevated false negative rate (FNR)/false positive rate (FPR) which may lead to inconsistent treatment of patients.

5.4. Clonal Hematopoiesis of Indeterminate potential (CHIP)

Clonal Hematopoiesis of Indeterminate Potential (CHIP) is a major issue for any liquid biopsy test in that it may lead to the detection of mutations in otherwise healthy, aging

BloodPAC
info@bloodpac.org | © 2017 BloodPAC
BloodPAC is a consortium managed by the Center for Computational Science Research, Inc., which is an Illinois based not-for-profit corporation

individuals. Due to CHIP, the number of mutations in white blood cells may be significantly expanded in the aging populations, creating an opportunity to detect a false positive liquid biopsy result that suggests a cancer driver gene may be active, when in fact it is not [17]. This increased false positive rate due to CHIP may impact the ability of the test to detect alterations that are accurate, reliable, and clinically meaningful. In addition, these CHIP variants detected in plasma may contribute to the discordance observed between tissue and plasma-based assays [17].

Factors to consider concerning CHIP:

1. Along with germline DNA, developers may also consider clonal hematopoiesis of indeterminate potential (CHIP), which is analogous to MGUS, a benign but potential precursor state to Multiple Myeloma. Note: The diagnosis of CHIP like MGUS does not mean that a person has cancer. CHIP should be addressed since it will likely influence (e.g., confounding variable) cfDNA assays.
2. The recommended approach to addressing false positives that may be caused by CHIP is to sequence matched white blood cells at the same depth as plasma. However, depending on the assay's intended use, developers may choose to address CHIP bioinformatically.
3. Recent large population genetic analyses concerning human aging have discovered that mutations in blood cells may introduce clonal expansion and occur frequently [27].
4. This process has been named clonal hematopoiesis of indeterminate potential (CHIP) and, importantly, does not involve dysplastic processes of cytopenias [28].
5. CHIP is similar to monoclonal gammopathy of undetermined significance (MGUS) and monoclonal B-cell lymphocytosis (MBL), which are predecessor conditions for multiple myeloma and chronic lymphocytic leukemia, respectively.
6. MGUS and MBL are usually benign and do not progress to a malignant state. Importantly, since mutations are commonly detected in healthy older adults (e.g. CHIP), the discovery of cancer related mutations via cfDNA might not indicate that the subject already has cancer, or will even develop cancer during their lifetime. Such results may just cause significant anxiety and possibly additional diagnostic procedures with radiation side effects (e.g., body CT).

5.5. Specimen Collection Tubes

Blood Collection Tubes (BCTs) are an important component of any ctDNA assay. It is recommended that new assay developers ensure that the selected BCT associated with the ctDNA assay is cleared or authorized concurrently or prior to the approval of the assay. Because of their importance as an integral part of the assay design, developers should select and evaluate BCTs appropriately for their intended use. Assay developers are recommended to identify and list the specifications of the BCTs required for the assay's intended use. As discussed in the Foreword to this document, the selection of an

BloodPAC
info@bloodpac.org | © 2017 BloodPAC
BloodPAC is a consortium managed by the Center for Computational Science Research, Inc., which is an Illinois based not-for-profit corporation

appropriate BCT should be made prior to starting the analytical validation protocols that follow. However, assay developers are responsible for the validation of the selected BCTs to demonstrate that the BCTs performance meets the specifications relevant to the assay's intended use and performance goal. It is also recommended that either the BCT manufacturer or the assay developer consider discussing BCT validation plans with the Division of Chemistry and Toxicology Devices (DCTD) prior to the start of analytical validation.

5.6. 21ST CENTURY CURES ACT

The 21st Century Cures Act (Cures Act), signed into law on December 13, 2016, is designed to help accelerate medical product development and bring new innovations and advances to patients who need them faster and more efficiently [16]. Principal aims are to bring treatments to market faster, especially in the realm of device and drug approvals. In this regard, liquid biopsy methodologies, for the purpose of plasma genotyping of cell free DNA (cfDNA) of solid tumors, are a new class of molecular assays. Such assays are rapidly being translated from the research space into clinical practice for oncology. Potential applications for these blood-based cfDNA assays include: i) selection of targeted therapies, ii) response to therapy and follow-up, iii) tumor evolution, and iv) minimal residual disease evaluation. Patients will benefit from advanced molecular diagnostics, especially concerning treatment decisions in the adjuvant setting, where avoiding over-treatment and unnecessary toxicity are paramount.

BloodPAC
info@bloodpac.org | © 2017 BloodPAC
BloodPAC is a consortium managed by the Center for Computational Science Research, Inc., which is an Illinois based not-for-profit corporation
.

SECTION II: ANALYTICAL VALIDATION PROTOCOLS

Overview: These protocols include an example design for each study. A suggested study design is described below. It is expected that individual sponsors will determine an appropriate sample size. For some studies (e.g. accuracy, etc.), the minimal sample size must be determined by the lower bound of the acceptance criteria. For other studies (e.g. LoB, LoD, etc.), that establish analytical performance based on point estimates or the performance goal of the assay, the minimal sample size should sufficiently address the uncertainty of the performance estimates to support the claims. See Appendix B: Summary Table of Minimal experimental Designs across protocols for a summary of all study designs.

1. PROTOCOLS FOR THE EVALUATION OF A REFERENCE INTERVAL

1.1. INTRODUCTION

The reference range or interval refers to the range of sequence variation and known polymorphisms that are found in the disease-free reference donor in the normal population that are detectable by the assay (CLSI MM09) [37]. Understanding this range in the normal population is important in determining whether the very low MAF variants detected by a cfDNA-NGS assay are clinically meaningful. Assay developers of NGS-based assays may decide to report all detected variants or may only report known, clinically relevant variants, depending on the assay's intended use (Jennings et al. 2017) [38]. In either case, the reference interval must be reported for the assay so that the results may be appropriately interpreted by the clinician or other user with an understanding of what variants may or may not have been reported (Jennings et. 2017)[38]. Selection of reference donors is critical to the validation of the reference range (CLSI C28-A3C) [1]. Selection should be based on the assay's intended use and should include disease-free donors that are otherwise representative of the patient population. For cfDNA-NGS-based assays, reference donors should be age- and risk-matched normal donors who have characteristics comparable to those of the intended use population and should include some young unmatched healthy individuals. Specimens from the selected reference donors should be collected and processed using the same conditions (e.g. blood collection tube, shipping and storage procedure, and cfDNA extraction method) used to handle patient samples in the locked assay design to control for preanalytical variables (CLSI C28-A3C) [1].

This consistency in processing of reference donors should also include the method of handling CHIP in the final device. As noted above, CHIP is a significant issue that may lead to the detection of mutations in healthy, aging individuals. Therefore, developers should plan to use the final CHIP subtraction/filtering method (through the sequencing of matched normal or by filtering out CHIP bioinformatically) that will be used in the final assay design

BloodPAC
info@bloodpac.org | © 2017 BloodPAC
BloodPAC is a consortium managed by the Center for Computational Science Research, Inc., which is an Illinois based not-for-profit corporation

in this study to determine the Reference Interval and in all other analytical and clinical validation studies.

It is recommended that assay developers calculate the reference range for each specific variant and within each variant class with a quantitative claim following the guidance CLSI C28-A3C, as described below [1]. Developers may utilize some normal donors selected for use in the LoB study in the reference interval study; however, these studies cannot entirely be combined since the LoB study also requires the use of mutation-negative samples from individuals with cancer. Because it may be difficult or impossible to find samples that are truly variant-free, for the purpose of these protocols, "mutation negative" will refer to samples that are wild type at each position of interest that may be reported as positive by the test. For variants with a qualitative claim, assay developers should utilize the LoB study data only and no reference interval will be reported.

1.2. Experimental Protocols

1. Select appropriate age-and risk-matched reference donors. Include some young, unmatched healthy individuals. This study may include, in part, normal donors who are also included in the LoB study (see below). Historical data collected from healthy subjects may also be included in the analysis to attain the minimum number of observations.
2. Process all reference donor plasma specimens using the same methods and procedures as patient samples.
3. Perform the minimum number of total observations listed below using the cfDNA-NGS assay to be validated.

1.3. Minimal Experimental Design (For Variants with Quantitative Claims)

A minimum of 120 observations. It is recommended that these observations come from 120 reference donors.

1.4. Statistical Analysis

The lower and upper reference limits are estimated as the 2.5th and 97.5th percentiles of the distribution of test results for the reference population, respectively (CLSI C28-A3C), and should be calculated for each variant in the panel with a quantitative claim. Samples with undetectable variants are considered to be at the lowest end of all test results.

1.5. Data presentation Format

Present the reference interval for each variant with a quantitative claim.

BloodPAC
info@bloodpac.org | © 2017 BloodPAC
BloodPAC is a consortium managed by the Center for Computational Science Research, Inc., which is an Illinois based not-for-profit corporation
.

2. Protocols for Evaluation of Limit of Blank (LoB)

2.1. Introduction

Limit of blank is established using blank samples and is used to control for type 1 error and thus forms the basis of an assay's specificity (see CLSI EP17)[16]. For cfDNA assays, blank samples represent "cancer biomarker-free nucleic acid samples." Such samples should be isolated from plasma obtained from age- and risk- matched normal donors who have characteristics comparable to those of the intended use population as well as from mutation-negative (wild type at each position that may be reported as positive by the test) individuals with cancer. This protocol is executed using the final device and bioinformatics pipeline, but ahead of any performance evaluation study (LoD, Accuracy, Precision etc.) as it provides the dataset needed to set variant detection thresholds and thus control for type 1 error (false positives). In the context of a LoB study using blank samples, any positive signal would be considered a false positive call. For example, MAF (for SNVs) or number of reads (for Fusions/Structural variants), may be examples of positive signal. For this reason, for molecular tests, it may also be appropriate to the set the LoB equal to zero and then to run a number of blank samples to confirm that no false positives are detected.

As discussed above in the Reference Interval section, CHIP is a significant issue that may lead to the detection of mutations in healthy, aging individuals. Therefore, CHIP may affect LoB of the assay. As in the Reference Interval study, assay developers should test samples in the LoB study in a method that reflects how the final device will handle CHIP. If it is sequencing for CHIP with subtraction through matched WBC sequencing), then the same approach should be used to determine Reference Interval and LoB and for all other validation studies.

As discussed above in the Reference Interval section, CHIP is a significant issue that may lead to the detection of mutations in healthy, aging individuals. Therefore, CHIP may affect LoB of the assay. As in the Reference Interval study, assay developers should test samples in the LoB study in a method that reflects how the final device will handle CHIP. For example, if sequencing for CHIP with subtraction through matched WBC sequencing is part of the final device, then the same approach should be used to determine Reference Interval and LoB, and subsequent analytical and clinical validation studies to demonstrate the performance of the assay.

Alternatively, rather than empirically establishing LoB, one can choose to set the LoB to zero, and then run blank samples to simply confirm performance (see CLSI EP17) [16].

Two example study designs (Option A and B) are described below for variants with quantitative and qualitative claims, respectively. These designs have been provided as examples. The minimal sample size should be determined for each assay based on its performance goal the level to which the false positive rate of the assay must be controlled for its intended use. For qualitative assays, the performance goal is a FPR (false positive

info@bloodpac.org | © 2017 BloodPAC
BloodPAC is a consortium managed by the Center for Computational Science Research, Inc., which is an Illinois based not-for-profit corporation

rate) close to zero. In selecting blank samples for this study, using only normal donors as described in the Reference Interval study above is not sufficient. Mutation-negative (wild type at each position of interest) samples from both normal donors and individuals with cancer should be included in the LoB study. Samples could be pre-screened using an orthogonal method before use in the LoB study and the results of study samples should be used to determine the FPR.

Please note that the LoB should be established for all variant types detected by the assay, using either Option A or Option B, depending on the assay claims and how the results will be reported. Note that for qualitative variants with a clinical qualitative indication, the positive results should be defined consistently with the definition of positive call in the clinical setting. Therefore, the false positive rate should be calculated using the clinical cutoffs.

2.2. Experimental Protocols

2.2.1. Option A: Establish LoB (for Variants with Quantitative Claims)

1. Panel of at least 60 total blank samples derived from a combination of age-appropriate normal donors and mutation-negative individuals with cancer that can be pre-screened using an orthogonal method.
2. Test all samples at an input that is at the high end of the assay's input requirements.
3. 2 separately manufactured reagent lots
4. LoB is typically defined as the 95th percentile of signal from blank samples.
5. To target <1% false positive rate per variant, the detection thresholds should be set at > 99th percentile of signal obtained from the blank samples.

2.2.2. Option B: Set LoB to Zero (for Variants with Qualitative Claims)

1. Panel of at least 60 total blank sample (per CLSI EP17) derived from a combination of age- and risk- matched normal donors who have characteristics similar to the intended use population and mutation-negative individuals with cancer.
2. Test all samples at an input that is at the high end of the assay's input requirements.
3. 2 separately manufactured reagent lots
4. Acceptance criteria: The FPR should be approximately zero for all levels.
   Note that the purpose of using an orthogonal method is to identify blank samples to be evaluated in the LoB study. This is to ensure that mutation positive samples are not evaluated in the LoB study since the purpose of the LoB study is to demonstrate FPR of the assay using blank samples, i.e., true mutation negative

samples. Variants above the detection threshold should NOT be verified post-hoc after running the LoB study.

5. The false positive rate should be reported as follows:
   - On a per variant basis, both for hotspots and panel-wide variants
   - On a per sample basis
   - In addition, the false positive rate should be calculated separately for both the normal donors and mutation-negative individuals with cancer used in this study.

2.3. Minimal Experimental Design

1. At least 2 independent reagent lots
2. An appropriate number of independent normal mutation-negative sample from individuals with cancer for each reagent lot to attain a total of 60 blank tests per reagent lot
3. At least 2 replicates for each independent sample run within a reagent lot
4. A total of at least 120 measurements (2 x 60)

2.4. Statistical Analysis

Depending upon the distributions of the blank sample results, a nonparametric, or more rarely, a parametric data analysis option is selected to calculate the LoB estimates.

Non-parametric Method (FOR VARIANTS WITH QUANTITATIVE CLAIMS)

1. Sort all B blank sample measurement results from lowest to highest; B is defined as the total number of blank results in the dataset: X (1), X (2), X (B), per CLSI EP17.
2. Calculate the pth percentile for the distribution of blank sample results (PctB) corresponding to the desired alpha (typically, 0.05) level and p=1-alpha=0.95, 95Pct.
3. Calculate the rank position corresponding to the PctB percentile as 0.5+0.95*B.
4. LoB is estimated as the value of the measurement result at the calculated rank position.

Parametric Method (for Variants with Quantitative Claims)

1. Calculate the mean (MB) and SD (SDB) of all blank sample results
2. Calculate the LoB as MB+Cp*SDB; Cp*SDB; where Cp is a multiplier to give the 95th percentile of a normal distribution, B is the total number of blank results, and K is the total number of blank samples.

Non-Parametric Method (for Variants with Qualitative Claims)

BloodPAC
info@bloodpac.org | © 2017 BloodPAC
BloodPAC is a consortium managed by the Center for Computational Science Research, Inc., which is an Illinois based not-for-profit corporation
.

For qualitative claims, the non-parametric method can be selected to confirm LoB. For example, for an alpha=0.01, 99% of calls should yield no signal.

OTHER CONSIDERATIONS

1. LoB estimate is calculated for each reagent lot. The maximum observed LoB across all reagent lots or the LoB estimate for combined data of all reagent lots is taken as the reported value for the measurement procedure.
2. Although setting an alpha of 0.05 is typical for determining LoB, it is recommended for developers to set a more stringent alpha at 0.01 in this protocol to optimize specificity and to reduce the risk of false positives on a per variant or per sample basis. However, assay developers should be aware that if they plan to determine false positives across the reportable space of their NGS panel (for example, per kb of target sequence), then an even more stringent alpha may be required, depending on the panel size.
3. As noted above, the handling and reporting of CHIP may affect the FPR of an assay. The assay should correct for CHIP to ensure that it has an adequate FPR to support clinical use.

2.5. DATA PRESENTATION FORMAT

In most NGS applications, LoB estimates should be set as zero for blank samples and then blank samples may be tested to confirm that no false positives are observed using a LoB of zero. However, developers should report the false positive results at the LoB cutoff observed on a per variant basis, as described above, on a per sample basis. A false positive rate for both mutant-negative samples from individuals with cancer as well as normal donor samples included in this study should be calculated. Assay developers should also be prepared to provide a list of specific "problem" variants in the panel that consistently result in false positive detection.

See also the Appendix A of this document for a worked example of a LoB study.

3. PROTOCOLS FOR CONTRIVED SAMPLE FUNCTIONAL CHARACTERIZATION STUDIES

3.1. INTRODUCTION

Although it is recommended to use clinical samples from the intended use population in analytical validation studies, developers may need to supplement these samples with non-clinical or contrived samples in cases of rare variants or if adequate sample volumes are difficult to obtain. When using samples other than clinical samples during analytical validation, assay developers should plan to provide appropriate justification for their use and to perform an appropriately designed Contrived Sample Functional Characterization (CSFC) study to establish that the clinical and contrived samples are functionally

comparable across different dilution levels around and below the limit of detection (LoD). Failure to do so may lead to erroneous and misleading patient results caused by matrix effects that result from differences in the way the contrived material was formulated, processed, handled, or preserved compared to actual patient samples. It is recommended that assay developers complete the CSFC study before starting any other validation studies. Furthermore, assay developers that seek regulatory approval should consider submitting the CSFC study results to the FDA for review prior to starting the other validation studies.

Contrived samples are often used to supplement clinical samples in cfDNA validation due to the low levels of cfDNA present in the circulation (for example, as low as 1.8-44ng/mL of plasma in normal individuals) [36]. In addition, the rarity of certain variants in patient populations and the need to validate the assay at specific defined MAF levels (for example, ±30% outside of measuring range) may also require the use of contrived samples. The measuring range is the range of values of measurands for which the error of the assay is intended to fall within a specified limit (EP06: A, 2003) [20].

However, although contrived samples that demonstrate equivalence through a CSFC study are acceptable to supplement clinical samples or to represent some rare variants, they are not intended to replace clinical samples. Variants with clinical significance, especially variants with CDx indications, should be adequately, analytically validated with clinical samples from intended use patient population. As noted in the protocols that follow, key studies such as LoD confirmation, accuracy, precision, and some stability studies should be completed using clinical samples from the intended use population.

Based upon the current understanding in the fields of liquid biopsy testing and analytical validation test protocols, three sample types are recommended for use in AV studies depending on the claim and type of study. The sample types are:

1) Clinical specimens – cf/ctDNA derived from plasma specimens obtained from the target population.
2) Contrived samples using cfDNA from cell culture media– Extracted cfDNA from cell line media that has been spiked into extracted cfDNA from the plasma of mutation-negative individuals with cancer or from risk-matched healthy donors Ideally, plasma should not be pooled across cancer types; if it is pooled, then developers should demonstrate that it does not impact the results or conclusions.(See also Section III: Standard Method 1). This method is the preferred contrived sample method to supplement clinical specimens as extracted cfDNA from cell line media already approximates natural cfDNA matrix and fragment size distribution as described in Bronkhorst et al. 2016 [41].
3) Contrived DNA samples using fragmented cell line genomic DNA – Fragmented cell line DNA that has been spiked into plasma from either mutation-negative individuals with cancer or risk-matched normal donors. The spiked plasma

should then be extracted to obtain contrived cfDNA, as described in Section III: Standard Method 2.

In addition, a number of commercially available contrived cfDNA reference materials are available. These materials may be produced through different methods than those described above. An example method is provided in Section III: Standard Method 3.

Note 1: Assay developers who produce contrived cfDNA samples should plan to demonstrate that the resulting sample is comparable to the cfDNA profile generated from cfDNA extracted from clinical specimens from the device intended population. One way to do this is to compare Bioanalyzer (or equivalent) fragment size profiles. The Bioanalyzer DNA profiles should be captured both before spiking and after extraction to show that the DNA profiles matches that of the clinical samples. In addition, ctDNA yield should also be compared between the clinical and contrived samples.

Note 2: The contrived samples prepared for use in AV studies should be not be the same samples used for assay development purposes. Unique samples must be used for each activity; however, in some cases, the same cell line or derivative used to produce the samples may be the same.

Note 3: In all cases, contrived samples should be prepared such that they closely simulate the expected DNA yield and concentration consistent with patient samples. Although developers may need to prepare contrived samples at very low MAF levels to validate the performance of the assay, it is to be noted that contrived samples may not be reproducibility prepared at MAFs <0.1% due to sample errors introduced at those low levels.

Contrived sample functional characterization studies are only required when using sample types 2 and 3 described above, in any AV protocols in this guidance document. In addition, any contrived sample functional characterization study should be done on all variant classes covered by the assay's intended used (for example, SNV, indels, fusions, CNV).

An example "contrived sample functional characterization study" design is described below; however, minimal sample size should be determined for each assay based on its performance goal. As noted below, these studies may be combined with the LoD and/or linearity studies, as appropriate.

3.2. Experimental PROTOCOLS (FOR VARIANTS WITH Qualitative CLAIMS)

1. For variants with qualitative claims, assay developers should demonstrate the functional equivalence between the two sample types across different dilution levels, especially at the lowest dilution levels of the paired clinical and contrived sample types.

BloodPAC
info@bloodpac.org | © 2017 BloodPAC
BloodPAC is a consortium managed by the Center for Computational Science Research, Inc., which is an Illinois based not-for-profit corporation

2. Clinical and contrived samples with the same variants at the same concentration as defined via an orthogonal method should be evaluated. The top-level sample/dilution (clinical and contrived, at least 1 starting sample each) should be paired in level based on quantification using an orthogonal method. Note: If the assumption of the same expected quantified value for clinical samples and contrived samples at each dilution level is not met, then manufacturers will need to test replicates at each dilution level by an orthogonal method for both clinical samples and contrived samples, as well.
3. For qualitative variants, serial dilutions should be prepared such that the clinical and contrived samples are tested at sufficient dilution levels at and below LoD (e.g., C95, C75, C50, C25, C5), per CLSI EP-12-A2 [18], in which Cx represents the concentration at which x number of positive calls out of 100 replicates are observed.
4. Testing should include at least 5 levels between the established LoD and LoB. The assay cutoff should be included in the dilutions if that level is below LoD.
5. Test at least 20 replicates per level.
6. For variants with a qualitative claim, it may be useful to combine this study with the LoD study.
7. Although the minimal requirement is 1 clinical sample, it may be useful to evaluate more than 1 sample to account for potential differences in sample performance.

3.3. Statistical Analysis (for Variants with Qualitative Claims)

For each mutation type, (1) plot the true variant frequency determined by the orthogonal method (x) vs. % positive call (y) for the clinical and contrived samples; (2) fit logit or probit regression model for clinical sample and contrived sample, respectively, Y=dichotomized device output, x=true variant frequency. To establish the sample comparability, the appropriate hypothesis testing is to evaluate the difference of the coefficients between clinical samples and contrived samples as follows, for example:

Assume the fitted regression coefficients for clinical sample (β0cs, β1cs) and contrived samples (β0con, β1con) are equivalent within pre-defined margins:

H0: $|\beta0con - \beta0cs)| \geq \gamma$ or $|\beta1con - \beta1cs)| \geq \gamma$

Ha: $|\beta0con - \beta0cs)| < \gamma$ and $|\beta1con - \beta1cs)| < \gamma$

Because it may be difficult to establish a clinically meaningful equivalence margin, it may be sufficient to describe the difference of the coefficients comparing contrived samples to clinical samples. In addition to providing the estimates of the probit/logistic regression coefficients (i.e., intercept and slope) and the corresponding standard errors, calculate the difference of the coefficients and the corresponding two-sided 95% confidence intervals, comparing contrived samples to clinical samples. The estimates of C25, C50, C75 and C95 as well as the corresponding two-sided 95% confidence intervals (CIs) should also be

BloodPAC
info@bloodpac.org | © 2017 BloodPAC
BloodPAC is a consortium managed by the Center for Computational Science Research, Inc., which is an Illinois based not-for-profit corporation

provided. The differences of C5, C25, C50, C75 and C95 and the corresponding CIs comparing contrived samples to clinical samples should also be included. Per above, please note that all analyses should be performed for each variant class covered by the assay's intended use (for example, SNVs, indels, fusions, CNV).

3.4. Experimental Protocols (FOR VARIANTS WITH QUANTITATIVE CLAIMS)

1. For variants with quantitative claims (e.g. monitoring claim or report MAF), assay developers should demonstrate the functional equivalence between the two sample types across the whole reportable range for that variant.
2. Clinical and contrived samples with the same variants at the same concentration should be evaluated. The top-level sample/dilution (clinical and contrived) should be paired in level and quantified using an orthogonal method.
3. The paired top-level clinical/contrived samples should then be diluted down to many levels to hit the high and low end of the range.
4. For variants with a quantitative claim, it may be useful to combine this study with the linearity study.
5. Although the minimal requirement is 1 clinical sample, it may be useful to evaluate more than 1 sample to account for potential differences in sample performance.

3.5. Statistical Analysis (FOR VARIANTS WITH QUANTITATIVE CLAIMS)

For variants with quantitative claims, the method comparison approach can be used to estimate the agreement of quantitative values (such as MAF) between the two sample types (contrived and clinical) for that variant. For each quantitative variant, plot the measured values observed using the clinical samples (x) vs. the measured values observed using the contrived samples (y) (Figure 1). Specifically, an appropriate regression model can be used to estimate the slope $\beta 1$ intercept $\beta 0$ and their associated confidence intervals. Assay developers can also calculate the predicted biases and their 95% confidence intervals from the regression analysis at medical decision points (e.g. for monitoring claim). In addition, Bland-Altman (difference) plots are also recommended to be used to assess any differences between two sample types.

As a supplementary analysis (not pictured), it is also recommended to plot the measured values observed using the new assay (y) versus the expected values calculated according to the dilution series (x) to assess the scatter around the line to assess the comparability of the variability between the contrived and the clinical samples. The scatter can be visualized by constructing the following plots:

1. For the dilution series of clinical samples, plot x=the expected values calculated according to the dilution series, y=measurement by new assay. Include fitted regression line.

BloodPAC
info@bloodpac.org | © 2017 BloodPAC
BloodPAC is a consortium managed by the Center for Computational Science Research, Inc., which is an Illinois based not-for-profit corporation

2. Construct a plot the same as in #1 except for contrived samples covering a similar range of reference values.
3. Superimpose plots 1 and 2 to view both the comparability of the fitted regression lines and the variability of points around the fitted lines.

Note: If the assumption of the same expected quantified value for clinical samples and contrived samples at each dilution level is not met, then manufacturers will need to test replicates at each dilution level by an orthogonal method for both clinical samples and contrived samples. In this scenario, this supplementary analysis will become the primary analysis. Manufacturers should instead plot the measured values observed using the new assay (y) versus the measured values observed using the orthogonal assay (x) for this analysis.

3.6. Summary Statement

When the contrived samples behave functionally equivalent to the clinical sample, based on the recommendations described above, the contrived samples may be used for some analytical validation studies, as described in the protocols below. However, the same contrived samples that have been used during assay development should not then be used in the validation of the same assay. There may be cases, however, for some variants, in which the same cell line or derivative used to produce the contrived sample may be the same in both cases. Contrived samples, although shown to be equivalent to clinical samples through CSFC, cannot replace the need for clinical samples, as noted in some key protocols below (LoD, accuracy, and precision).

3.7. DATA PRESENTATION FORMAT: CONTRIVED SAMPLE FUNCTIONAL CHARACTERIZATION STUDY

Table 1 Data Presentation Format (Qualitative Claims)

	β0 (95% CI)	B1 (95% CI)	LoD (95% CI)	C75 (95% CI)	C50 (95% CI)	C25 (95% CI)	C5 (95% CI)
Contrived							
Clinical							
Difference (Contrived-Clinical)							

The above table should list coefficients, LoD, C75, C50, C25, C5 and 95% CIs for clinical samples, contrived samples, and the difference between the two.

BloodPAC
info@bloodpac.org | © 2017 BloodPAC
BloodPAC is a consortium managed by the Center for Computational Science Research, Inc., which is an Illinois based not-for-profit corporation

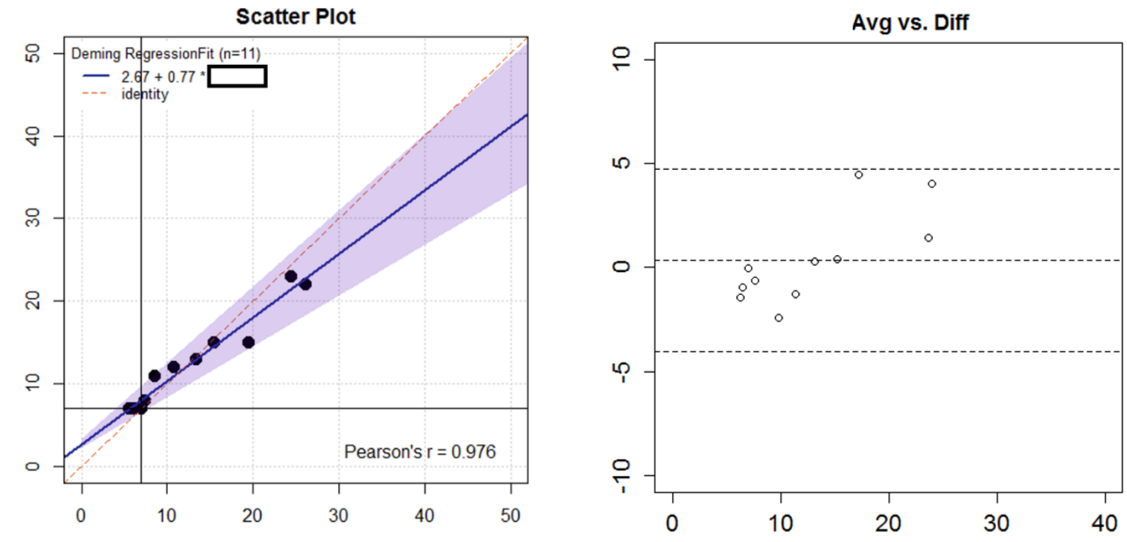

Figure 1 Data Presentation Format (Quantitative Claims) Left: Deming regression model with identity line, fitted line, model coefficients, and raw data points. X-axis denotes observed values from clinical samples and Y-axis denotes observed values for contrived samples. In addition, 95% confidence intervals for the coefficients and point estimates and 95% confidence intervals at medical decision points should be included. Right: Bland-Altman plot of the difference (X-Y) on the vertical axis versus the average (X+Y)/2 on the horizontal axis.

Note: Deming regression should be used with caution, as it requires equal error variance for the two assays or at least a known ratio of variances.

4. Protocols for Evaluation of Limit of Detection (LoD)

4.1. Introduction

LoD is defined as the lower limit of detection. The LoD is the lowest allelic frequency of SNV, CNV, indels, MNV, or structural variants such as fusions at the DNA level, that can be detected at least 95% of the time at the nominal DNA input level specified by the assay's instructions for use (CLSI EP17-A2) [16]. Due to the low abundance of cfDNA in plasma, contrived cfDNA samples or sample blends prepared across various levels may be required to attain adequate samples for the LoD determination. Contrived cfDNA samples should be prepared for this study as described in Standard Method 1 and Standard Method 2 or characterized reference materials should be selected to simulate the apoptotic fragmentation footprint that is characteristic of cfDNA. Any contrived samples used should be prepared in a method that is size-representative (target of 170 bp when sample is

plasma) of cfDNA (demonstrated by testing on a Bioanalyzer or equivalent) and should demonstrate equivalence to plasma-derived cfDNA from patient samples as determined in the CSFC study above.

In addition, due to limiting sample volumes and due to the extended workflow and increased cost of NGS-based assays compared to other detection assays, the traditional probit approach of testing 20 replicates per level may not be manageable for all developers. For those cases, a minimal replicate number of 10 per level may be proposed. Please note, though, that 20 replicates per level (i.e., 10 replicates per lot x 2 lots) is recommended; if 10 replicates are used, it will be at the assay developer or manufacturer's risk.

Contrived samples may be used to determine the LoD; however, the confirmation of the LoD must be performed using true patient samples or pools of patient samples. Note that developers should not pool across cancer types. Pools should be made within each cancer type and certain variant types. Accountability of pooling should be tracked as it helps calculate the final allelic frequencies due to pooling/diluting of targets. In cases of sample limitations, it may be helpful to pool the results instead of pooling samples. For variants with qualitative claims, it may be useful to combine the LoD study with the CSFC study described above.

A note on reporting LoD: LoD should be reported in the same unit(s) as clinical cutoff (e.g., MAF for SNVs, MNVs, indels; copy number for CNVs, fusion reads for rearrangements/fusions, etc.). Because MAF is a ratio, it is recommended to include another quantitative measure in addition to MAF for reporting the LoD of SNV's, MNV's and indels. Therefore, as described in "Data Presentation Format" below, assay developers should be prepared to report LoD using 1) MAF, or other unit(s) used for the clinical cutoff, 2) unique molecules, and 3) nanogram input at which the LoD was observed. In addition, if assay developers plan to label their device as detecting true positives below LoD, then appropriate clinical validation data should be provided. Variants detected in this range should be included in the analytical accuracy study described below.

4.2. Experimental Protocols: Preparation of Contrived Samples

1. DNA dilution panels containing variants at discrete allele frequencies, which are quantified using an orthogonal method such as another NGS assay employing UMIs or a validated dPCR assay (See Section III: Standard Method 1 or Section III: Standard Method 2).
2. Size/efficiency representative of cfDNA
3. Diluted into size representative normal DNA background demonstrated to be wild type at each locus of interest using an orthogonal method
4. Specimen blend panels will be constructed and studied for the predominant variants representing the assay's intended use. A representative testing strategy is acceptable for SNVs and indels; however, it should not be used for the other

structural variants (e.g., fusions and copy number variations) in the panel. In addition, for CDx variants, a representative approach will not be applicable. Additional variants which may be detected across the interrogated regions, such as heterozygous germline polymorphisms which may be present at <50% MAF after blending of DNAs, may also be used to establish LoD, as appropriate.

4.3. Experimental Protocols: Evaluation of Limit of Detection (LoD)

1. An appropriate number of low-level positive samples/specimen blend panels. The number of samples may vary, but should represent all of the variant types to be detected and the assay claims.
2. At least 5 allele frequency dilution levels per sample/panel
3. N replicates of testing for each level
4. N lots of reagents/assay
5. Each panel should test 1-2 representative variants (driver specific) of each class, as described in 11.2 above
6. LoD should be assessed at most challenging assay condition— typically, this is at the lowest cfDNA input

4.4. Minimal Experimental Design

1. At least 2 independent reagent lots. Note whether the assay/device performance is the same between lots; data from two lots may be combined for analysis.
2. An appropriate number of low-level positive samples/specimen blend panels per reagent lot. The number of samples will be chosen, as needed, to represent all variant types to be detected.
3. At least 2 replicates for each independent sample run within a reagent lot
4. At least 60 tests per reagent lot total across all 5 allele frequency dilution levels

4.5. Minimal Experimental Design: probit Regression Model

1. 2 reagent lots
2. An appropriate number of low level positive samples/specimen blend panels, as needed to represent all of the variant types to be detected and the assay claims
3. At least 5 dilutions per positive samples around the targeted LoD. It is desired to have only 1 dilution level in the extreme levels (with hit rates <0.10, >0.90).
4. Test 20 replicates per level (10 reps/lot x 2 lots) across 5 dilution levels (10 x 2 x 5) for a total of 100 measurements

Note: Probit may be over fitted if only 1 or 2 of the positive sample dilutions prepared above yields a hit rate between 0.10-0.90. In this case and in general, it is always helpful to empirically test the lowest level at which 95% hit rate is observed.

BloodPAC
info@bloodpac.org | © 2017 BloodPAC
BloodPAC is a consortium managed by the Center for Computational Science Research, Inc., which is an Illinois based not-for-profit corporation

4.6. Statistical Analysis using the Parametric and Non-Parametric Models for Variants with Quantitative Claims

For variants with quantitative claims, parametric analysis could be used to calculate the LoD estimates when the variability of the measurement results is relatively consistent across the low-level samples. Non-parametric method and probability models could also be utilized for the LoD calculation for quantitative variants to compensate for the instability of the reactions or wide measurand range across the low-level samples. The calculation based on parametric method, non-parametric method, and precision profile method are detailed in CLSI EP17-A2 [16]. This document will focus on the probit approach to evaluate the LoD.

Probit model LoD calculation for variants with Qualitative Claims

Analyze the data from all starting samples for each reagent lot independently

1. Calculate the hit rate Hi for each dilution as Hi=N(pos)i/N(tot)i; where N(pos)i is the number of replicates reported as positive for presence of the measuring and N(tot)i is the total number of replicates processed for the ith dilution.
2. Input the hit rates as y-axis and their corresponding measurand concentrations as x-axis to perform a probit fit. Typically, log10 concentration on the x-axis improves the probit fit.
3. Evaluate the model with a statistical goodness of fit test. If the goodness of fit is not acceptable, it may be necessary to improve the goodness of fit by testing additional dilutions and/or replicates at the existing dilution level as appropriate.
4. If the model fit is deemed acceptable, the measurand concentration corresponding to the desired β error risk (β=0.05 for a hit rate of 0.95) is the LoD for the reagent lot.
5. Repeat above steps to obtain LoD results for all reagent lots.
6. The maximum LoD value from all reagent lots is reported as the LoD estimate for the assay.

4.7. Data Presentation Format

BloodPAC
info@bloodpac.org | © 2017 BloodPAC
BloodPAC is a consortium managed by the Center for Computational Science Research, Inc., which is an Illinois based not-for-profit corporation
.

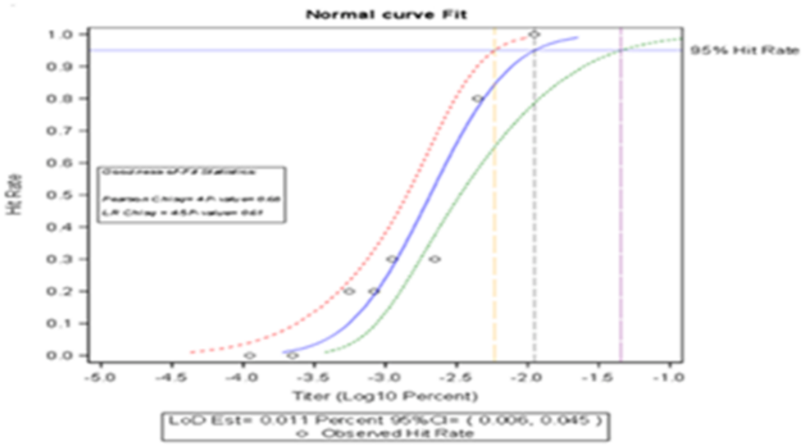

Figure 2 LoD Estimate of Probit Model Analysis

Table 2 Limit of Detection Example Hit Rate Data

[illegible]

Target	DNA Type	Plasma Source	5x LoD (Round 1)	1x LoD (Round 1)	1x LoD (Round 2)	0.75x LoD (Round 2)	0.5x LoD (Round 1)	0.2x LoD (Round 2)	0.1x LoD (Round 2)	0.03x LoD (Round 2)
Ex. 19del	Intact	NSCLC	100% (83.2-100)	100% (83.2-100)	95% (75.1-99.9)	100% (83.2-100)	100% (83.2-100)	95% (75.1-99.9)	100% (83.2-100)	75% (50.9-91.3)
		HD	100% (83.2-100)	95% (75.1-99.9)	100% (83.2-100)	100% (83.2-100)	100% (83.2-100)	100% (83.2-100)	90% (68.3-98.8)	80% (56.3-94.3)
	Sheared	NSCLC	100% (83.2-100)	100% (83.2-100)	100% (83.2-100)	100% (83.2-100)	100% (83.2-100)	100% (83.2-100)	95% (75.1-99.9)	65% (40.8-84.6)
		HD	100% (83.2-100)	95% (75.1-99.9)	100% (83.2-100)	100% (83.2-100)	100% (83.2-100)	95% (75.1-99.9)	80% (56.3-94.3)	70% (45.7-88.1)
L858R	Intact	NSCLC	100% (83.2-100)	100% (83.2-100)	100% (83.2-100)	95% (75.1-99.9)	95% (75.1-99.9)	85% (62.1-96.8)	75% (50.9-91.3)	25% (8.7-49.1)
		HD	100% (83.2-100)	80% (56.3-94.3)	100% (83.2-100)	100% (83.2-100)	75% (50.9-91.3)	95% (75.1-99.9)	45% (23.1-68.5)	0% (0-16.8)
	Sheared	NSCLC	100% (83.2-100)	95% (75.1-99.9)	100% (83.2-100)	100% (83.2-100)	85% (62.1-96.8)	90% (68.3-98.8)	80% (56.3-94.3)	20% (5.7-43.7)
		HD	95% (75.1-99.9)	85% (62.1-96.8)	100% (83.2-100)	100% (83.2-100)	95% (75.1-99.9)	100% (83.2-100)	70% (45.7-88.1)	5% (0.1-24.9)

4.8. Experimental Protocols: Confirmation of LoD

Note that the minimal experiment design presented above is for the establishment of LoD. If this was done using contrived samples, then the following confirmation study should be

completed after the LoD has been established using contrived samples only. If the establishment study was done as described above using clinical samples in place of non-clinical samples, then the sponsor does not need to conduct the LoD confirmation study outlined below.

1. At least 1 positive patient sample prepared from true patient samples (clinical samples) or pools of patients diluted to the calculated LoD using an orthogonal method, such as another NGS assay employing UMIs or a validated dPCR assay.
2. 20 replicates of testing at the established level
3. 2 reagent lots (10 replicates/lot)
4. Each panel should test 1-2 representative variants (driver specific) of each class

4.9. Statistical Analysis: Confirmation of LoD

Determine the hit rate for each variant.

4.10. Data Presentation Format: Confirmation of LoD

Present the hit rate at the LoD, with confidence interval reported. LoD should be reported in the same unit(s) as clinical cutoff.

5. Protocols for Evaluation of Analytical Accuracy

5.1. Introduction

Analytical accuracy can be assessed by method comparison (comparing the results from the test method to an orthogonal method, on a given set of samples) or with known reference standards. The protocols described here include both approaches and leverage many aspects of the New York State Next Generation Sequencing guidelines (Jan 2018) for somatic variant detection and other guidance [19, 32].

Due to the high sensitivity, breadth of genomic coverage, and range of variants detected by NGS-based cfDNA assays, it may be challenging to select an appropriate orthogonal method against which to validate. In particular, the currently approved PCR-based IVD for mutation detection from cfDNA differs in the sensitivity capabilities of NGS and does not represent the range of targets and variant types included in the emerging cfDNA NGS panels. Likewise, matched FFPE tissue testing may also not always be concordant to plasma-based NGS cfDNA assay results due to difference in assay sensitivities, tumor heterogeneity, and biological and treatment factors that influence how the patient's disease state is represented in the circulation. New assay developers or test manufacturers should select an orthogonal method that does not have the same limitations as the test being validated. Therefore, the orthogonal method should ideally be based on a technology that

BloodPAC
info@bloodpac.org | © 2017 BloodPAC
BloodPAC is a consortium managed by the Center for Computational Science Research, Inc., which is an Illinois based not-for-profit corporation

does not have the same limitations as the test being validated; if not, the study will reproduce the same erroneous results/error profiles and will not be a true evaluation of accuracy.

Accuracy should be determined for Level 1 companion diagnostic (CDx), Level 2 and Level 3 variants, and for the panel-wide, SNVs and indels based on a representative approach (1). All CDx variants should be included in the accuracy study. Accuracy should be determined for all genes with copy number alterations (CNAs) and fusions on the panel, unless the sponsor has data to demonstrate that the performance of these variant types are optimum across different genomic regions interrogated by their device.

True patient samples or pools of patient samples should be prioritized for use in this accuracy study, as described below.

Priority 1: Clinical trial specimens
Priority 2: Procured clinical specimens.

Experimental Protocols

Note, that the sample numbers stated below are a recommendation only. The adequate number of variants positive and negative samples to be used to assess accuracy will depend on a number of factors, including the assay's intended use, performance goals, size of the panel, clinically relevant/significant genes in the panel, CDx claims, tumor profiling claims, and pan-tumor claims. Assay developers who seek regulatory approval should discuss the sample size with the FDA.

1. Variant negative samples: Sequence a minimum of approximately 100 samples known to be negative for cancer-relevant mutations across all regions interrogated by the assay. Error rate should be <2%. If an unexpected variant can be verified by an orthogonal method (e.g. dPCR), it can be treated as a true positive.
2. Variant positive samples: 10 to 20 positive samples for each type of intended Level 1 significance variants (variants included in the assay's IVD claim) must be sequenced and confirmed. Performance may be assessed across a collection of clinically significant variants of a given variant type in this study. As mentioned earlier, the total number of samples for the accuracy study should be calculated based on the assay's intended use, performance goals, size of the panel, clinically relevant/significant genes in the panel, CDx claims, tumor profiling claims, and pan-tumor claims. The existing approvals for FoundationOneCDx test may be used as a recent guidance to this approach [39]. However, new assay developers are encouraged to observe the most current regulatory approvals on this topic at the time of beginning any new validation study.
3. For variants with very low prevalence (like rare fusions), the accuracy study should attempt to accommodate at least 5 unique specimens. The Summary of Safety and

Effectiveness Data of the Thermo Fisher Oncomine Dx Target Test PMA submission may be used as a current guidance to this approach [40].
Note that variant positive and negative samples in 1 and 2 above may serve "double-duty" across a panel to reduce the total number of samples needed in this study. For example, BRAF positive samples may serve as negative samples when the EGFR gene is assessed. In addition, samples with more than one variant may be used to cover multiple gene targets within the panel. However, new assay developers should include some known WT samples (from patients in the same clinical population as the positives that are mutation-negative for the hotspot of interest) in this study.

4. For cfDNA samples, confirm detected variant(s) with an orthogonal method (dPCR or an NGS platform in another lab developed independently of the test being validated).

5.2. Statistical Analysis for Variants with Quantitative Claims

For variants with quantitative indications, accuracy should be assessed by comparing the quantitative analyte (e.g., MAF) measured by both the new cfDNA assay under development and by an appropriate orthogonal (reference) method, which has a similar level of sensitivity (at least 80-85% overlap) as the new method. The method comparison approach may be used to estimate the agreement of quantitative values between two assays for each variant:

- An appropriate regression model may be used to estimate the slope β1 intercept β0 and their associated confidence intervals. The predicted biases and their 95% confidence intervals from the regression analysis at medical decision points (e.g., for a monitoring claim for a specific variant) can then be calculated
- Any differences in variance about the regression line should also be captured and reported
- In addition, the use of Bland-Altman (difference) plots is also recommended to be used to assess any differences between the new assay and the orthogonal method

5.3. Statistical Analysis for Variants with Qualitative Claims

The analytical accuracy is reported as positive percent agreement (PPA), negative percent agreement (NPA), and overall percent agreement (OPA) in reference to a reference detection method and their corresponding confidence intervals [1.a.i.42 42]. The total number positive by the orthogonal method should be set as the denominator to calculate PPA and the total number negative by the orthogonal method should be set as the denominator to calculate the NPA.

Due to the large number of mutations that are reported by NGS panels, PPA and NPA will be calculated in the following ways:

BloodPAC
info@bloodpac.org | © 2017 BloodPAC
BloodPAC is a consortium managed by the Center for Computational Science Research, Inc., which is an Illinois based not-for-profit corporation

- PPA/NPA will be calculated and reported for all variants and separately for each variant type detected by the assay.
- PPA/NPA will be calculated and reported individually for each of the Level 1 significance variants of the assay's intended use.
- PPA/NPA should be determined at the sample, variant, and bin- levels for Level 1, 2, and 3 significance variants, and for the panel-wide analysis. In this example, "bin" may be defined as variant class.

Please note that the ways to calculate PPA/NPA and PPV/NPV may vary depending on how samples are selected. If samples are selected using orthogonal method, then PPA/NPA can be directly calculated from the concordance table between test device and orthogonal method, but PPV/NPV needs to be calculated by adjusting for the proportion of variant positive samples by orthogonal method. In contrast, if samples are selected using test device, then PPV/NPV can be calculated directly from the concordance table between test device and orthogonal method, but PPA/NPA needs to be calculated by adjusting for the proportion of variant positive samples by test device.

Note: Caution should be taken in reporting PPV and NPV. These terms only have value in the intended use population and may not be interpretable in a different population.

5.4. Data Presentation Format

Table 3 PPA Results for Qualitative Measurements

PPA measure	Excluding no calls		Including no calls	
	Percent agreement	95% CI	Percent agreement	95% CI
Variant	98.5% (195/198)	(95.6%, 99.7%)	98.5% (195/198)	(95.6%, 99.7%)
Bin	97.2% (176/181)	(93.7%, 99.1%)	97.2% (176/181)	(93.7%, 99.1%)
Sample	96.9% (158/163)	(93.0%, 99.0%)	96.9% (158/163)	(93.0%, 99.0%)

In this example, "bin" is defined as a class of variants. For example, simple SNVs, complex SNVs (SNVs in di-/tri-nucleotide repeat regions and multi-nucleotide variant, deletions and fusions are classes of different bins.

6. Protocols for Evaluation of Linearity

BloodPAC
info@bloodpac.org | © 2017 BloodPAC
BloodPAC is a consortium managed by the Center for Computational Science Research, Inc., which is an Illinois based not-for-profit corporation
.

6.1. Introduction

In the case of monitoring the level of a biomarker, it is necessary to illustrate that the analytical method is linear. This range may be more restricted than the given measuring range for an assay. That is, there exists a mathematically verified straight-line relationship between the observed values and the true concentrations or activities of the analyte (EP06-A: 2003) [20]. For a monitoring assay, there must be a linear relationship between the allele fraction reported by the laboratory and the true allele fraction in the blood. For example, if the true allele fraction of an informative biomarker were to double in the blood, this should be reflected in a doubling of reported allele fraction. For linearity studies, the ideal sample matrix is a patient's specimen pool with an analyte concentration near the expected upper reportable limit that is diluted with another patient's sample pool having an analyte concentration at the expected or tested lower limit (EP06-A:2003) [20]. Developers should determine what clinically relevant levels should be selected based on the targeted patient population based on the assay's intended use. However, given the difficulties of obtaining sufficient amounts of blood from patients with a very high level of biomarker to create such pools, contrived DNA samples should be used, as appropriate, and should be size and efficiency representative of true patient samples (use a Bioanalyzer to demonstrate the fragmented size profile is comparable to that of cfDNA from plasma), and should be demonstrated as functionally equivalent as described in Section 10 above. An example study design for linearity is described below; however, minimal sample size should be determined for each assay based on its performance goal. Note that this study design may be combined with the "Protocol for Contrived Sample Functional Characterization Studies" described above for variants with quantitative claims.

6.2. Experimental Protocols: Preparation of Contrived DNA

1. Sample dilution panels constructed from orthogonally validated control DNA
2. Size/efficiency representative of cfDNA
3. Quantified against an orthogonal method
4. Diluted into extracted WT cfDNA (derived from normal donors)
5. Specimen blend panels will be constructed and studied for the predominant variants listed in the assay's Intended Use.

6.3. Experimental Protocols: Evaluation of Linearity

1. At least 11 levels that cover the entire measuring range of the assay. It is recommended to test over a range that is 20 to 30% wider than the anticipated measuring range (for example, 0.1%-100%AF), and then plan to eliminate nonlinear points to establish the widest possible range of acceptable linear response
2. 1 instrument
3. 1 reagent lot

4. At least 4 replicates at each level; however, a larger number of replicates may be needed depending on the imprecision of the assay

6.4. Statistical Analysis

The Polynomial evaluation assumes that the data points fall perfectly on a line or curve in the absence of random error. The method consists of two parts. The first part examines whether a nonlinear polynomial fits the data better than a linear one. The second part, performed in cases when a nonlinear polynomial fits the data better than a linear one, assesses whether the difference between the best-fitting nonlinear and linear polynomial is less than the amount of allowable bias (predefined) for the method.

6.5. Data Presentation Format

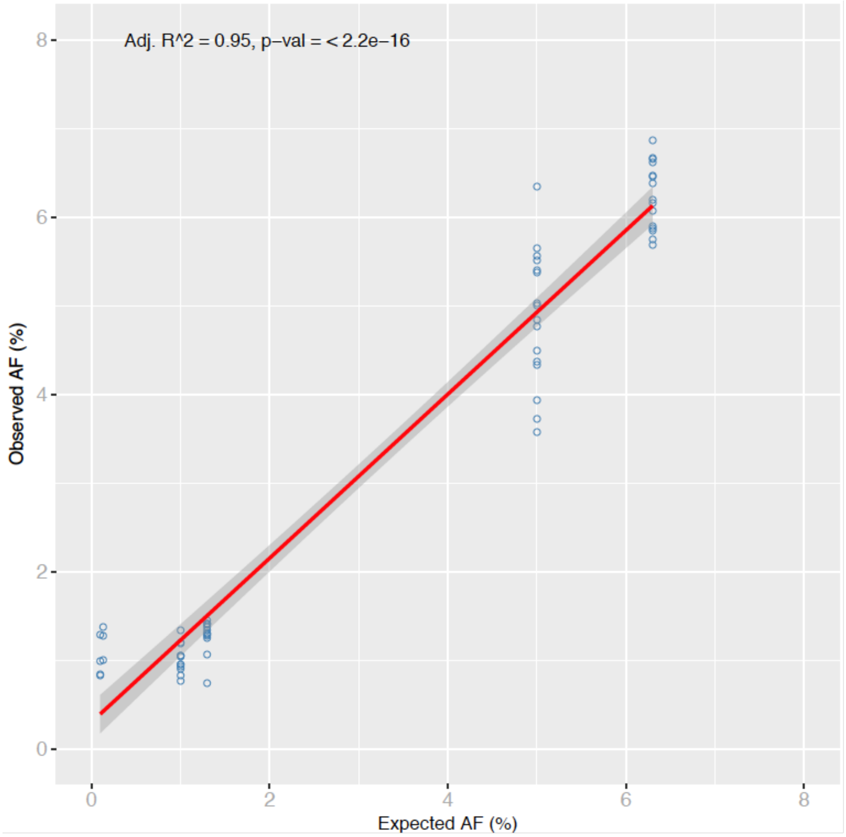

Figure 3 Polynomial Regression Analysis in the Linear Range

Table 4 Polynomial Regression Analysis in the Linear Range. Note: MAF range shown is an example only; a different (lower) MAF range may be more relevant to cfDNA.

Variant	Range of Target AFs	Quadratic model *p*-value: Quadratic coef.	Cubic model *p*-values: Quadratic coef.	Cubic model *p*-values: Cubic coef.	Linear range
EGFR3 S249C	2-15%	0.119	0.020	1.000	2 - 15%
FGFR3 S249C	4-15%	0.094	0.908	0.961	4 - 15%

7. Protocols for Evaluation of Limit of Quantitation (LoQ)

7.1. Introduction

LoQ does not apply to qualitative assays. This study will be required of developers who seek a quantitative claim. The LoQ will be applicable to the specific variants in a panel with a quantitative claim and not to the entire panel.

For quantitative assays, it may not be necessary to determine the LoQ if the total error can be determined and reported for all results at low allele fractions, allowing the user to interpret whether or not it is suitable for use. LoQ represents the lowest allele fraction that can be quantitatively determined with stated accuracy (as total error or as independent requirements for bias and precision) (CLSI EP17-A2) [16]. The LoQ could be equal to or greater, but never lower, than the LoD. As stated above, developers who seek monitoring claims will need to establish the LoQ for their assay to demonstrate the assay's ability to distinguish biological changes in the level of ctDNA in circulation from technical variation and measure the ctDNA level with sufficient accuracy.

An example study design is described below; however, minimal sample size should be determined for each assay based on its performance goal. Individual sponsors may choose to incorporate the evaluation of LoQ into another protocol, such as Repeatability, as appropriate.

7.2. Minimal Experimental Design: preparation of Contrived samples

1. Panels constructed from orthogonally validated control DNA
2. Size/efficiency representative of cfDNA
3. Quantified against an orthogonal method
4. Select a target concentration (expected LOQ) and prepare 4 dilutions at this level, by diluting into extracted WT cfDNA.
5. Specimen blend panels will be constructed and studied for the predominant variants listed in the assay's Intended Use.

BloodPAC
info@bloodpac.org | © 2017 BloodPAC
BloodPAC is a consortium managed by the Center for Computational Science Research, Inc., which is an Illinois based not-for-profit corporation
.

7.3. Minimal Experimental Design: Evaluation of LoQ

1. At least 2 separately manufactured reagent lots
2. At least 3 days
3. At least Four independent low AF contrived samples pools (diluted at target LoQ); A suggested study design is described below; however, minimal sample size must be determined based on the LoQ acceptance criterion (which targets an acceptable total error). LoQ acceptance criteria need to be determined for each assay based on its performance.
4. At least 36 total low AF contrived samples pool replicates per reagent lot (across all samples, instrument systems, and days)

7.4. Statistical Analysis

The LoQ is a performance attribute applicable only to quantitative measurement procedures. Given the flexibility of the LoQ definition, LoQ is reported with the underlying accuracy goals for both bias and precision. In a classical approach using two reagent lots, the sample with the lowest concentration that met the accuracy specifications is taken as the LoQ for that lot. The greatest LoQ across all lots or the LoQ from the combined dataset is taken as the LoQ for the measurement procedure. A variant approach may also be appropriate that enables evaluation of the LoQ as part of a LoD evaluation using the precision profile approach.

7.5. Data Presentation Format

N/A

8. Protocols for Evaluation of Repeatability, Precision and Reproducibility

8.1. Introduction

The precision of an analytical procedure expresses the closeness of agreement (degree of scatter) between a series of measurements obtained from multiple sampling of the same homogeneous sample under the prescribed conditions. Precision may be considered at three levels: repeatability, intermediate precision and reproducibility. For the purpose of this study, repeatability and reproducibility will be used to assess intermediate precision.

In this study, to capture the variability in true patient matrices, real patient samples or pools of multiple patient samples should be prioritized for use in this protocol, as described below:

Priority 1: Use patient pools or pooled patient samples diluted in normal donor plasma. Prepare patient pools according to the Standard Method outlined in "***Section III: BloodPAC AV WG Standard Methods***." It is recommended to pool the same variant type from within

info@bloodpac.org | © 2017 BloodPAC
BloodPAC is a consortium managed by the Center for Computational Science Research, Inc., which is an Illinois based not-for-profit corporation

the same donor, if possible, and as stated above, pooling should not be done across cancer types. New assay developers should plan to address how pooling may affect the bioinformatics pipeline for germline removal. When assessing mutations with high prevalence in the intended use population, clinical samples should be prioritized as much as possible.

Priority 2: If insufficient patient samples pool volume is available or if pooling is not feasible to attain the variant/MAF combinations required, then developers may use contrived samples or may supplement patient samples/pools with contrived for some studies, as described below.

However, due to the challenge of the limited plasma volume from each patient and the rarity of some variants in the population, it is acceptable to supplement patient samples/pools with contrived samples that were shown to be functionally comparable to patient samples through the CSFC study. Also, due to the extended workflow of NGS (for example, up to 24 hours per run), in this set of protocols, "Run" will necessarily be confounded by "Day." The protocols have been presented here as separate study designs, but some developers may present combined study designs to assess these factors. Example study designs are described below; however, minimal sample size should be determined for each assay based on its performance goal. In addition, due to the limited samples, the repeatability and precision/reproducibility study may be combined into one study. One possibility could be to include lots, runs, days, and instruments in the factorial design, while confounding operators. If there are multiple confounding factors, then it may be difficult to investigate the cause of a study failure.

8.2. Experimental Protocols: REPRODUCIBILITY

1. Multiple samples representing Level 1, 2, and 3 variants as well as panel-wide variants (See Reference 1 for a description of Level 1/2/3/4 claims).
2. Minimal study design:
   i. *For 1 instrument at 3 sites:* 2 operators/site; 3 manufacturers' lots; at least 2 technical replicates (dilution) per sample per run, tested over the course of 20 days per CLSI EP05 [25]
   ii. *For a single site PMA using 3 devices:* 2 operators; 3 manufacturers' lots; at least 2 technical replicates (dilution) per sample per run, tested over the course of 20 days per CLSI EP05 [25].
   iii. *Alternate minimal design for cases of clinical samples with limited specimen volume: 2 replicates/run, 3 reagent lots, 4 instrument/operator combinations (e.g. 2 instruments/2 operators) with a total of 2*3*4=24 data points per sample per site, to be*

BloodPAC
info@bloodpac.org | © 2017 BloodPAC
BloodPAC is a consortium managed by the Center for Computational Science Research, Inc., which is an Illinois based not-for-profit corporation

conducted over multiple days where days are not required to be factorial since the assay is run over multiple days.

3. Contrived samples may be used, if appropriate, based on performance in the CSFC study, but real patient samples should be prioritized as discussed in the introduction above. Note: When using contrived samples, 36 replicates per sample should be tested in this study.
4. Contrived DNA samples will be combined to cover all Level 1 significance variants (and all types of variants reportable in the assay) at 2 levels of input: Use multiples of LoD to determine the minimum of 2 levels of input to test in the protocol (1 level close to LoD at 1x-1.5x LoD and one level at 2x-3xLoD). In addition to these low-level samples, at least 1 wild type sample should also be included in this study.
5. The Repeatability study may be combined with the Reproducibility study described above.
6. In cases in which contrived samples or clinical samples with no specimen volume limitation are available, the developers should consider adding more data points/levels to the study designs above.

8.3. Experimental Protocols: INTERMEDIATE PRECISION

1. Multiple samples representing Level 1, 2, and 3 variants as well as panel-wide variants (See Reference 1 for a description of Level 1/2/3/4 claims).
2. 1 operator, minimum of three instruments at one site, 10 replicates, contrived DNA samples (standard method for prep), will be combined to cover all Level 1 significance (and all types of variants reportable in the assay), tested over the course of 20 days per CLSI EP05 [25] to assess intermediate precision.
3. The Reproducibility study may be combined with the Repeatability study described above.

8.4. Statistical Analysis for Variants with Quantitative Claims

ANOVA or the like model for random effects or components of variance model (typically with multiple factors) may be utilized to identify the sources of variations. The precision is measured as %CV= (SD/Mean)*100[1] using the Satterthwaite/chi-square approach are typically constructed. In this example, SD represents variance components rather than a direct computing of SD across values. In addition, a laboratory should establish its own precision of a measurement procedure.

[1] Note that 95% CIs of %CVs do not need to be reported in this study; however, developers may opt to capture this information to provide some measure of the uncertainty of the estimated quantity.

8.5. Statistical Analysis for Variants with Qualitative Claims

1. For variants with qualitative claims, agreement analysis based on the assay's binary output (variant detected vs. not detected) should be performed. For example, if a mutation status for each sample tested above can be determined using an orthogonal method, then PPA/NPA can be directly calculated by comparing the new assay's test results to that of the orthogonal method. Otherwise, majority call rate can be calculated in the absence of orthogonal measurement; however, caution should be used with this approach, especially in cases of a slim majority. PPA/NPA should be calculated at different levels such as variant level, bin level (e.g. gene level or variant type) and sample level.
2. Additional analyses such as pairwise agreements may also be needed to assess the agreements between specific factors (e.g. lot, operator, and instrument). The agreement analysis should be done for each variant with clinical claims and clinically relevant variant separately, as well as for the whole panel (e.g. panel-wide analysis).
3. The two-sided 95% confidence intervals for the agreements should also be calculated and reported.

11.5. Data Presentation Format

Table 5 Overall Mean and Standard Deviation Observed

	Allele Frequency		Within-run		Between-system		Between-operator		Between-lot		Total	
Variant	Mean value	N	SD	CV	SD	CV	SD	CV	SD	CV	SD	CV
COSM6223	0.1347	36	0.01166	8.70%	0.0069	5.10%	0	0.00%	0	0.00%	0.01355	10.10%
COSM707	0.1159	36	0.01274	11.00%	0.00482	4.20%	0	0.00%	0.0055	4.70%	0.01483	12.80%
COSM6224	0.0994	35	0.00981	9.90%	0	0.00%	0	0.00%	0.0027	2.70%	0.01097	11.00%
COSM754	0.1316	36	0.01027	7.80%	0.00336	2.60%	0.00373	2.80%	0.0036	2.80%	0.01201	9.10%

9. Protocols for Evaluation of Interfering Substances

9.1. Introduction

The purpose of this protocol is to identify potential interference of the assay's Intended Use by endogenous (e.g., high molecular weight DNA, hemoglobin, albumin, triglycerides) and should include conjugated or unconjugated or bilirubin and cholesterol. In addition, developers should plan to assess exogenous substances, such as buffer components and additives from BCTs to evaluate the effect of short blood draws. Developers should determine which substances to include based on identified potential interferents in the assay's intended patient population and specimen type, including common medications

BloodPAC
info@bloodpac.org | © 2017 BloodPAC
BloodPAC is a consortium managed by the Center for Computational Science Research, Inc., which is an Illinois based not-for-profit corporation

that patients may be taking. An example study design is described below; however, minimal sample size should be determined for each assay based on its performance goal.

9.2. Experimental Protocols: Sources of Interference

Endogenous and Exogenous Interfering substances

1. Refer to Appendix B of CLSI EP07 for suggested levels of endogenous substances [26].
   Examples of endogenous substances may include hemoglobin, albumin, conjugated bilirubin, triglycerides, and high molecular weight DNA.
2. Exogenous substances may include Blood Collection Tube (BCT) additives or anticoagulants, expected treatment compounds (such as Tarceva or neupogen). Testing should also include exogenous substances introduced by the cfDNA extraction process, such as ethanol, xylene, beads, salts, etc.
   Microorganism testing may be considered, depending on the assay's intended use and specimen type, but may not be required.
3. Interference by germline DNA may be considered as an interfering substance or it may simply be addressed as a bioinformatics challenge.

9.3. Minimal Experimental Design

1. Include at least 1 variant positive specimen (at 1x-1.5x LoD) and 1 WT sample (derived from normal donors) to evaluate the effect of each potential interferent and to assess potential false positives in the presence of potentially interfering substances. Use contrived samples, as appropriate. Testing may be completed at one site; instrument and operator are not factors in this study design.
2. Test all specimens with and without (control group) each interferent present. Use contrived samples at levels close to LoD (at 1x-1.5x LoD). Include WT samples (derived from normal donors) to assess potential false positives in the presence of each interferent.
3. It is recommended that developers work with a statistician to determine the minimal replicate number required, based on assay performance.
4. Interfering substances may be tested individually or pooled to reduce the total number of samples to be tested.

9.4. Statistical Analysis

Visually inspect the plotted data and assess whether there is systematic bias (difference) between the selected specimens with interferent and the control group. If there is, evaluate the range of the difference and compare it to the interference criteria (typically clinical meaningful, pre-established criteria).

BloodPAC
info@bloodpac.org | © 2017 BloodPAC
BloodPAC is a consortium managed by the Center for Computational Science Research, Inc., which is an Illinois based not-for-profit corporation

9.5. Data Presentation Format

Table 6 Simple statistics and difference between the control and interferent tests.

Variants	Control Allele Frequency (Crtl.)			Interferent Allele Frequency (Interf.)			Difference from Control
	N	Mean	SD	N	Mean	SD	
COSM476	6	0.105	0.022	5	0.098	0.012	-7.20%
COSM6224	6	0.18	0.024	6	0.173	0.012	-4.00%

Table 7 APA* and ANA* Statistics

Statistic	Sample/Variant(s)			
	Sample 1/ COSM476	Sample 5/ COSM516	Wild-type	All Samples
# pos by both	5	5		43
# pos, ctrl	5	6		46
# pos, interf	5	6		45
APA	100.0%	83.30%	NA	94.50%
# pos, ctrl (No Call excluded)	5	5		43
# pos, interf (No Call excluded)	5	6		45
APA (No Call excluded)	100.0%	90.90%	NA	97.70%
# neg by both	1914	2499	3571	25861
# neg, ctrl	1933	2863	3582	26428
# neg, interf	2122	2541	3624	26223
ANA	94.4%	92.50%	99.10%	98.20%
# neg, ctrl(No Call excluded)	1914	2500	3571	25863
# neg, interf (No Call excluded)	1914	2499	3571	25861
ANA (No Call excluded)	100.0%	99.98%	100.00%	99.97%

* APA and ANA are weighted averages of PPA and NPA. A more in-depth description of APA and ANA can be found in CLSI ILA28 [43].

10. Protocols for Guardbanding Class III Medical Devices

10.1. Introduction

The objective of guardbanding studies is to demonstrate the robustness of the assay to detect cfDNA variants across a range of factors that may affect sample level, quality, and assay performance. The demonstration of robustness is necessary to determine the range of parameters in which the assay can detect and quantify the variants included in the intended use and due to the specific challenges posed by cfDNA samples. In particular, cfDNA derived from tumor is present in the circulation at very low levels, especially in comparison to germline-based cfDNA present in the circulation. In addition, the levels of cfDNA are known to vary widely by patient and are influenced by the patient's disease type, stage, and treatment status. The levels and quality of cfDNA may also be highly vulnerable to preanalytical factors surrounding blood collection, such as BCT type, blood storage temperature and transport conditions, and time to plasma collection. Because of these factors, the range in which the assay is robust and critical parameters around patient factors and specimen collection, and sample handling must be identified so that the risk to performance can be managed.

An example study designs are described below; however, minimal sample size should be determined for each assay based on its performance goal. The minimal sample size should sufficiently address the uncertainty of the performance estimates to support the claims. Assay developers may find it useful to leverage in *silico analysis* methods to perform guardbanding studies around iterative changes in the assay design during the development process and prior to assay lock.

10.2. Experimental Protocols: Identifying Variables

1. Assay developers will need to identify the most critical steps in their specimen collection and NGS process based on their chosen assay techniques. Developers should use risk assessment to guide this process.
2. Recommendation: Guardbanding studies completed by the BloodPAC Pre-analytics Working Group have been completed to determine the effect of factors around specimen collection such as blood draw volumes, hemolysis, multiple tube draws, time between draws, and spin speeds, which may affect cfDNA yield and quality.
    - Consider blood collection as step 1 in the protocol. For example, the USC team has done work in this area [29].
3. Other "standard" guardbanding variables to consider:
    - Amplification Time
    - Incubation Time

info@bloodpac.org | © 2017 BloodPAC
BloodPAC is a consortium managed by the Center for Computational Science Research, Inc., which is an Illinois based not-for-profit corporation

- Elution Time
- Temperature variation for critical assay steps
- Sample volume
- DNA input amount (see below)

Testing DNA input as a guard banding variable enables the developer to evaluate the performance of the assay at DNA inputs other than the standard input, at which the LoD was determined. Detection may be observed even at low inputs, but this input may be below the level of maximal performance.

Other considerations around DNA input: Developers may find that there is a risk of false positives at too high DNA input.

Note: Select members of Blood PAC have tested ranges of 1-50ng or even 100ng DNA inputs. The key objective is to determine the input that maximized performance and minimized the risk of interference.

10.3. Minimal Experimental Design

1. Include at least 1 variant positive specimen (at 1x-1.5x LoD) and 1 WT samples (derived from normal donors) to assess potential false positives under all altered test conditions. Use contrived samples, as appropriate.
2. Test all specimens run with each altered condition and run using the standard condition.
3. It is recommended that developers work with a statistician to determine the minimal replicate number required, based on the assay performance goal. The minimal sample size should sufficiently address the uncertainty of the performance estimates to support the claims.

10.4. Statistical Analysis

An ANOVA will be performed to compare the assay performance run under the standard conditions and among the altered conditions tested to assess robustness. The mean and SD of each condition will also be determined.

10.5. Data Presentation Format

BloodPAC
info@bloodpac.org | © 2017 BloodPAC
BloodPAC is a consortium managed by the Center for Computational Science Research, Inc., which is an Illinois based not-for-profit corporation

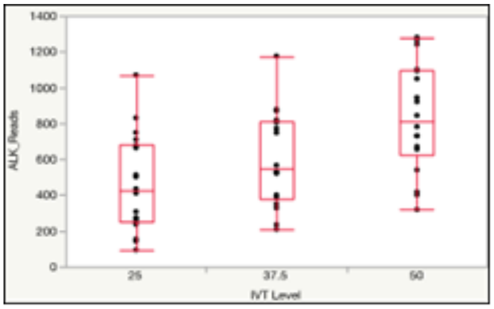

Figure 4 Example Box Plot of ALK Fusion Assay within three IVT guard band conditions

Table 8 Example Simple statistics and 95% confidence intervals for ALK Fusion Assay within three IVT guard band conditions

ALK Fusion Reads		N	Mean	Std Dev	Lower 95%CI	Upper 95% CI
Guard Band Condition	25 IVT	18	461	273	325	596
	37.5 IVT	18	607	265	476	739
	50 IVT	18	833	302	682	983

11. Protocols for Evaluation of Prepared Specimen Stability

11.1. Introduction

Stability Studies discussed below focus only on Prepared Specimen Stability, as the AV WG believes that protocols for Kit stability are well established in other guidelines, such as CLSI EP25 [30]. Specimens in all formats listed in the assay's intended use, as well extracted cfDNA, should be considered for specimen stability evaluation. In addition to prepared specimen stability, assay developers should also demonstrate stability from whole blood specimens, as appropriate with the BCT selection and assay specifications.

Individual sponsors who have a centralized testing model in which specimens, such as stabilized whole blood in BCTs or frozen plasma, are shipped prior to testing should assess the shipping stability of specimens. In this case, specimens should be exposed to a sequence of stress conditions that mimic various shipping scenarios (for example, summer

shipping, international shipping, humidity, vibration, etc.) before placement on test for stability. Individual sponsors should refer to CLSI EP25 for guidance on the design of this protocol [30]. An example study design is described below; however, minimal sample size should be determined for each assay based on its performance goal. The minimal sample size should sufficiently address the uncertainty of the performance estimates to support the claims.

11.2. Experimental Protocols

1. 3 prepared variant positive specimens and 1 WT specimen (derived from normal donors)
2. Variant positive specimens selected should be at or near LoD (1x-1.5x LoD) to assess stability.
3. Test from baseline (0 months), in 3-month increment up to desired stability period (typically at a minimum of 6 months, and up to 24 months).

11.3. Statistical Analysis

A linear least squares regression analysis could be used to evaluate the stability of prepared specimens. The fitted regression lines, parameter estimates and associated 95% one-sided lower confidence intervals for the regression lines shall be obtained, along with the mean results at each time point and the acceptance criterion for the maximum slope drifts (typically pre-specified).

11.4. Data Presentation Format

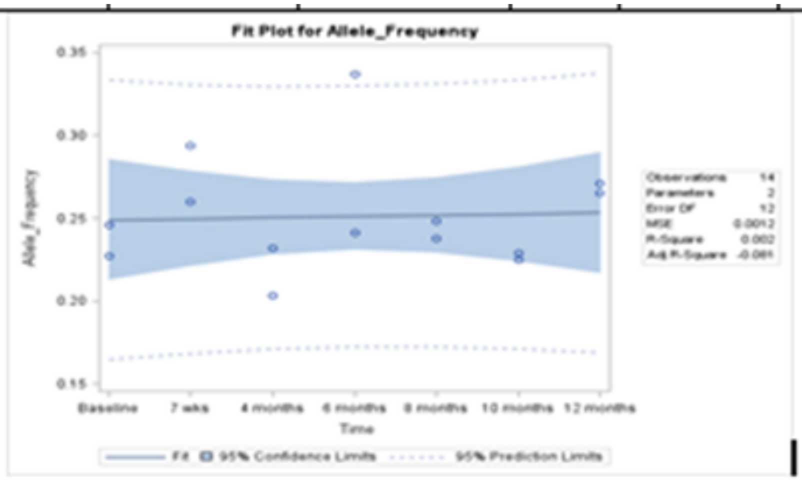

Figure 5 Linear regression of allele frequency of samples from stability tests.

BloodPAC
info@bloodpac.org | © 2017 BloodPAC
BloodPAC is a consortium managed by the Center for Computational Science Research, Inc., which is an Illinois based not-for-profit corporation

Table 9 Linear regression of allele frequency of samples from stability tests.

Sample	Expected Variant	Intercept	Slope_Time	Slope_Pvalue	NObs	NPositive	Baseline	Criterion	Time
AD1000	COSM6223	0.249	0.00071429	0.88074	14	14	0.2365	0.16555	373
AD1000	COSM6223	0.23788	-0.0021964	0.58884	14	14	0.247	0.1729	373

BloodPAC
info@bloodpac.org | © 2017 BloodPAC
BloodPAC is a consortium managed by the Center for Computational Science Research, Inc., which is an Illinois based not-for-profit corporation
.

SECTION III: BLOODPAC AV WG STANDARD METHODS

1. PREPARATION OF CONTRIVED SAMPLES USING CFDNA FROM CELL CULTURE MEDIA

1.1. OBJECTIVE OF THIS STANDARD METHOD

The objective of this method is to prepare contrived cfDNA samples that are representative of the DNA fragment size and the intended matrix of true patient samples using cfDNA extracted from cell culture media. These samples may be used to supplement clinical samples in selected analytical validation studies, after equivalence is shown through the CSFC study discussed above. The method described below is preferred for producing contrived samples as cfDNA obtained from cell culture media closely simulates the apoptotic fragment size distribution of cfDNA derived from true patient matrix [41].

1. Select mutant-positive cell lines that contain the variants to be assessed in the new assay
2. Culture the cell lines using the appropriate media using the processing conditions specified in Bronkhurst et al. 2016 [41].
3. Collect the cell culture media as described in Bronkhurst et al. 2016 [41].
4. Extract the cell line cfDNA from the cell culture media using the appropriate extraction method and process that aligns with the assay's intended use.
5. Assess the DNA fragment size of the extracted mutant-positive cell line cfDNA by using a Bioanalyzer or similar fragment analysis method to ensure that the fragment sizes represent those derived from patient samples (165-175bp). Retain an image of the Bioanalyzer profile of the extracted cell line media cfDNA.
6. Quantify the mutant and WT (wild type) molecules of the target(s) of interest in the extracted the cell line cfDNA using an appropriate orthogonal method. Alternatively, developers may choose to use an internal method for intermediate quantification steps on pool components as long as the final contrived sample is measured by the orthogonal method.
7. Store the quantified cell line cfDNA stock(s) for later spiking into plasma-derived cfDNA.
8. To obtain a pool of variant negative cfDNA to serve as the background for the contrived samples, select variant-negative individuals with cancer or risk-matched normal donors. Donors should have previously been determined be wild type at each locus of interest using the orthogonal method. Collect plasma from these donors using an appropriate BCT that aligns with the new assay's intended use.
9. Extract each variant-negative donor using the appropriate extraction method and process that aligns with the assay's intended use to ensure that the matrix is representative of true patient samples.
10. Pool the resulting plasma-derived cfDNA.

BloodPAC
info@bloodpac.org | © 2017 BloodPAC
BloodPAC is a consortium managed by the Center for Computational Science Research, Inc., which is an Illinois based not-for-profit corporation

11. Assess the DNA fragment size of the extracted plasma-derived cfDNA pool before spiking by using a Bioanalyzer or similar fragment analysis method to ensure that the fragment sizes represent those derived from patient samples (165-175bp). Retain an image of the Bioanalyzer profile of the plasma-derived cfDNA before spiking.
12. Quantify the WT (wild type) molecules of the target(s) of interest the extracted the plasma-derived cfDNA using an appropriate orthogonal method or internal method, as described above.
13. Using the quantified values obtained above, spike the mutation-positive, extracted cell line cfDNA into the variant-negative, extracted cfDNA from plasma at appropriate ratios to attain the target mutant allelic fraction (MAF) within +/-20 to 30% of the targeted final value. Note that it is not advised to produce contrived samples at MAFs below 0.1%, as reproducibility cannot be controlled at those low levels due to sampling/pipetting effects. Retain some "unspiked" variant-negative, plasma-derived cfDNA for MAF adjustments, if necessary.
14. Quantify the mutant and WT (wild type) molecules of the target(s) of interest new contrived cfDNA sample using an appropriate orthogonal or internal method.
1. Adjust the MAF of the sample by re-spiking with cell line cfDNA or by adding more variant-negative cfDNA, as needed. The final MAF of the pool must be confirmed using the appropriate orthogonal method even if intermediates were quantified using an internal method.
15. To ensure that background and the fragment size of the final contrived sample mimics that of the clinical samples, use a Bioanalyzer or similar fragment analysis method to demonstrate that the final profile matches that of expected of clinical samples. Retain an image of the final Bioanalyzer profile.
16. The concentration of the final contrived samples should be quantified and then may be diluted as needed to attain a cfDNA concentration or yield that is consistent with that of patient samples in the intended use population.
17. Representative contrived samples can then be aliquoted and frozen for use in the CFSC study. Contrived samples used in AV studies should be different from those prepared and used for assay development.

2. Preparation of Contrived DNA Samples using Fragmented Cell line Genomic DNA

2.1. Objective of this Standard Method

The objective of this method is to prepare contrived cfDNA samples that are representative of the DNA fragment size and the intended matrix of true patient samples. These samples may be used to supplement clinical samples in selected analytical validation studies, after equivalence is shown through the CSFC study discussed above.

2. Select mutant-positive cell lines that contain the variants to be assessed in the new assay
3. Fragment the mutant positive cell line DNA to mimic the size/fragment length of cfDNA derived from patient samples (165-175bp). Note that the choice of fragmentation method may introduce spurious variants into the cell line DNA. Additional assessments of the cell line DNA post-fragmentation may be done if the introduction of additional variants is a concern.
4. Assess the DNA fragment size of the fragmented mutant-positive cell line DNA by using a Bioanalyzer or similar fragment analysis method to ensure that the fragment sizes represent those derived from patient samples (165-175bp). Retain an image of the Bioanalyzer profile.
18. Quantify the mutant and WT (wild type) molecules of the target(s) of interest in the fragmented cell line DNA using an appropriate orthogonal method. Alternatively, developers may choose to use an internal method for intermediate quantification steps on pool components as long as the final contrived sample is measured by the orthogonal method.
5. Store the quantified, fragmented cell line DNA stock(s) for later spiking into plasma.
6. Select variant-negative individuals with cancer or risk-matched normal donors. Donors should have previously been determined be wild type at each locus of interest using the orthogonal method. Collect plasma from these donors using an appropriate BCT that aligns with the new assay's intended use. Variant-negative plasma may be pooled as needed to have sufficient volume to complete steps 7-10 below.
7. To have a pool of variant negative cfDNA for later MAF adjustments, extracted the pooled plasma retained in step 7 above using the appropriate extraction method and process that aligns with the assay's intended use. Retain a sufficient volume of the plasma pool for spiking.
8. Quantify the WT (wild type) molecules of the target(s) of interest in the pool, variant-negative cfDNA extracted from plasma using an appropriate orthogonal or internal method as described above.
9. Assess the DNA fragment size of the extracted plasma-derived cfDNA pool before spiking by using a Bioanalyzer or similar fragment analysis method to ensure that the fragment sizes represent those derived from patient samples (165-175bp). Retain an image of the Bioanalyzer profile of the plasma-derived cfDNA before spiking.
10. Using the quantification values obtained above, spike the mutation-positive, fragmented cell line DNA into an aliquot of the variant-negative plasma retained above in step 7 at appropriate ratios to attain the target mutant allelic fraction (MAF). Note that it is not advised to produce contrived samples at MAFs below 0.1%, as reproducibility cannot be controlled at those low levels due to

info@bloodpac.org | © 2017 BloodPAC
BloodPAC is a consortium managed by the Center for Computational Science Research, Inc., which is an Illinois based not-for-profit corporation

sampling/pipetting effects. At this step, it is advisable to "over spike" the mutant target to allow for differences in recovery after extraction. The sample MAF can then be adjusted down after extraction. Retain some "unspiked" variant-negative, plasma that can be used as a source of negative cfDNA for adjustments, if necessary.

11. Extract the spiked-in plasma samples from step 9 above using the appropriate extraction method and process that aligns with the assay's intended use.
12. Quantify the mutant and WT molecules of the target(s) of interest in the extracted spiked-in sample using an appropriate orthogonal method.
13. Adjust the MAF of the sample by re-spiking with fragmented cell line DNA or by adding more variant-negative cfDNA, as needed to attain a final MAF within +/-20 to 30% of the targeted final value. The final MAF of the pool must be confirmed using the appropriate orthogonal method even if intermediates were quantified using an internal method.
14. To ensure that background and the fragment size of the final contrived sample mimics that of the clinical samples, use a Bioanalyzer or similar fragment analysis method to demonstrate that the final profile matches that of expected of clinical samples. Retain an image of the final Bioanalyzer profile of the final, spiked sample.
15. The final contrived samples may be diluted as needed to attain a cfDNA concentration or yield that is consistent with that of patient samples in the intended use population.
16. Representative contrived samples can then be aliquoted and frozen for use in the CFSC study. Contrived samples used in AV studies should be different from those prepared and used for assay development.

3. Preparation of AcroMetrix™ Oncology Hotspot Control Reference material

3.1. Objective of this Standard Method

To prepare the AcroMetrix™ Oncology Hotspot Control material for SNVs/small InDels, Fusions, and CNVs

Note: The protocols in this section represent those currently practiced by Thermo Fisher Scientific for preparing cfDNA reference materials and has been included here as an example process used by a manufacturer of reference/control materials. Assay developers who are preparing contrived materials in their labs for use in various analytical validation protocols should refer to Standard Methods 1 and 2.

3.2. Standard Protocol: Preparation of Contrived Samples for Lung cfDNA Assay Development | SNVs

BloodPAC
info@bloodpac.org | © 2017 BloodPAC
BloodPAC is a consortium managed by the Center for Computational Science Research, Inc., which is an Illinois based not-for-profit corporation
.

1. AcroMetrix™ Oncology Hotspot Control material, which contains 40 engineered variants covered by the Oncomine Lung cfDNA Assay, in a genomic DNA (gDNA) background GM24385 from Coriell Institute for Medical Research. See **Appendix C: Targets Included in AcroMetrix® Oncology Hotspot Control** for a complete list of variants included.
2. Serially diluted at 0.1% and 0.5% MAF's.
3. Both AcroMetrix™ Oncology Hotspot Control material and GM24385 gDNA were fragmented by sonication to mimic the size of cfDNA (165-175 bp). It is recommended that developers use a Bioanalyzer to demonstrate that the DNA fragmentation profile of any prepared contrived sample is comparable to the DNA size profile of cfDNA derived from plasma samples.
4. Currently, efforts are being made to commercialize this reference material.
5. For Analytical Validation Protocols, the AV WG recommends working in a range of DNA/RNA input that is representative of actual disease states, e.g., 50ng, as well as being representative of the assay in question.

3.3. Standard Protocol: Preparation of Contrived Samples for lung cfDNA Assay Development | FUSIONS

1. Titrate total RNA (~1%) from fusions positive cell lines into cell free nucleic acid (cfDNA+cfRNA) background extracted from healthy donor plasma samples.
2. Configure assay for detection of ALK, RET and ROS driver genes for lung cancer assay development.

3.4. Standard Protocol: Preparation of Contrived Samples for lung cfDNA Assay Development | CNVs

1. cfDNA was extracted from cell free media of cultured cells harboring CNV targets titrated into cfDNA background extracted from healthy donor plasma.
2. Target configurations included CCND1, EGFR, MET, ERBB2, MYC and FGFR1 amplifications.

4. Standard Method for Collecting Normal Human Plasma

4.1. Objective(s) for this Standard Method

A standardized approach for the collection of healthy human plasma for use in the development and analytical validation of cfDNA/cfRNA assays must be both safe and effective. Inability to follow appropriate safety precautions for handling human donor plasma may lead to an inadvertent serious infection. Inability to secure an effective and standardized supply of healthy human plasma can seriously derail/delay the development of a cfDNA/cfRNA assay

BloodPAC
info@bloodpac.org | © 2017 BloodPAC
BloodPAC is a consortium managed by the Center for Computational Science Research, Inc., which is an Illinois based not-for-profit corporation

4.2. Standard Protocols

1. Suggestion: Use of normal donors to provide the plasma, 200 mL per donor, is possible. If donors are to be pooled, it recommended to minimize the total number of donors that are included in one pool. If additional sample is needed, it is preferable to pool serial collections from the same donor than to pool multiple donors.
2. "Normal Donors" will be defined by the intended use population. For example, you may collect an exercise cohort before and after exercise or a cohort of active smokers who do not have lung cancer, or a cohort of patients with inflammatory disease.
3. Collect blood in the appropriate BCT for assay's intended use (for example, Streck or K2EDTA).
4. Process the blood to plasma. A suggested double-spun protocol for obtaining cell-free plasma is provided below:
   i. Collect plasma within 4 hours of blood draw
   ii. Centrifuge the blood sample at 2000 × g for 10 minutes at 4°C
   iii. After the first centrifugation, carefully transfer the plasma to a new centrifuge tube, taking care not to disturb the buffy coat layer.
      1. Centrifuge the plasma sample at 16,000 × g for 10 minutes at 4°C. Alternatively, the plasma sample can be centrifuged at 6000 × g for 30 minutes at 4°C.
      2. Transfer the supernatant to a fresh tube and note the plasma volume. Proceed directly to cfDNA extraction or freeze the plasma at -80°C.

5. Standard Method for the Preparation of Patient Sample Pools

In chemistries and/or studies where contrived material derived from cell lines is not comparable or equivalent to patient samples (e.g. due to efficiency in recovery of input molecules or fragmentation patterns), it is possible to create pools of variants comprised of patient cfDNA.

Note 1: Pools should not be made across cancer types. Ideally, pools should be made within each cancer type and certain variant types. If pooling is done across cancer types, then developers should demonstrate that results/conclusions have not been impacted.

Note 2: Accountability of pooling is important to track as it helps calculate allelic frequencies in the pool.

- Quantify variant of interest in a patient cfDNA, at the DNA molecule level, and confirm negative status at other variants of interest
  - quantification methods include dPCR or targeted NGS
  - measuring: mutant molecules per uL, wild type molecules per uL

- Quantify wild type DNA molecules per uL in diluent cfDNA (can be from healthy donors or wild type patient samples)
- Calculate appropriate volume of each patient cfDNA and wild type cfDNA to add to pool in order to achieve targeted variant allele frequency.
  Example:
  - Sample 1:
    - SNV1 at 100 mutant molecules per uL and 900 wildtype molecules per uL
    - SNV2 at 1000 wild type molecules per uL
  - Sample 2:
    - SNV1 at 2000 wild type molecules per uL
    - SNV2 at 200 mutant molecules per uL, 1800 wild type molecules per uL
  - 10 uL of sample 1 + 5 uL of sample 2
    - SNV1 at 5% VAF
    - SNV2 at 5% VAF
- Confirm that targeted frequencies have been reached through quantification step (dPCR or NGS)
- This stock may then be diluted in wild type cfDNA for subsequent experiments that need particular multiples of LoD.

BloodPAC
info@bloodpac.org | © 2017 BloodPAC
BloodPAC is a consortium managed by the Center for Computational Science Research, Inc., which is an Illinois based not-for-profit corporation
.

SECTION IV: REFERENCES CITED

1. CLSI: Defining, Establishing, and Verifying Reference Intervals in the Clinical Laboratory; Approved Guideline-Third Edition. CLSI document EP28-A3C. Wayne, PA: Clinical and Laboratory Standards Institute, 2005.
2. CLSI. Protection of Laboratory Workers from Occupationally Acquired Infections; Approved Guideline-Third Edition. CLSI document M29-A3. Wayne, PA: Clinical and Laboratory Standards Institute, 2010.
3. Mandel, P., and P. Metais. "Circulating nucleic acid." *Acad Sci Paris* 142 (1948): 241-3.
4. Leon, S. A., et al. "Free DNA in the serum of cancer patients and the effect of therapy." *Cancer research* 37.3 (1977): 646-650.
5. Stroun, M., et al. "Neoplastic characteristics of the DNA found in the plasma of cancer patients." *Oncology* 46.5 (1989): 318-322.
6. Diehl, Frank, et al. "Detection and quantification of mutations in the plasma of patients with colorectal tumors." *Proceedings of the National Academy of Sciences of the United States of America* 102.45 (2005): 16368-16373.
7. Diehl, F. et al. Circulating mutant DNA to assess tumor dynamics. Nat. Med. 14, 985-990 (2008).
8. Oncomine™ cfDNA Assay Statistical Methods White Paper: Analytical verification methods for the Oncomine Lung cfDNA Assay using the Ion S5 XL System
9. Yu, H. A. et al. Analysis of tumor specimens at the time of acquired resistance to EGFR-TKI therapy in 155 patients with EGFR-mutant lung cancers. Clin. Cancer Res. Off. J. Am. Assoc. Cancer Res. 19, 2240–2247 (2013).
10. Overman, M. J. et al. Use of research biopsies in clinical trials: are risks and benefits adequately discussed? J. Clin. Oncol. Off. J. Am. Soc. Clin. Oncol. 31, 17–22 (2013)
11. Lokhandwala, T. et al. Costs of Diagnostic Assessment for Lung Cancer: A Medicare Claims Analysis. Clin. Lung Cancer 18, e27–e34 (2017)
12. Oxnard, G. R. et al. Association between plasma genotyping and outcomes of treatment with osimertinib (AZD9291) in advanced non-small-cell lung cancer. J. Clin. Oncol. 34(28):3375-3382 (2016)
13. National Comprehensive Cancer Network. Non-Small Cell Lung Cancer (Version 2.2018). Accessed February 8, 2018
14. Lindeman, N. I. et al. Updated molecular testing guideline for the selection of lung cancer patients for treatment with targeted tyrosine kinase inhibitors. Arch Pathol Lab Med. 2018; 142(3):321-346.
15. Kesselheim, Aaron S., and Jerry Avorn. "New "21st Century Cures" legislation: speed and ease vs science." *JAMA* 317.6 (2017): 581-582
16. CLSI: Evaluation of Detection Capability for Clinical Laboratory Measurement Procedures, 2nd Edition, CLSI document EP17-A2. Wayne, PA: Clinical and Laboratory Standards Institute; 2012

BloodPAC
info@bloodpac.org | © 2017 BloodPAC
BloodPAC is a consortium managed by the Center for Computational Science Research, Inc., which is an Illinois based not-for-profit corporation

17. Hu, Yuebi, et al. "False-positive plasma genotyping due to clonal hematopoiesis." Clinical Cancer Research 24.18 (2018): 4437-4443.
18. CLSI: User Protocol for Evaluation of Qualitative Test Performance-Second Edition, CLSI EP-12-A2.
19. New York State Next Generation Sequencing guidelines for somatic variant detection (Jan 2018)
20. CLSI: Evaluation of the Linearity of Quantitative Measurement Procedures: A Statistical Approach, Approved Guideline, EP06-AE: Clinical and Laboratory Standards Institute, 2003.
21. Schmiegel et al. (2017): Mol Oncol. 11(2) : 208 – 219
22. Saunders et al. (2016): Annals of Oncology 27 (6) : 149 – 206
23. Vidal et al. (2017): J Clin Oncol 35 (suppl 4S; Abstract 607)
24. Oxnard et al. (2016) J Clin Oncol 34(28):3375-3382; Baselga et al. (2015): Oral presentation. SABCS, Abstract S 6 – 01
25. CLSI: Evaluation of Precision of Quantitative Measurement Procedures. CLSI document EP-05A3. Wayne, PA: Clinical and Laboratory Standards Institute
26. CLSI: Interference Testing in Clinical Chemistry; EP7-A2, 2nd edition, Wayne, PA, 2005.
27. Jaiswal, Siddhartha, et al. "Age-related clonal hematopoiesis associated with adverse outcomes." *New England Journal of Medicine* 371.26 (2014): 2488-2498.
28. Steensma, David P., et al. "Clonal hematopoiesis of indeterminate potential and its distinction from myelodysplastic syndromes." *Blood* 126.1 (2015): 9-16.
29. Rodríguez-Lee, Mariam, et al. "Effect of Blood Collection Tube Type and Time to Processing on the Enumeration and High-Content Characterization of Circulating Tumor Cells Using the High-Definition Single-Cell Assay." Archives of pathology & laboratory medicine (2017).)
30. CLSI: Evaluation of Stability of In Vitro Diagnostic Reagents, CLSI document EP25-A, Wayne, PA, 2009.
31. Merker, Jason D., et al. "Circulating Tumor DNA Analysis in Patients With Cancer: American Society of Clinical Oncology and College of American Pathologists Joint Review." Archives of pathology & laboratory medicine (2018).
32. Next-generation Sequencing: Standardization of clinical testing (NEXSToCT)-Supplementary guidelines. Biotechnology:doi:10,1038/hbt.2403.
33. MolDx: Analytical Performance Specifications for Qualitative Tumor-Only Somatic Variant Detection using Circulating Tumor DNA (M00135, V2)-Checklist.
34. Miller MJ, et al.; National Center for Emerging and Zoonotic Infectious Diseases, CDC, Guidelines for safe work practices in human and animal medical diagnostic laboratories. MMWR Surveil Summ. 2012; 61:1-102.
35. Guidance for Industry and FDA staff, Statistical Guidance on Reporting Results from Studies Evaluating Diagnostic Tests. March 13, 2007

36. Xue, Xiaoyan, et al. "Optimizing the yield and utility of circulating cell-free DNA from plasma and serum." *Clinica Chimica Acta* 404.2 (2009): 100-104.
37. CLSI MM09-A2. Nucleic Acid Sequencing Methods in Diagnostic Laboratory Medicine; Approved Guideline, MM09A2.
38. Jennings, Lawrence J., et al. "Guidelines for validation of next-generation sequencing–based oncology panels: a joint consensus recommendation of the Association for Molecular Pathology and College of American Pathologists." The Journal of Molecular Diagnostics 19.3 (2017): 341-365.
39. Summary of Safety and Effectiveness Data of the FoundationOne CDx test (P170019).
40. Summary of Safety and Effectiveness Data of the Thermo Fisher Oncomine Dx Target Test PMA submission (P160045).
41. Bronkhorst, Abel Jacobus, et al. "Characterization of the cell-free DNA released by cultured cancer cells." Biochimica et Biophysica Acta (BBA)-Molecular Cell Research 1863.1 (2016): 157-165.
42. Statistical Guidance on Reporting Results from Studies Evaluating Diagnostic Tests - Guidance for Industry and FDA Staff, March 2007https://www.fda.gov/regulatory-information/search-fda-guidance-documents/statistical-guidance-reporting-results-studies-evaluating-diagnostic-tests-guidance-industry-and-fda
43. CLSI: Quality Assurance for Design Control and Implementation of Immunohistochemistry Assays Approved Guideline-Second Edition, CLSI I/LA-28-A2.
44. Prevalence of clonal hematopoiesis of indeterminate potential (CHIP) measured by an ultra-sensitive sequencing assay: exploratory analysis of the Circulating Cell-free Genome Atlas (CCGA) study. ASCO, June 5, 2018

info@bloodpac.org | © 2017 BloodPAC
BloodPAC is a consortium managed by the Center for Computational Science Research, Inc., which is an Illinois based not-for-profit corporation

APPENDIX A: WORKED EXAMPLE:
Cell Free DNA Limit of Blank (LoB) Study (example)

EXECUTIVE SUMMARY

This study was conducted following study protocol PCPXXXXX to assess the Limit of Blank (LoB) of the Oncomine™ Cell Free Assay by analyzing the false positive rate in a set of samples known to be wild type at each variant location that can be detected by the test. Affirming a LoB at or close to zero is important as it enables clinicians to discern between low-level true positives and false positives that manifest in the form of background noise. A low LoB with respect to overall signal level is an important aspect in achieving specific diagnoses.

In this study, operators extracted cell free DNA (cfDNA) from *60 different healthy donor blood plasma specimens* and carried these extractions through sequencing. The study was conducted using two different lots of Oncomine™ Cell Free Assay. For each kit lot, each plasma sample was extracted once and made into single cfDNA libraries. Operators sequenced each library in duplicate, thus a total of 120 libraries were sequenced per kit lot.

The result at each variant location for each sample was tabulated. For all five samples, there were no positive calls at any of the variant locations or for any of the fusion targets. The false positive rate of the test is thus determined to be zero out of the total of XXX SNV and deletion locations confirming that the LoB of the Oncomine™ Cell Free Assay is zero.

PURPOSE

The purpose of this study was to demonstrate that the LoB meets the specifications with respect to each kit lot, and that the false positive rate (no calls excluded) at each somatic variant location shall be less than 5%, corresponding to a probability of Type I error (i.e. false positive rate) of no greater than 5% at the LoB.

Samples that are WT at all variant locations (Blanks) were used to confirm no variant calls were made at any variant locations targeted by the test. Variant calling software provides only three possible results [variant detected, variant not detected, or no information provided (no call)]. Negative samples have essentially no distribution

because all such results are typically reported as variant not detected, and by definition (EP17-A2), the 95th percentile of test results on blank samples equals zero.

SCOPE

The goal of this study was to assert that the LoB was equal to zero for all XXX variant locations that the test can detect; including those variants with clinical and analytical claims from sixty different healthy donor blood plasma specimens produce WT calls at all targeted variant locations.

DEFINITIONS

Term	Abbreviation	Definition
Allele or Allelic Frequency	AF	The proportion of a particular allele (variant of a gene) among all allele copies being considered
Base Pair	BP or bp	A pair of complementary bases in a double-stranded nucleic acid molecule
Diagnostic	Dx	Used to help identify a disease, illness, or problem.
False Positive	FP	A test result which incorrectly indicates that a particular condition or attribute is present
Cell Free DNA	cfDNA	DNA which has been released from cells and is circulating in the bloodstream
In Vitro Diagnostic	IVD	Tests that can detect diseases, conditions, or infections
In Vitro Transcript	IVT	RNA produced enzymatically from ds DNA template
Insertions/ Deletions	InDel	Insertion or deletion of DNA sequences relative to the reference sequence
Limit of Blank	LoB	The highest measurement result that is likely to be observed with a blank (or WT) sample of a given target.
Mutant	MUT	A new genetic character arising or resulting from an instance of mutation
Negative Percent Agreement	NPA	A statistical method of comparing negative results to determine agreement between tests.
Next Generation Sequencing	NGS	A set of new high-throughput, low-cost sequencing technologies

BloodPAC
info@bloodpac.org | © 2017 BloodPAC
BloodPAC is a consortium managed by the Center for Computational Science Research, Inc., which is an Illinois based not-for-profit corporation

Table 1: Results by Variant Location (sample snippet)

	Lot A						Lot B						Overall					
variant	n	pos.call	neg.call	no.call	nocall.rate	fp.rate	n	pos.call	neg.call	no.call	nocall.rate	fp.rate	n	pos.call	neg.call	no.call	nocall.rate	fp.rate
**SNVs and Deletions**																		
**COSM1185313**	60	0	60	0	0.00%	0.00%	60	0	60	0	0.00%	0.00%	120	0	120	0	0.00%	0.00%
**OM3148**	60	0	60	0	0.00%	0.00%	60	0	60	0	0.00%	0.00%	120	0	120	0	0.00%	0.00%
**COSM20417**	60	0	59	1	1.67%	0.00%	60	0	58	2	3.33%	0.00%	120	0	117	3	2.50%	0.00%
**COSM1686998**	60	0	59	1	1.67%	0.00%	60	0	58	2	3.33%	0.00%	120	0	117	3	2.50%	0.00%
**COSM1560108**	60	0	59	1	1.67%	0.00%	60	0	60	0	0.00%	0.00%	120	0	119	1	0.83%	0.00%
**COSM1134662**	60	0	55	5	8.33%	0.00%	60	0	60	0	0.00%	0.00%	120	0	115	5	4.17%	0.00%
**...**																		

BloodPAC
info@bloodpac.org | © 2017 BloodPAC
BloodPAC is a consortium managed by the Center for Computational Science Research, Inc., which is an Illinois based not-for-profit corporation
.

APPENDIX B: SUMMARY TABLE OF EXAMPLE EXPERIMENTAL DESIGNS ACROSS PROTOCOLS

Example study designs are summarized below.

*Minimal sample size must be determined by individual sponsors for each assay based on its performance goal

Note that FDA recommends use of clinical samples in key analytical validation studies as noted below (e.g., LoD confirmation, accuracy, precision, certain stability studies, etc.) to demonstrate the performance of the test

Protocol	Section	Sample Type(s)	Sample source	Number of Unique Samples	Testing Sites	Operators/ Site	Minimum Levels/Sample	Minimum Reagent Lots	Instruments/ Sites	Minimum Total Days (Duration)	Other Factors	Total Reps/Sample/ Level within Reagent Lot per Condition	Total Samples per Reagent Lot*	Total*
**Reference Interval**	II:1	Negative Samples	Age and risk matched Normal Donors	120	1	1	1	1	1	1	N/A	120	120	120
**LoB**	II:2	Negative Samples	Age and risk matched Normal Donors and Mutation-Negative Patient Samples	~60 (see protocol)	1	1	1	2	1	1	N/A	TBD, as long as 60 total Blank samples are tested per Lot	60	120
**Contrived Sample Characterization Study**	II:3	Positive Samples	Serially diluted Patient Samples plus Contrived samples to be assessed	5	1	1	5	1	1	1	N/A	20	100	100
**LoD**	II:4	Low AF Positive Samples	Contrived Samples or Pooled	As needed to repres	1	1	5	2	1	N/A	N/A	10	50	100

**Protocol**	**Section**	**Sample Type(s)**	**Sample source**	**Number of Unique Samples**	**Testing Sites**	**Operators/ Site**	**Minimum Levels/Sample**	**Minimum Reagent Lots**	**Instruments/ Sites**	**Minimum Total Days (Duration)**	**Other Factors**	**Total Reps/Sample/ Level within Reagent Lot per Condition**	**Total Samples per Reagent Lot***	**Total***
			Clinical Samples for confirmation	ent the variants of interest										
**Analytical Accuracy**	II:5	WT (Negative) + Positive Samples	Clinical Samples	TBD*	1	1	1	1	1	1	N/A	TBD*	TBD*	TBD*
**Linearity**	II:6	Positive Samples	Contrived Samples or Pooled Clinical Samples	1	1	1	11	1	1	1	N/A	4	44	44
**Limit of Quantitation**	II:7	Low AF Positive Samples (around LoQ)	Contrived Samples or Pooled Clinical Samples	4	1	1	1	2	1	3	N/A	3	TBD*	TBD*
**Repeatability /Reproducibility**	II:8 i	Positive Samples, plus 1 WT	Clinical Samples	1	3	2	2	3	1	20	N/A	2 replicates/run	24	72
	II:8 ii		Clinical Samples	≥2	1 (for Single Site PMA)	2	2	3	3	20	N/A	2 replicates/run	72	144
	11.8 iii		Clinical samples with minimal volumes/ Contrived Samples	≥2	1 or multiple	4 instrument/ operator combinations	2	3	4 instrument/operator combinations	Multiple	Instrument /Operator confounded	2 replicates/run	8	24 (36 total if contrived) per sample, per site

Protocol	Section	Sample Type(s)	Sample source	Number of Unique Samples	Testing Sites	Operators/ Site	Minimum Levels/Sample	Minimum Reagent Lots	Instruments/ Sites	Minimum Total Days (Duration)	Other Factors	Total Reps/Sample/ Level within Reagent Lot per Condition	Total Samples per Reagent Lot*	Total*
**Interfering Substances**	II:9	1 WT (Negative) Samples + 1 Positive Sample (around 1-1.5x LoD)	Contrived Samples or Pooled Clinical Samples plus Normal Donor	2	1	1	1	1	1	1	Complete testing on Control Assay Condition +N Total Interfering Substances Conditions	TBD*	TBD*	TBD *
**Guard banding**	II:10	1 WT (Negative) Samples + 1 Positive Sample (around 1-1.5x LoD)	Contrived Samples or Pooled Clinical Samples plus Normal Donor	2	1	1	1	1	1	1	Complete testing on Control Assay Condition (no altered conditions) +N Total Assay Conditions	TBD*	TBD*	TBD *
**Prepared Specimen Stability**	II:11	1 WT (Negative) Samples + 3 Positive Sample (around 1-1.5x LoD)	Contrived Samples or Pooled Clinical Samples plus Normal Donor	4	1	1	1	1	1	Up to 24 months	Test at multiple time points from baseline (0 months), in 3-month increments.	TBD*	TBD*	TBD *

APPENDIX C: TARGETS INCLUDED IN ACROMETRIX® ONCOLOGY HOTSPOT CONTROL

Gene	Mutation CDS	Mutation AA	Mutation Type
MPL	c.1514G>A	p.S505N	SNV
MPL	c.1544G>T	p.W515L	SNV
MPL	c.1555G>A	p.A519T	SNV
NRAS	c.182A>G	p.Q61R	SNV
NRAS	c.174A>G	p.T58T	SNV
NRAS	c.112-70C>T	p.(=)	SNV
NRAS	c.52G>A	p.A18T	SNV
NRAS	c.35G>A	p.G12D	SNV
NRAS	c.29G>A	p.G10E	SNV
ALK	c.3824G>A	p.R1275Q	SNV
ALK	c.3522C>A	p.F1174L	SNV
MSH6	c.3246G>T	p.P1082P	SNV
MSH6	c.3261delC	p.F1088fs*2	DEL
MSH6	c.3300G>A	p.T1100T	SNV
MSH6	c.3438+14A>T	p.(=)	SNV
IDH1	c.395G>A	p.R132H	SNV
IDH1	c.388A>G	p.I130V	SNV
IDH1	c.367G>A	p.G123R	SNV
ERBB4	c.2791G>T	p.D931Y	SNV
ERBB4	c.2782G>T	p.E928*	SNV
ERBB4	c.1835G>A	p.R612Q	SNV
ERBB4	c.1828C>A	p.P610T	SNV
ERBB4	c.1784A>G	p.D595G	SNV
ERBB4	c.1089T>C	p.N363N	SNV
ERBB4	c.1022C>T	p.S341L	SNV
ERBB4	c.1003G>T	p.D335Y	SNV
ERBB4	c.909T>C	p.S303S	SNV
ERBB4	c.885T>G	p.H295Q	SNV
ERBB4	c.829C>A	p.H277N	SNV
ERBB4	c.804C>A	p.Y268*	SNV
ERBB4	c.730A>G	p.T244A	SNV
ERBB4	c.704C>T	p.A235V	SNV
ERBB4	c.633G>A	p.T211T	SNV
ERBB4	c.542A>G	p.N181S	SNV
ERBB4	c.515C>G	p.P172R	SNV
VHL	c.266T>A	p.L89H	SNV

Gene	Mutation CDS	Mutation AA	Mutation Type
NOTCH1	c.7412C>A	p.S2471*	SNV
NOTCH1	c.7386delC	p.A2463fs*14	DEL
NOTCH1	c.7375C>T	p.Q2459*	SNV
NOTCH1	c.7318C>T	p.Q2440*	SNV
NOTCH1	c.5033T>C	p.L1678P	SNV
NOTCH1	c.5025C>T	p.I1675I	SNV
NOTCH1	c.4799T>C	p.L1600P	SNV
NOTCH1	c.4793G>C	p.R1598P	SNV
NOTCH1	c.4778T>C	p.L1593P	SNV
NOTCH1	c.4721T>C	p.L1574P	SNV
RET	c.1852T>C	p.C618R	SNV
RET	c.1858T>C	p.C620R	SNV
RET	c.1894_1906>AGCT	p.E632_T636>SS	Complex
RET	c.1942G>A	p.V648I	SNV
RET	c.1991C>A	p.A664D	SNV
RET	c.2304G>C	p.E768D	SNV
RET	c.2307T>A	p.L769L	SNV
RET	c.2647_2648GC>TT	p.A883F	MNV
RET	c.2701G>A	p.E901K	SNV
RET	c.2753T>C	p.M918T	SNV
PTEN	c.19G>T	p.E7*	SNV
PTEN	c.40A>G	p.R14G	SNV
PTEN	c.49C>T	p.Q17*	SNV
PTEN	c.71A>G	p.D24G	SNV
PTEN	c.80-96A>G	p.(=)	SNV
PTEN	c.80A>G	p.Y27C	SNV
PTEN	c.112C>T	p.P38S	SNV
PTEN	c.142A>G	p.N48D	SNV
PTEN	c.156T>C	p.D52D	SNV
PTEN	c.163A>G	p.R55G	SNV
PTEN	c.166T>G	p.F56V	SNV
PTEN	c.202T>C	p.Y68H	SNV
PTEN	c.209+5G>A	p.?	SNV
PTEN	c.212G>A	p.C71Y	SNV
PTEN	c.227_228delAT	p.Y76fs*1	DEL
PTEN	c.245A>C	p.N82T	SNV

Gene	Mutation CDS	Mutation AA	Mutation Type
VHL	c.277G>C	p.G93R	SNV
VHL	c.286C>T	p.Q96*	SNV
VHL	c.296delC	p.P99fs*60	DEL
VHL	c.343C>A	p.H115N	SNV
VHL	c.353T>C	p.L118P	SNV
VHL	c.388G>C	p.V130L	SNV
VHL	c.431delG	p.G144fs*15	DEL
VHL	c.472C>G	p.L158V	SNV
VHL	c.481C>T	p.R161*	SNV
VHL	c.499C>T	p.R167W	SNV
VHL	c.506T>C	p.L169P	SNV
MLH1	c.1151T>A	p.V384D	SNV
CTNNB1	c.98C>G	p.S33C	SNV
CTNNB1	c.110C>T	p.S37F	SNV
CTNNB1	c.121A>G	p.T41A	SNV
CTNNB1	c.134C>T	p.S45F	SNV
FOXL2	c.402C>G	p.C134W	SNV
PIK3CA	c.35G>A	p.G12D	SNV
PIK3CA	c.93A>G	p.I31M	SNV
PIK3CA	c.180A>G	p.Q60Q	SNV
PIK3CA	c.210C>T	p.F70F	SNV
PIK3CA	c.323G>A	p.R108H	SNV
PIK3CA	c.331A>G	p.K111E	SNV
PIK3CA	c.344G>T	p.R115L	SNV
PIK3CA	c.536A>G	p.K179R	SNV
PIK3CA	c.971C>T	p.T324I	SNV
PIK3CA	c.1002C>T	p.L334L	SNV
PIK3CA	c.1035T>A	p.N345K	SNV
PIK3CA	c.1213T>C	p.S405P	SNV
PIK3CA	c.1258T>C	p.C420R	SNV
PIK3CA	c.1370A>G	p.N457S	SNV
PIK3CA	c.1616C>G	p.P539R	SNV
PIK3CA	c.1624G>A	p.E542K	SNV
PIK3CA	c.1633G>A	p.E545K	SNV
PIK3CA	c.1640A>G	p.E547G	SNV
PIK3CA	c.2102A>C	p.H701P	SNV
PIK3CA	c.2702G>T	p.C901F	SNV
PIK3CA	c.2725T>C	p.F909L	SNV
PIK3CA	c.3110A>G	p.E1037G	SNV

Gene	Mutation CDS	Mutation AA	Mutation Type
PTEN	c.253+1G>A	p.?	SNV
PTEN	c.263A>G	p.Y88C	SNV
PTEN	c.302T>C	p.I101T	SNV
PTEN	c.314G>T	p.C105F	SNV
PTEN	c.334C>G	p.L112V	SNV
PTEN	c.395G>A	p.G132D	SNV
PTEN	c.449A>G	p.E150G	SNV
PTEN	c.464A>G	p.Y155C	SNV
PTEN	c.477G>T	p.R159S	SNV
PTEN	c.493-12delT	p.?	DEL
PTEN	c.578T>C	p.L193P	SNV
PTEN	c.595_597delATG	p.M199del	DEL
PTEN	c.610C>A	p.P204T	SNV
PTEN	c.615G>A	p.M205I	SNV
PTEN	c.697C>T	p.R233*	SNV
PTEN	c.703G>T	p.E235*	SNV
PTEN	c.721T>C	p.F241L	SNV
PTEN	c.787A>T	p.K263*	SNV
PTEN	c.800delA	p.K267fs*9	DEL
PTEN	c.879A>G	p.G293G	SNV
PTEN	c.895G>T	p.E299*	SNV
PTEN	c.955_958delACTT	p.T319fs*1	DEL
PTEN	c.1025A>G	p.K342R	SNV
PTEN	c.1026+32T>G	p.(=)	SNV
PTEN	c.1027-2A>G	p.?	SNV
PTEN	c.1040_1041delTC	p.F347fs*13	DEL
PTEN	c.1055A>G	p.E352G	SNV
PTEN	c.1091C>G	p.S364C	SNV
FGFR2	c.1647T>A	p.N549K	SNV
FGFR2	c.1144T>C	p.C382R	SNV
FGFR2	c.1124A>G	p.Y375C	SNV
FGFR2	c.1108A>G	p.T370A	SNV
FGFR2	c.929A>G	p.K310R	SNV
FGFR2	c.913G>A	p.G305R	SNV
FGFR2	c.755C>G	p.S252W	SNV
HRAS	c.182A>G	p.Q61R	SNV
HRAS	c.175G>A	p.A59T	SNV
HRAS	c.81T>C	p.H27H	SNV
HRAS	c.35G>T	p.G12V	SNV

Gene	Mutation CDS	Mutation AA	Mutation Type
PIK3CA	c.3140A>G	p.H1047R	SNV
PIK3CA	c.3204_3205insA	p.N1068fs*4	INS
FGFR3	c.746C>G	p.S249C	SNV
FGFR3	c.753C>T	p.H251H	SNV
FGFR3	c.850delC	p.H284fs*10	DEL
FGFR3	c.1108G>T	p.G370C	SNV
FGFR3	c.1138G>A	p.G380R	SNV
FGFR3	c.1150T>C	p.F384L	SNV
FGFR3	c.1172C>A	p.A391E	SNV
FGFR3	c.1928A>G	p.H643R	SNV
FGFR3	c.1948A>G	p.K650E	SNV
FGFR3	c.1959A>G	p.T651T	SNV
FGFR3	c.2089G>T	p.G697C	SNV
FGFR3	c.2401A>C	p.P800P	SNV
PDGFRA	c.1698_1712del15	p.S566_E571>R	DEL
PDGFRA	c.1701A>G	p.P567P	SNV
PDGFRA	c.1743T>C	p.P581P	SNV
PDGFRA	c.1977C>A	p.N659K	SNV
PDGFRA	c.2001A>G	p.S667S	SNV
PDGFRA	c.2021C>T	p.T674I	SNV
PDGFRA	c.2440-50_c.2440-49insA	p.(=)	INS
PDGFRA	c.2517G>T	p.L839L	SNV
PDGFRA	c.2525A>T	p.D842V	SNV
PDGFRA	c.2544C>A	p.N848K	SNV
KIT	c.92C>T	p.P31L	SNV
KIT	c.154G>A	p.D52N	SNV
KIT	c.218A>G	p.E73G	SNV
KIT	c.1405T>C	p.F469L	SNV
KIT	c.1416A>G	p.L472L	SNV
KIT	c.1509_1510insGCCTAT	p.Y503_F504insAY	INS
KIT	c.1516T>C	p.F506L	SNV
KIT	c.1526A>T	p.K509I	SNV
KIT	c.1535A>G	p.N512S	SNV
KIT	c.1588G>A	p.V530I	SNV
KIT	c.1621A>C	p.M541L	SNV
KIT	c.1698C>T	p.N566N	SNV
KIT	c.1727T>C	p.L576P	SNV

Gene	Mutation CDS	Mutation AA	Mutation Type
ATM	c.1009C>T	p.R337C	SNV
ATM	c.1229T>C	p.V410A	SNV
ATM	c.1810C>T	p.P604S	SNV
ATM	c.1898+2T>A	p.?	SNV
ATM	c.2572T>C	p.F858L	SNV
ATM	c.3925G>A	p.A1309T	SNV
ATM	c.5044G>T	p.D1682Y	SNV
ATM	c.5152C>G	p.L1718V	SNV
ATM	c.5178-1G>T	p.?	SNV
ATM	c.5188C>T	p.R1730*	SNV
ATM	c.5224G>C	p.A1742P	SNV
ATM	c.5380C>T	p.L1794L	SNV
ATM	c.5476T>G	p.L1826V	SNV
ATM	c.5821G>C	p.V1941L	SNV
ATM	c.7325A>C	p.Q2442P	SNV
ATM	c.7996A>G	p.T2666A	SNV
ATM	c.8084G>C	p.G2695A	SNV
ATM	c.8095C>A	p.P2699T	SNV
ATM	c.8174A>T	p.D2725V	SNV
ATM	c.8624A>G	p.N2875S	SNV
ATM	c.8668C>G	p.L2890V	SNV
ATM	c.8671+104T>C	p.(=)	SNV
ATM	c.8839A>T	p.T2947S	SNV
ATM	c.8850+60A>G	p.(=)	SNV
ATM	c.9023G>A	p.R3008H	SNV
ATM	c.9054A>G	p.K3018K	SNV
ATM	c.9139C>T	p.R3047*	SNV
KRAS	c.491G>A	p.R164Q	SNV
KRAS	c.351A>C	p.K117N	SNV
KRAS	c.183A>C	p.Q61H	SNV
KRAS	c.175G>A	p.A59T	SNV
KRAS	c.111+1C>T	p.?	SNV
KRAS	c.104C>T	p.T35I	SNV
KRAS	c.35G>A	p.G12D	SNV
KRAS	c.24A>G	p.V8V	SNV
PTPN11	c.181G>T	p.D61Y	SNV
PTPN11	c.205G>A	p.E69K	SNV

Gene	Mutation CDS	Mutation AA	Mutation Type
KIT	c.1755C>T	p.P585P	SNV
KIT	c.1924A>G	p.K642E	SNV
KIT	c.1961T>C	p.V654A	SNV
KIT	c.2089C>T	p.H697Y	SNV
KIT	c.2148T>C	p.D716D	SNV
KIT	c.2209G>A	p.D737N	SNV
KIT	c.2410C>T	p.R804W	SNV
KIT	c.2484+43T>A	p.?	SNV
KIT	c.2484+78T>C	p.(=)	SNV
KIT	c.2558G>A	p.W853*	SNV
KIT	c.2586G>C	p.L862L	SNV
KDR	c.*27T>C	p.(=)	SNV
KDR	c.4008C>T	p.T1336T	SNV
KDR	c.3594del25	p.V1199fs*27	DEL
KDR	c.3433G>A	p.G1145R	SNV
KDR	c.2917G>T	p.A973S	SNV
KDR	c.2619A>G	p.G873G	SNV
KDR	c.2615-37_2615-36insC	p.(=)	INS
KDR	c.1416A>T	p.Q472H	SNV
KDR	c.1413-42del41	p.?	DEL
KDR	c.824G>T	p.R275L	SNV
KDR	c.798+54G>A	p.(=)	SNV
FBXW7	c.2079A>G	p.E693E	SNV
FBXW7	c.2065C>T	p.R689W	SNV
FBXW7	c.2033C>G	p.S678*	SNV
FBXW7	c.2001delG	p.S668fs*39	DEL
FBXW7	c.1473_1474insCTCGTTGATTTCAGAGAATATGAATATGATC	p.N492fs*42	INS
FBXW7	c.1580A>G	p.D527G	SNV
FBXW7	c.1576T>C	p.W526R	SNV
FBXW7	c.1558G>A	p.D520N	SNV
FBXW7	c.1451G>T	p.R484M	SNV
FBXW7	c.1436G>A	p.R479Q	SNV
FBXW7	c.1394G>A	p.R465H	SNV
FBXW7	c.1338G>A	p.W446*	SNV

Gene	Mutation CDS	Mutation AA	Mutation Type
PTPN11	c.215C>T	p.A72V	SNV
PTPN11	c.226G>A	p.E76K	SNV
PTPN11	c.1472C>T	p.P491L	SNV
PTPN11	c.1508G>C	p.G503A	SNV
PTPN11	c.1519A>G	p.T507A	SNV
PTPN11	c.1528C>A	p.Q510K	SNV
HNF1A	c.617G>T	p.W206L	SNV
HNF1A	c.632A>C	p.Q211P	SNV
HNF1A	c.685C>T	p.R229*	SNV
HNF1A	c.710A>G	p.N237S	SNV
HNF1A	c.779C>T	p.T260M	SNV
HNF1A	c.787C>T	p.R263C	SNV
HNF1A	c.872_873insC	p.G292fs*25	INS
HNF1A	c.864G>C	p.G288G	SNV
FLT3	c.2516A>G	p.D839G	SNV
FLT3	c.2503G>T	p.D835Y	SNV
FLT3	c.2492G>A	p.G831E	SNV
FLT3	c.2039C>T	p.A680V	SNV
FLT3	c.1800_1801insTTCAGAGAATATGAATATGAT	p.D600_L601insFREYEYD	INS
FLT3	c.1775T>C	p.V592A	SNV
FLT3	c.1352C>T	p.S451F	SNV
FLT3	c.1310-3T>C	p.?	SNV
RB1	c.409G>T	p.E137*	SNV
RB1	c.596T>A	p.L199*	SNV
RB1	c.940-2A>T	p.?	SNV
RB1	c.958C>T	p.R320*	SNV
RB1	c.968A>G	p.E323G	SNV
RB1	c.982A>G	p.N328D	SNV
RB1	c.1072C>T	p.R358*	SNV
RB1	c.1363C>T	p.R455*	SNV
RB1	c.1654C>T	p.R552*	SNV
RB1	c.1666C>T	p.R556*	SNV
RB1	c.1687T>C	p.W563R	SNV
RB1	c.1735C>T	p.R579*	SNV

Gene	Mutation CDS	Mutation AA	Mutation Type
FBXW7	c.1322G>T	p.R441L	SNV
FBXW7	c.1177C>T	p.R393*	SNV
FBXW7	c.832C>T	p.R278*	SNV
FBXW7	c.744G>T	p.E248D	SNV
APC	c.2543_2544insA	p.D849fs*2	INS
APC	c.2626C>T	p.R876*	SNV
APC	c.2639T>C	p.I880T	SNV
APC	c.2656C>T	p.Q886*	SNV
APC	c.2752G>T	p.E918*	SNV
APC	c.3286C>T	p.Q1096*	SNV
APC	c.3305A>G	p.Y1102C	SNV
APC	c.3435A>G	p.E1145E	SNV
APC	c.3700delA	p.S1234fs*31	DEL
APC	c.3795A>G	p.E1265E	SNV
APC	c.3871C>T	p.Q1291*	SNV
APC	c.3880C>T	p.Q1294*	SNV
APC	c.3923_3924insA	p.E1309fs*6	INS
APC	c.3964G>T	p.E1322*	SNV
APC	c.4057G>T	p.E1353*	SNV
APC	c.4063T>C	p.S1355P	SNV
APC	c.4132C>T	p.Q1378*	SNV
APC	c.4141C>T	p.P1381S	SNV
APC	c.4189_4190delGA	p.R1399fs*9	DEL
APC	c.4216C>T	p.Q1406*	SNV
APC	c.4285C>T	p.Q1429*	SNV
APC	c.4393_4394delAG	p.S1465fs*3	DEL
APC	c.4540delC	p.P1514fs*9	DEL
APC	c.4561G>T	p.E1521*	SNV
APC	c.4639G>T	p.E1547*	SNV
APC	c.4654G>T	p.E1552*	SNV
APC	c.4666_4667insA	p.T1556fs*3	INS
APC	c.4773_4774insA	p.P1594fs*38	INS
APC	c.4826C>T	p.P1609L	SNV
CSF1R	c.*36A>C	p.(=)	SNV
CSF1R	c.*35C>T	p.(=)	SNV
CSF1R	c.2906A>G	p.Y969C	SNV
CSF1R	c.2878G>A	p.A960T	SNV

Gene	Mutation CDS	Mutation AA	Mutation Type
RB1	c.1814+2T>C	p.?	SNV
RB1	c.2028_2040del13	p.L676fs*16	DEL
RB1	c.2053C>T	p.Q685*	SNV
RB1	c.2063T>C	p.L688P	SNV
RB1	c.2107-2A>G	p.?	SNV
RB1	c.2117G>T	p.C706F	SNV
RB1	c.2143A>T	p.K715*	SNV
RB1	c.2153A>G	p.D718G	SNV
RB1	c.2242G>T	p.E748*	SNV
RB1	c.2261T>G	p.V754G	SNV
RB1	c.2267A>G	p.Y756C	SNV
RB1	c.2293A>T	p.K765*	SNV
AKT1	c.49G>A	p.E17K	SNV
MAP2K1	c.171G>T	p.K57N	SNV
MAP2K1	c.199G>A	p.D67N	SNV
IDH2	c.515G>A	p.R172K	SNV
IDH2	c.474A>G	p.P158P	SNV
IDH2	c.419G>A	p.R140Q	SNV
CDH1	c.241del30	p.G81_F91del	DEL
CDH1	c.1058A>G	p.E353G	SNV
CDH1	c.1108G>C	p.D370H	SNV
CDH1	c.1204G>A	p.D402N	SNV
CDH1	c.1733_1734insC	p.G579fs*9	INS
CDH1	c.1742T>C	p.L581P	SNV
CDH1	c.1774G>A	p.A592T	SNV
CDH1	c.1849G>A	p.A617T	SNV
CDH1	c.1901C>T	p.A634V	SNV
CDH1	c.1913G>A	p.W638*	SNV
TP53	c.1146delA	p.K382fs*>12	DEL
TP53	c.1123C>T	p.Q375*	SNV
TP53	c.1101-2A>G	p.?	SNV
TP53	c.1024C>T	p.R342*	SNV
TP53	c.1015G>T	p.E339*	SNV
TP53	c.1009C>T	p.R337C	SNV
TP53	c.1001G>T	p.G334V	SNV
TP53	c.991C>T	p.Q331*	SNV
TP53	c.981T>G	p.Y327*	SNV

Gene	Mutation CDS	Mutation AA	Mutation Type
CSF1R	c.890-2_c.890-1insTCAAGCAGT	p.?	INS
NPM1	c.863_864insTCTG	p.W288fs*12	INS
EGFR	c.323G>A	p.R108K	SNV
EGFR	c.340G>A	p.E114K	SNV
EGFR	c.408C>T	p.P136P	SNV
EGFR	c.866C>T	p.A289V	SNV
EGFR	c.874G>T	p.V292L	SNV
EGFR	c.1793G>T	p.G598V	SNV
EGFR	c.1859G>A	p.C620Y	SNV
EGFR	c.2063T>C	p.L688P	SNV
EGFR	c.2092G>A	p.A698T	SNV
EGFR	c.2156G>C	p.G719A	SNV
EGFR	c.2170G>A	p.G724S	SNV
EGFR	c.2184+19G>A	p.(=)	SNV
EGFR	c.2197C>T	p.P733S	SNV
EGFR	c.2203G>A	p.G735S	SNV
EGFR	c.2222C>T	p.P741L	SNV
EGFR	c.2235_2249del15	p.E746_A750delELREA	DEL
EGFR	c.2293G>A	p.V765M	SNV
EGFR	c.2361G>A	p.Q787Q	SNV
EGFR	c.2375T>C	p.L792P	SNV
EGFR	c.2429G>A	p.G810D	SNV
EGFR	c.2441T>C	p.L814P	SNV
EGFR	c.2485G>A	p.E829K	SNV
EGFR	c.2497T>G	p.L833V	SNV
EGFR	c.2504A>T	p.H835L	SNV
EGFR	c.2515G>A	p.A839T	SNV
EGFR	c.2573T>G	p.L858R	SNV
EGFR	c.2582T>A	p.L861Q	SNV
EGFR	c.2588G>A	p.G863D	SNV
EGFR	c.2612C>G	p.A871G	SNV
MET	c.504G>T	p.E168D	SNV
MET	c.1124A>G	p.N375S	SNV
MET	c.3082+1G>A	p.?	SNV
MET	c.3336T>C	p.H1112H	SNV
MET	c.3370C>G	p.H1124D	SNV
MET	c.3534G>C	p.M1178I	SNV
MET	c.3562C>T	p.R1188*	SNV

Gene	Mutation CDS	Mutation AA	Mutation Type
TP53	c.963A>G	p.K321K	SNV
TP53	c.949C>T	p.Q317*	SNV
TP53	c.916C>T	p.R306*	SNV
TP53	c.892G>T	p.E298*	SNV
TP53	c.833C>T	p.P278L	SNV
TP53	c.818G>A	p.R273H	SNV
TP53	c.743G>A	p.R248Q	SNV
TP53	c.733G>A	p.G245S	SNV
TP53	c.722C>T	p.S241F	SNV
TP53	c.701A>G	p.Y234C	SNV
TP53	c.672+62A>G	p.(=)	SNV
TP53	c.659A>G	p.Y220C	SNV
TP53	c.653T>A	p.V218E	SNV
TP53	c.646G>A	p.V216M	SNV
TP53	c.614A>G	p.Y205C	SNV
TP53	c.542G>A	p.R181H	SNV
TP53	c.488A>G	p.Y163C	SNV
TP53	c.481G>A	p.A161T	SNV
TP53	c.469G>T	p.V157F	SNV
TP53	c.404G>A	p.C135Y	SNV
TP53	c.395A>G	p.K132R	SNV
TP53	c.388C>G	p.L130V	SNV
TP53	c.380C>T	p.S127F	SNV
TP53	c.375+17G>A	p.?	SNV
TP53	c.375G>A	p.T125T	SNV
TP53	c.329G>T	p.R110L	SNV
TP53	c.319T>G	p.Y107D	SNV
TP53	c.273G>A	p.W91*	SNV
TP53	c.245C>T	p.P82L	SNV
TP53	c.215G>C	p.R72P	SNV
TP53	c.166G>T	p.E56*	SNV
TP53	c.151G>T	p.E51*	SNV
TP53	c.134T>C	p.L45P	SNV
TP53	c.112C>T	p.Q38*	SNV
TP53	c.80delC	p.P27fs*17	DEL
TP53	c.74+38C>G	p.(=)	SNV
ERBB2	c.2264T>C	p.L755S	SNV
ERBB2	c.2305G>T	p.D769Y	SNV

Gene	Mutation CDS	Mutation AA	Mutation Type
MET	c.3573T>C	p.T1191T	SNV
MET	c.3668T>G	p.L1223W	SNV
MET	c.3757T>G	p.Y1253D	SNV
MET	c.3778G>T	p.G1260C	SNV
MET	c.3785A>G	p.K1262R	SNV
MET	c.3803T>C	p.M1268T	SNV
MET	c.4071G>A	p.A1357A	SNV
MET	c.4146G>A	p.P1382P	SNV
SMO	c.595C>T	p.R199W	SNV
SMO	c.970G>A	p.A324T	SNV
SMO	c.1234C>T	p.L412F	SNV
SMO	c.1264+41A >G	p.(=)	SNV
SMO	c.1604G>T	p.W535L	SNV
SMO	c.1918A>G	p.T640A	SNV
BRAF	c.1799T>A	p.V600E	SNV
BRAF	c.1790T>G	p.L597R	SNV
BRAF	c.1781A>G	p.D594G	SNV
BRAF	c.1742A>G	p.N581S	SNV
BRAF	c.1391G>T	p.G464V	SNV
BRAF	c.1380A>G	p.G460G	SNV
BRAF	c.1359T>C	p.P453P	SNV
BRAF	c.1330C>T	p.R444W	SNV
EZH2	c.1937A>T	p.Y646F	SNV
FGFR1	c.816C>T	p.N272N	SNV
FGFR1	c.448C>T	p.P150S	SNV
FGFR1	c.421A>G	p.T141A	SNV
FGFR1	c.374C>T	p.S125L	SNV
JAK2	c.1849G>T	p.V617F	SNV
JAK2	c.1860C>A	p.D620E	SNV
CDKN2A	c.358G>T	p.E120*	SNV
CDKN2A	c.341C>T	p.P114L	SNV
CDKN2A	c.330G>A	p.W110*	SNV
CDKN2A	c.322G>T	p.D108Y	SNV
CDKN2A	c.247C>T	p.H83Y	SNV
CDKN2A	c.238C>T	p.R80*	SNV
CDKN2A	c.205G>T	p.E69*	SNV
CDKN2A	c.172C>T	p.R58*	SNV
GNAQ	c.1002C>T	p.T334T	SNV
GNAQ	c.829_830insGTAC	p.D277fs*20	INS

Gene	Mutation CDS	Mutation AA	Mutation Type
ERBB2	c.2324_2325ins12	p.A775_G776insYVMA	INS
ERBB2	c.2524G>A	p.V842I	SNV
ERBB2	c.2570A>G	p.N857S	SNV
ERBB2	c.2632C>T	p.H878Y	SNV
SMAD4	c.306T>C	p.P102P	SNV
SMAD4	c.377T>C	p.V126A	SNV
SMAD4	c.389C>T	p.P130L	SNV
SMAD4	c.403C>T	p.R135*	SNV
SMAD4	c.431C>G	p.S144*	SNV
SMAD4	c.502G>T	p.G168*	SNV
SMAD4	c.533C>A	p.S178*	SNV
SMAD4	c.547C>T	p.Q183*	SNV
SMAD4	c.733C>T	p.Q245*	SNV
SMAD4	c.766C>T	p.Q256*	SNV
SMAD4	c.776_777delCT	p.T259fs*4	DEL
SMAD4	c.931C>T	p.Q311*	SNV
SMAD4	c.955+5G>C	p.?	SNV
SMAD4	c.1001A>G	p.Q334R	SNV
SMAD4	c.1010A>G	p.E337G	SNV
SMAD4	c.1018A>G	p.K340E	SNV
SMAD4	c.1028C>G	p.S343*	SNV
SMAD4	c.1156G>C	p.G386R	SNV
SMAD4	c.1216G>A	p.A406T	SNV
SMAD4	c.1229_1230insCA	p.Q410fs*6	INS
SMAD4	c.1246A>G	p.R416G	SNV
SMAD4	c.1333C>T	p.R445*	SNV
SMAD4	c.1504A>G	p.R502G	SNV
SMAD4	c.1519A>G	p.K507E	SNV
SMAD4	c.1576G>T	p.E526*	SNV
SMAD4	c.1591C>A	p.R531R	SNV
STK11	c.169delG	p.E57fs*7	DEL
STK11	c.465-51T>C	p.(=)	SNV
STK11	c.465-1G>T	p.?	SNV
STK11	c.475C>T	p.Q159*	SNV
STK11	c.580G>T	p.D194Y	SNV
STK11	c.595G>T	p.E199*	SNV
STK11	c.816C>T	p.Y272Y	SNV
STK11	c.842C>T	p.P281L	SNV
STK11	c.1062C>G	p.F354L	SNV

Gene	Mutation CDS	Mutation AA	Mutation Type
GNAQ	c.736-34del12	p.?	DEL
GNAQ	c.735+34T>C	p.(=)	SNV
GNAQ	c.679_681TGT>GCA	p.M227_F228>SI	MNV
GNAQ	c.606-66del41	p.?	DEL
GNAQ	c.548G>A	p.R183Q	SNV
GNAQ	c.523A>T	p.T175S	SNV
ABL1	c.742C>G	p.L248V	SNV
ABL1	c.749G>A	p.G250E	SNV
ABL1	c.757T>C	p.Y253H	SNV
ABL1	c.763G>A	p.E255K	SNV
ABL1	c.827A>G	p.D276G	SNV
ABL1	c.878_879insGCC	p.I293>MP	INS
ABL1	c.1052T>C	p.M351T	SNV
ABL1	c.1064A>G	p.E355G	SNV
ABL1	c.1075T>G	p.F359V	SNV
ABL1	c.1150C>A	p.L384M	SNV
ABL1	c.1187A>G	p.H396R	SNV

Gene	Mutation CDS	Mutation AA	Mutation Type
GNA11	c.547C>T	p.R183C	SNV
GNA11	c.626A>T	p.Q209L	SNV
GNA11	c.771C>T	p.T257T	SNV
JAK3	c.2164G>A	p.V722I	SNV
JAK3	c.1715C>T	p.A572V	SNV
SRC	c.1460C>T	p.P487L	SNV
GNAS	c.489C>T	p.Y163Y	SNV
GNAS	c.601C>T	p.R201C	SNV
GNAS	c.680A>T	p.Q227L	SNV
SMARCB1	c.118C>T	p.R40*	SNV
SMARCB1	c.141C>A	p.Y47*	SNV
SMARCB1	c.157C>T	p.R53*	SNV
SMARCB1	c.472C>T	p.R158*	SNV
SMARCB1	c.566_567ins19	p.L191fs*26	INS
SMARCB1	c.601C>T	p.R201*	SNV
SMARCB1	c.607G>A	p.A203T	SNV
SMARCB1	c.601+66G>C	p.(=)	SNV
SMARCB1	c.1148delC	p.P383fs	DEL
