# Supplementary material for: Generic Protocols for the Analytical Validation of Next-Generation Sequencing-Based ctDNA Assays: A Joint Consensus Recommendation of the BloodPAC’s Analytical Variables Working Group
Source: Clin Chem. 2020 Sep 1;66(9):1156–66. doi: 10.1093/clinchem/hvaa164 (PMC7462123; doi:10.1093/clinchem/hvaa164)
Supplement: hvaa164_Supplementary_Data [file hvaa164_supplementary_data.zip › hvaa164-suppl_data/Supplemental Material 2.pdf]

Supplemental Materials 2

STANDARD METHOD FOR PREPARATION OF ACROMETRIX ONCOLOGY HOTSPOT CONTROL REFERENCE MATERIAL

Note: The methods in this section represent those currently practiced by Thermo Fisher Scientific for preparing cfDNA reference materials and has been included here as an example process used by a manufacturer of reference/control material. Assay developers who are preparing contrived materials in their labs for use in various analytical validation protocols should refer to the standard methods described above.

SNVs: The contrived sample is a mixture of AcroMetrix™ Oncology Hotspot Control material that contains 40-engineered variants covered by the Oncomine Lung cfDNA Assay, in a genomic DNA background of GM24385 from Coriell Institute for Medical Research. See Table 1 for a complete list of variants included in the control material. Currently, efforts are being made to commercialize this reference material.

Fragment AcroMetrix™ Oncology Hotspot Control material and GM24385 gDNA by sonication to mimic the size of cfDNA (165 to 175 bp). It is recommended to use a Bioanalyzer or similar fragment analysis method to demonstrate that the DNA fragmentation profile of any prepared contrived sample is comparable to the DNA size profile of cfDNA derived from plasma samples.

Serially dilute into two sample formats, one where the pooled variants are at 0.1% MAF and one where the pooled variants are at 0.5% MAF.

Fusions: Configure the assay for detection of *ALK, RET,* and *ROS* driver genes. Titrate total RNA (~1%) extracted from fusion positive cell lines into a cell free nucleic acid background (cfDNA and cfRNA) extracted from healthy donor plasma samples.

CNVs: Target configurations can include *CCND1, EGFR, MET, ERBB2*, *MYC* and *FGFR1* amplifications. Titrate cfDNA extracted from cell free medium of cultured cells harboring CNV targets and into a background of cfDNA extracted from healthy donor plasma samples.

Table 1 Targets Included in AcroMetrix® Oncology Hotspot Control

Gene	Mutation CDS	Mutation AA	Mutation Type
MPL	c.1514G>A	p.S505N	SNV
MPL	c.1544G>T	p.W515L	SNV
MPL	c.1555G>A	p.A519T	SNV
NRAS	c.182A>G	p.Q61R	SNV
NRAS	c.174A>G	p.T58T	SNV
NRAS	c.112-70C>T	p.(=)	SNV
NRAS	c.52G>A	p.A18T	SNV
NRAS	c.35G>A	p.G12D	SNV
NRAS	c.29G>A	p.G10E	SNV
ALK	c.3824G>A	p.R1275Q	SNV
NOTCH1	c.7412C>A	p.S2471*	SNV
NOTCH1	c.7386delC	p.A2463fs*14	DEL
NOTCH1	c.7375C>T	p.Q2459*	SNV
NOTCH1	c.7318C>T	p.Q2440*	SNV
NOTCH1	c.5033T>C	p.L1678P	SNV
NOTCH1	c.5025C>T	p.I1675I	SNV
NOTCH1	c.4799T>C	p.L1600P	SNV
NOTCH1	c.4793G>C	p.R1598P	SNV
NOTCH1	c.4778T>C	p.L1593P	SNV
NOTCH1	c.4721T>C	p.L1574P	SNV

Gene	Mutation CDS	Mutation AA	Mutation Type
ALK	c.3522C>A	p.F1174L	SNV
MSH6	c.3246G>T	p.P1082P	SNV
MSH6	c.3261delC	p.F1088fs*2	DEL
MSH6	c.3300G>A	p.T1100T	SNV
MSH6	c.3438+14A>T	p.(=)	SNV
IDH1	c.395G>A	p.R132H	SNV
IDH1	c.388A>G	p.I130V	SNV
IDH1	c.367G>A	p.G123R	SNV
ERBB4	c.2791G>T	p.D931Y	SNV
ERBB4	c.2782G>T	p.E928*	SNV
ERBB4	c.1835G>A	p.R612Q	SNV
ERBB4	c.1828C>A	p.P610T	SNV
ERBB4	c.1784A>G	p.D595G	SNV
ERBB4	c.1089T>C	p.N363N	SNV
ERBB4	c.1022C>T	p.S341L	SNV
ERBB4	c.1003G>T	p.D335Y	SNV
ERBB4	c.909T>C	p.S303S	SNV
ERBB4	c.885T>G	p.H295Q	SNV
ERBB4	c.829C>A	p.H277N	SNV
ERBB4	c.804C>A	p.Y268*	SNV
ERBB4	c.730A>G	p.T244A	SNV
ERBB4	c.704C>T	p.A235V	SNV
ERBB4	c.633G>A	p.T211T	SNV
ERBB4	c.542A>G	p.N181S	SNV
ERBB4	c.515C>G	p.P172R	SNV
VHL	c.266T>A	p.L89H	SNV
VHL	c.277G>C	p.G93R	SNV
VHL	c.286C>T	p.Q96*	SNV
VHL	c.296delC	p.P99fs*60	DEL
VHL	c.343C>A	p.H115N	SNV
VHL	c.353T>C	p.L118P	SNV
VHL	c.388G>C	p.V130L	SNV
VHL	c.431delG	p.G144fs*15	DEL
VHL	c.472C>G	p.L158V	SNV
VHL	c.481C>T	p.R161*	SNV
VHL	c.499C>T	p.R167W	SNV
VHL	c.506T>C	p.L169P	SNV
MLH1	c.1151T>A	p.V384D	SNV

Gene	Mutation CDS	Mutation AA	Mutation Type
RET	c.1852T>C	p.C618R	SNV
RET	c.1858T>C	p.C620R	SNV
RET	c.1894_1906>AGCT	p.E632_T636>SS	Complex
RET	c.1942G>A	p.V648I	SNV
RET	c.1991C>A	p.A664D	SNV
RET	c.2304G>C	p.E768D	SNV
RET	c.2307T>A	p.L769L	SNV
RET	c.2647_2648GC>TT	p.A883F	MNV
RET	c.2701G>A	p.E901K	SNV
RET	c.2753T>C	p.M918T	SNV
PTEN	c.19G>T	p.E7*	SNV
PTEN	c.40A>G	p.R14G	SNV
PTEN	c.49C>T	p.Q17*	SNV
PTEN	c.71A>G	p.D24G	SNV
PTEN	c.80-96A>G	p.(=)	SNV
PTEN	c.80A>G	p.Y27C	SNV
PTEN	c.112C>T	p.P38S	SNV
PTEN	c.142A>G	p.N48D	SNV
PTEN	c.156T>C	p.D52D	SNV
PTEN	c.163A>G	p.R55G	SNV
PTEN	c.166T>G	p.F56V	SNV
PTEN	c.202T>C	p.Y68H	SNV
PTEN	c.209+5G>A	p.?	SNV
PTEN	c.212G>A	p.C71Y	SNV
PTEN	c.227_228delAT	p.Y76fs*1	DEL
PTEN	c.245A>C	p.N82T	SNV
PTEN	c.253+1G>A	p.?	SNV
PTEN	c.263A>G	p.Y88C	SNV
PTEN	c.302T>C	p.I101T	SNV
PTEN	c.314G>T	p.C105F	SNV
PTEN	c.334C>G	p.L112V	SNV
PTEN	c.395G>A	p.G132D	SNV
PTEN	c.449A>G	p.E150G	SNV
PTEN	c.464A>G	p.Y155C	SNV
PTEN	c.477G>T	p.R159S	SNV
PTEN	c.493-12delT	p.?	DEL
PTEN	c.578T>C	p.L193P	SNV
PTEN	c.595_597delATG	p.M199del	DEL

Gene	Mutation CDS	Mutation AA	Mutation Type
CTNNB1	c.98C>G	p.S33C	SNV
CTNNB1	c.110C>T	p.S37F	SNV
CTNNB1	c.121A>G	p.T41A	SNV
CTNNB1	c.134C>T	p.S45F	SNV
FOXL2	c.402C>G	p.C134W	SNV
PIK3CA	c.35G>A	p.G12D	SNV
PIK3CA	c.93A>G	p.I31M	SNV
PIK3CA	c.180A>G	p.Q60Q	SNV
PIK3CA	c.210C>T	p.F70F	SNV
PIK3CA	c.323G>A	p.R108H	SNV
PIK3CA	c.331A>G	p.K111E	SNV
PIK3CA	c.344G>T	p.R115L	SNV
PIK3CA	c.536A>G	p.K179R	SNV
PIK3CA	c.971C>T	p.T324I	SNV
PIK3CA	c.1002C>T	p.L334L	SNV
PIK3CA	c.1035T>A	p.N345K	SNV
PIK3CA	c.1213T>C	p.S405P	SNV
PIK3CA	c.1258T>C	p.C420R	SNV
PIK3CA	c.1370A>G	p.N457S	SNV
PIK3CA	c.1616C>G	p.P539R	SNV
PIK3CA	c.1624G>A	p.E542K	SNV
PIK3CA	c.1633G>A	p.E545K	SNV
PIK3CA	c.1640A>G	p.E547G	SNV
PIK3CA	c.2102A>C	p.H701P	SNV
PIK3CA	c.2702G>T	p.C901F	SNV
PIK3CA	c.2725T>C	p.F909L	SNV
PIK3CA	c.3110A>G	p.E1037G	SNV
PIK3CA	c.3140A>G	p.H1047R	SNV
PIK3CA	c.3204_3205insA	p.N1068fs*4	INS
FGFR3	c.746C>G	p.S249C	SNV
FGFR3	c.753C>T	p.H251H	SNV
FGFR3	c.850delC	p.H284fs*10	DEL
FGFR3	c.1108G>T	p.G370C	SNV
FGFR3	c.1138G>A	p.G380R	SNV
FGFR3	c.1150T>C	p.F384L	SNV
FGFR3	c.1172C>A	p.A391E	SNV
FGFR3	c.1928A>G	p.H643R	SNV

Gene	Mutation CDS	Mutation AA	Mutation Type
PTEN	c.610C>A	p.P204T	SNV
PTEN	c.615G>A	p.M205I	SNV
PTEN	c.697C>T	p.R233*	SNV
PTEN	c.703G>T	p.E235*	SNV
PTEN	c.721T>C	p.F241L	SNV
PTEN	c.787A>T	p.K263*	SNV
PTEN	c.800delA	p.K267fs*9	DEL
PTEN	c.879A>G	p.G293G	SNV
PTEN	c.895G>T	p.E299*	SNV
PTEN	c.955_958delACTT	p.T319fs*1	DEL
PTEN	c.1025A>G	p.K342R	SNV
PTEN	c.1026+32T>G	p.(=)	SNV
PTEN	c.1027-2A>G	p.?	SNV
PTEN	c.1040_1041delTC	p.F347fs*13	DEL
PTEN	c.1055A>G	p.E352G	SNV
PTEN	c.1091C>G	p.S364C	SNV
FGFR2	c.1647T>A	p.N549K	SNV
FGFR2	c.1144T>C	p.C382R	SNV
FGFR2	c.1124A>G	p.Y375C	SNV
FGFR2	c.1108A>G	p.T370A	SNV
FGFR2	c.929A>G	p.K310R	SNV
FGFR2	c.913G>A	p.G305R	SNV
FGFR2	c.755C>G	p.S252W	SNV
HRAS	c.182A>G	p.Q61R	SNV
HRAS	c.175G>A	p.A59T	SNV
HRAS	c.81T>C	p.H27H	SNV
HRAS	c.35G>T	p.G12V	SNV
ATM	c.1009C>T	p.R337C	SNV
ATM	c.1229T>C	p.V410A	SNV
ATM	c.1810C>T	p.P604S	SNV
ATM	c.1898+2T>A	p.?	SNV
ATM	c.2572T>C	p.F858L	SNV
ATM	c.3925G>A	p.A1309T	SNV
ATM	c.5044G>T	p.D1682Y	SNV
ATM	c.5152C>G	p.L1718V	SNV
ATM	c.5178-1G>T	p.?	SNV
ATM	c.5188C>T	p.R1730*	SNV

Gene	Mutation CDS	Mutation AA	Mutation Type
FGFR3	c.1948A>G	p.K650E	SNV
FGFR3	c.1959A>G	p.T651T	SNV
FGFR3	c.2089G>T	p.G697C	SNV
FGFR3	c.2401A>C	p.P800P	SNV
PDGFRA	c.1698_1712del15	p.S566_E571>R	DEL
PDGFRA	c.1701A>G	p.P567P	SNV
PDGFRA	c.1743T>C	p.P581P	SNV
PDGFRA	c.1977C>A	p.N659K	SNV
PDGFRA	c.2001A>G	p.S667S	SNV
PDGFRA	c.2021C>T	p.T674I	SNV
PDGFRA	c.2440-50_c.2440-49insA	p.(=)	INS
PDGFRA	c.2517G>T	p.L839L	SNV
PDGFRA	c.2525A>T	p.D842V	SNV
PDGFRA	c.2544C>A	p.N848K	SNV
KIT	c.92C>T	p.P31L	SNV
KIT	c.154G>A	p.D52N	SNV
KIT	c.218A>G	p.E73G	SNV
KIT	c.1405T>C	p.F469L	SNV
KIT	c.1416A>G	p.L472L	SNV
KIT	c.1509_1510insGCCTAT	p.Y503_F504insAY	INS
KIT	c.1516T>C	p.F506L	SNV
KIT	c.1526A>T	p.K509I	SNV
KIT	c.1535A>G	p.N512S	SNV
KIT	c.1588G>A	p.V530I	SNV
KIT	c.1621A>C	p.M541L	SNV
KIT	c.1698C>T	p.N566N	SNV
KIT	c.1727T>C	p.L576P	SNV
KIT	c.1755C>T	p.P585P	SNV
KIT	c.1924A>G	p.K642E	SNV
KIT	c.1961T>C	p.V654A	SNV
KIT	c.2089C>T	p.H697Y	SNV
KIT	c.2148T>C	p.D716D	SNV
KIT	c.2209G>A	p.D737N	SNV
KIT	c.2410C>T	p.R804W	SNV
KIT	c.2484+43T>A	p.?	SNV
KIT	c.2484+78T>C	p.(=)	SNV

Gene	Mutation CDS	Mutation AA	Mutation Type
ATM	c.5224G>C	p.A1742P	SNV
ATM	c.5380C>T	p.L1794L	SNV
ATM	c.5476T>G	p.L1826V	SNV
ATM	c.5821G>C	p.V1941L	SNV
ATM	c.7325A>C	p.Q2442P	SNV
ATM	c.7996A>G	p.T2666A	SNV
ATM	c.8084G>C	p.G2695A	SNV
ATM	c.8095C>A	p.P2699T	SNV
ATM	c.8174A>T	p.D2725V	SNV
ATM	c.8624A>G	p.N2875S	SNV
ATM	c.8668C>G	p.L2890V	SNV
ATM	c.8671+104T>C	p.(=)	SNV
ATM	c.8839A>T	p.T2947S	SNV
ATM	c.8850+60A>G	p.(=)	SNV
ATM	c.9023G>A	p.R3008H	SNV
ATM	c.9054A>G	p.K3018K	SNV
ATM	c.9139C>T	p.R3047*	SNV
KRAS	c.491G>A	p.R164Q	SNV
KRAS	c.351A>C	p.K117N	SNV
KRAS	c.183A>C	p.Q61H	SNV
KRAS	c.175G>A	p.A59T	SNV
KRAS	c.111+1C>T	p.?	SNV
KRAS	c.104C>T	p.T35I	SNV
KRAS	c.35G>A	p.G12D	SNV
KRAS	c.24A>G	p.V8V	SNV
PTPN11	c.181G>T	p.D61Y	SNV
PTPN11	c.205G>A	p.E69K	SNV
PTPN11	c.215C>T	p.A72V	SNV
PTPN11	c.226G>A	p.E76K	SNV
PTPN11	c.1472C>T	p.P491L	SNV
PTPN11	c.1508G>C	p.G503A	SNV
PTPN11	c.1519A>G	p.T507A	SNV
PTPN11	c.1528C>A	p.Q510K	SNV
HNF1A	c.617G>T	p.W206L	SNV
HNF1A	c.632A>C	p.Q211P	SNV
HNF1A	c.685C>T	p.R229*	SNV

Gene	Mutation CDS	Mutation AA	Mutation Type
KIT	c.2558G>A	p.W853*	SNV
KIT	c.2586G>C	p.L862L	SNV
KDR	c.*27T>C	p.(=)	SNV
KDR	c.4008C>T	p.T1336T	SNV
KDR	c.3594del25	p.V1199fs*27	DEL
KDR	c.3433G>A	p.G1145R	SNV
KDR	c.2917G>T	p.A973S	SNV
KDR	c.2619A>G	p.G873G	SNV
KDR	c.2615-37_2615-36insC	p.(=)	INS
KDR	c.1416A>T	p.Q472H	SNV
KDR	c.1413-42del41	p.?	DEL
KDR	c.824G>T	p.R275L	SNV
KDR	c.798+54G>A	p.(=)	SNV
FBXW7	c.2079A>G	p.E693E	SNV
FBXW7	c.2065C>T	p.R689W	SNV
FBXW7	c.2033C>G	p.S678*	SNV
FBXW7	c.2001delG	p.S668fs*39	DEL
FBXW7	c.1473_1474insCTCGTTGATTTCAGAGAATATGAATATGATC	p.N492fs*42	INS
FBXW7	c.1580A>G	p.D527G	SNV
FBXW7	c.1576T>C	p.W526R	SNV
FBXW7	c.1558G>A	p.D520N	SNV
FBXW7	c.1451G>T	p.R484M	SNV
FBXW7	c.1436G>A	p.R479Q	SNV
FBXW7	c.1394G>A	p.R465H	SNV
FBXW7	c.1338G>A	p.W446*	SNV
FBXW7	c.1322G>T	p.R441L	SNV
FBXW7	c.1177C>T	p.R393*	SNV
FBXW7	c.832C>T	p.R278*	SNV
FBXW7	c.744G>T	p.E248D	SNV
APC	c.2543_2544insA	p.D849fs*2	INS
APC	c.2626C>T	p.R876*	SNV
APC	c.2639T>C	p.I880T	SNV

Gene	Mutation CDS	Mutation AA	Mutation Type
HNF1A	c.710A>G	p.N237S	SNV
HNF1A	c.779C>T	p.T260M	SNV
HNF1A	c.787C>T	p.R263C	SNV
HNF1A	c.872_873insC	p.G292fs*25	INS
HNF1A	c.864G>C	p.G288G	SNV
FLT3	c.2516A>G	p.D839G	SNV
FLT3	c.2503G>T	p.D835Y	SNV
FLT3	c.2492G>A	p.G831E	SNV
FLT3	c.2039C>T	p.A680V	SNV
FLT3	c.1800_1801insTTCAGAGAATATGAATATGAT	p.D600_L601insFREYEYD	INS
FLT3	c.1775T>C	p.V592A	SNV
FLT3	c.1352C>T	p.S451F	SNV
FLT3	c.1310-3T>C	p.?	SNV
RB1	c.409G>T	p.E137*	SNV
RB1	c.596T>A	p.L199*	SNV
RB1	c.940-2A>T	p.?	SNV
RB1	c.958C>T	p.R320*	SNV
RB1	c.968A>G	p.E323G	SNV
RB1	c.982A>G	p.N328D	SNV
RB1	c.1072C>T	p.R358*	SNV
RB1	c.1363C>T	p.R455*	SNV
RB1	c.1654C>T	p.R552*	SNV
RB1	c.1666C>T	p.R556*	SNV
RB1	c.1687T>C	p.W563R	SNV
RB1	c.1735C>T	p.R579*	SNV
RB1	c.1814+2T>C	p.?	SNV
RB1	c.2028_2040del13	p.L676fs*16	DEL
RB1	c.2053C>T	p.Q685*	SNV
RB1	c.2063T>C	p.L688P	SNV
RB1	c.2107-2A>G	p.?	SNV
RB1	c.2117G>T	p.C706F	SNV
RB1	c.2143A>T	p.K715*	SNV

Gene	Mutation CDS	Mutation AA	Mutation Type
APC	c.2656C>T	p.Q886*	SNV
APC	c.2752G>T	p.E918*	SNV
APC	c.3286C>T	p.Q1096*	SNV
APC	c.3305A>G	p.Y1102C	SNV
APC	c.3435A>G	p.E1145E	SNV
APC	c.3700delA	p.S1234fs*31	DEL
APC	c.3795A>G	p.E1265E	SNV
APC	c.3871C>T	p.Q1291*	SNV
APC	c.3880C>T	p.Q1294*	SNV
APC	c.3923_3924insA	p.E1309fs*6	INS
APC	c.3964G>T	p.E1322*	SNV
APC	c.4057G>T	p.E1353*	SNV
APC	c.4063T>C	p.S1355P	SNV
APC	c.4132C>T	p.Q1378*	SNV
APC	c.4141C>T	p.P1381S	SNV
APC	c.4189_4190delGA	p.R1399fs*9	DEL
APC	c.4216C>T	p.Q1406*	SNV
APC	c.4285C>T	p.Q1429*	SNV
APC	c.4393_4394delAG	p.S1465fs*3	DEL
APC	c.4540delC	p.P1514fs*9	DEL
APC	c.4561G>T	p.E1521*	SNV
APC	c.4639G>T	p.E1547*	SNV
APC	c.4654G>T	p.E1552*	SNV
APC	c.4666_4667insA	p.T1556fs*3	INS
APC	c.4773_4774insA	p.P1594fs*38	INS
APC	c.4826C>T	p.P1609L	SNV
CSF1R	c.*36A>C	p.(=)	SNV
CSF1R	c.*35C>T	p.(=)	SNV
CSF1R	c.2906A>G	p.Y969C	SNV
CSF1R	c.2878G>A	p.A960T	SNV
CSF1R	c.890-2_c.890-1insTCAAGCAGT	p.?	INS
NPM1	c.863_864insTCTG	p.W288fs*12	INS
EGFR	c.323G>A	p.R108K	SNV
EGFR	c.340G>A	p.E114K	SNV
EGFR	c.408C>T	p.P136P	SNV

Gene	Mutation CDS	Mutation AA	Mutation Type
RB1	c.2153A>G	p.D718G	SNV
RB1	c.2242G>T	p.E748*	SNV
RB1	c.2261T>G	p.V754G	SNV
RB1	c.2267A>G	p.Y756C	SNV
RB1	c.2293A>T	p.K765*	SNV
AKT1	c.49G>A	p.E17K	SNV
MAP2K1	c.171G>T	p.K57N	SNV
MAP2K1	c.199G>A	p.D67N	SNV
IDH2	c.515G>A	p.R172K	SNV
IDH2	c.474A>G	p.P158P	SNV
IDH2	c.419G>A	p.R140Q	SNV
CDH1	c.241del30	p.G81_F91del	DEL
CDH1	c.1058A>G	p.E353G	SNV
CDH1	c.1108G>C	p.D370H	SNV
CDH1	c.1204G>A	p.D402N	SNV
CDH1	c.1733_1734insC	p.G579fs*9	INS
CDH1	c.1742T>C	p.L581P	SNV
CDH1	c.1774G>A	p.A592T	SNV
CDH1	c.1849G>A	p.A617T	SNV
CDH1	c.1901C>T	p.A634V	SNV
CDH1	c.1913G>A	p.W638*	SNV
TP53	c.1146delA	p.K382fs*>12	DEL
TP53	c.1123C>T	p.Q375*	SNV
TP53	c.1101-2A>G	p.?	SNV
TP53	c.1024C>T	p.R342*	SNV
TP53	c.1015G>T	p.E339*	SNV
TP53	c.1009C>T	p.R337C	SNV
TP53	c.1001G>T	p.G334V	SNV
TP53	c.991C>T	p.Q331*	SNV
TP53	c.981T>G	p.Y327*	SNV
TP53	c.963A>G	p.K321K	SNV
TP53	c.949C>T	p.Q317*	SNV
TP53	c.916C>T	p.R306*	SNV
TP53	c.892G>T	p.E298*	SNV
TP53	c.833C>T	p.P278L	SNV

Gene	Mutation CDS	Mutation AA	Mutation Type
EGFR	c.866C>T	p.A289V	SNV
EGFR	c.874G>T	p.V292L	SNV
EGFR	c.1793G>T	p.G598V	SNV
EGFR	c.1859G>A	p.C620Y	SNV
EGFR	c.2063T>C	p.L688P	SNV
EGFR	c.2092G>A	p.A698T	SNV
EGFR	c.2156G>C	p.G719A	SNV
EGFR	c.2170G>A	p.G724S	SNV
EGFR	c.2184+19G>A	p.(=)	SNV
EGFR	c.2197C>T	p.P733S	SNV
EGFR	c.2203G>A	p.G735S	SNV
EGFR	c.2222C>T	p.P741L	SNV
EGFR	c.2235_2249del15	p.E746_A750delELREA	DEL
EGFR	c.2293G>A	p.V765M	SNV
EGFR	c.2361G>A	p.Q787Q	SNV
EGFR	c.2375T>C	p.L792P	SNV
EGFR	c.2429G>A	p.G810D	SNV
EGFR	c.2441T>C	p.L814P	SNV
EGFR	c.2485G>A	p.E829K	SNV
EGFR	c.2497T>G	p.L833V	SNV
EGFR	c.2504A>T	p.H835L	SNV
EGFR	c.2515G>A	p.A839T	SNV
EGFR	c.2573T>G	p.L858R	SNV
EGFR	c.2582T>A	p.L861Q	SNV
EGFR	c.2588G>A	p.G863D	SNV
EGFR	c.2612C>G	p.A871G	SNV
MET	c.504G>T	p.E168D	SNV
MET	c.1124A>G	p.N375S	SNV
MET	c.3082+1G>A	p.?	SNV
MET	c.3336T>C	p.H1112H	SNV
MET	c.3370C>G	p.H1124D	SNV
MET	c.3534G>C	p.M1178I	SNV
MET	c.3562C>T	p.R1188*	SNV
MET	c.3573T>C	p.T1191T	SNV
MET	c.3668T>G	p.L1223W	SNV
MET	c.3757T>G	p.Y1253D	SNV
MET	c.3778G>T	p.G1260C	SNV
MET	c.3785A>G	p.K1262R	SNV

Gene	Mutation CDS	Mutation AA	Mutation Type
TP53	c.818G>A	p.R273H	SNV
TP53	c.743G>A	p.R248Q	SNV
TP53	c.733G>A	p.G245S	SNV
TP53	c.722C>T	p.S241F	SNV
TP53	c.701A>G	p.Y234C	SNV
TP53	c.672+62A>G	p.(=)	SNV
TP53	c.659A>G	p.Y220C	SNV
TP53	c.653T>A	p.V218E	SNV
TP53	c.646G>A	p.V216M	SNV
TP53	c.614A>G	p.Y205C	SNV
TP53	c.542G>A	p.R181H	SNV
TP53	c.488A>G	p.Y163C	SNV
TP53	c.481G>A	p.A161T	SNV
TP53	c.469G>T	p.V157F	SNV
TP53	c.404G>A	p.C135Y	SNV
TP53	c.395A>G	p.K132R	SNV
TP53	c.388C>G	p.L130V	SNV
TP53	c.380C>T	p.S127F	SNV
TP53	c.375+17G>A	p.?	SNV
TP53	c.375G>A	p.T125T	SNV
TP53	c.329G>T	p.R110L	SNV
TP53	c.319T>G	p.Y107D	SNV
TP53	c.273G>A	p.W91*	SNV
TP53	c.245C>T	p.P82L	SNV
TP53	c.215G>C	p.R72P	SNV
TP53	c.166G>T	p.E56*	SNV
TP53	c.151G>T	p.E51*	SNV
TP53	c.134T>C	p.L45P	SNV
TP53	c.112C>T	p.Q38*	SNV
TP53	c.80delC	p.P27fs*17	DEL
TP53	c.74+38C>G	p.(=)	SNV
ERBB2	c.2264T>C	p.L755S	SNV
ERBB2	c.2305G>T	p.D769Y	SNV
ERBB2	c.2324_2325ins12	p.A775_G776insYVMA	INS
ERBB2	c.2524G>A	p.V842I	SNV
ERBB2	c.2570A>G	p.N857S	SNV
ERBB2	c.2632C>T	p.H878Y	SNV
SMAD4	c.306T>C	p.P102P	SNV

Gene	Mutation CDS	Mutation AA	Mutation Type
MET	c.3803T>C	p.M1268T	SNV
MET	c.4071G>A	p.A1357A	SNV
MET	c.4146G>A	p.P1382P	SNV
SMO	c.595C>T	p.R199W	SNV
SMO	c.970G>A	p.A324T	SNV
SMO	c.1234C>T	p.L412F	SNV
SMO	c.1264+41A>G	p.(=)	SNV
SMO	c.1604G>T	p.W535L	SNV
SMO	c.1918A>G	p.T640A	SNV
BRAF	c.1799T>A	p.V600E	SNV
BRAF	c.1790T>G	p.L597R	SNV
BRAF	c.1781A>G	p.D594G	SNV
BRAF	c.1742A>G	p.N581S	SNV
BRAF	c.1391G>T	p.G464V	SNV
BRAF	c.1380A>G	p.G460G	SNV
BRAF	c.1359T>C	p.P453P	SNV
BRAF	c.1330C>T	p.R444W	SNV
EZH2	c.1937A>T	p.Y646F	SNV
FGFR1	c.816C>T	p.N272N	SNV
FGFR1	c.448C>T	p.P150S	SNV
FGFR1	c.421A>G	p.T141A	SNV
FGFR1	c.374C>T	p.S125L	SNV
JAK2	c.1849G>T	p.V617F	SNV
JAK2	c.1860C>A	p.D620E	SNV
CDKN2A	c.358G>T	p.E120*	SNV
CDKN2A	c.341C>T	p.P114L	SNV
CDKN2A	c.330G>A	p.W110*	SNV
CDKN2A	c.322G>T	p.D108Y	SNV
CDKN2A	c.247C>T	p.H83Y	SNV
CDKN2A	c.238C>T	p.R80*	SNV
CDKN2A	c.205G>T	p.E69*	SNV
CDKN2A	c.172C>T	p.R58*	SNV
GNAQ	c.1002C>T	p.T334T	SNV
GNAQ	c.829_830insGTAC	p.D277fs*20	INS
GNAQ	c.736-34del12	p.?	DEL
GNAQ	c.735+34T>C	p.(=)	SNV
GNAQ	c.679_681TGT>GCA	p.M227_F228>SI	MNV

Gene	Mutation CDS	Mutation AA	Mutation Type
SMAD4	c.377T>C	p.V126A	SNV
SMAD4	c.389C>T	p.P130L	SNV
SMAD4	c.403C>T	p.R135*	SNV
SMAD4	c.431C>G	p.S144*	SNV
SMAD4	c.502G>T	p.G168*	SNV
SMAD4	c.533C>A	p.S178*	SNV
SMAD4	c.547C>T	p.Q183*	SNV
SMAD4	c.733C>T	p.Q245*	SNV
SMAD4	c.766C>T	p.Q256*	SNV
SMAD4	c.776_777delCT	p.T259fs*4	DEL
SMAD4	c.931C>T	p.Q311*	SNV
SMAD4	c.955+5G>C	p.?	SNV
SMAD4	c.1001A>G	p.Q334R	SNV
SMAD4	c.1010A>G	p.E337G	SNV
SMAD4	c.1018A>G	p.K340E	SNV
SMAD4	c.1028C>G	p.S343*	SNV
SMAD4	c.1156G>C	p.G386R	SNV
SMAD4	c.1216G>A	p.A406T	SNV
SMAD4	c.1229_1230insCA	p.Q410fs*6	INS
SMAD4	c.1246A>G	p.R416G	SNV
SMAD4	c.1333C>T	p.R445*	SNV
SMAD4	c.1504A>G	p.R502G	SNV
SMAD4	c.1519A>G	p.K507E	SNV
SMAD4	c.1576G>T	p.E526*	SNV
SMAD4	c.1591C>A	p.R531R	SNV
STK11	c.169delG	p.E57fs*7	DEL
STK11	c.465-51T>C	p.(=)	SNV
STK11	c.465-1G>T	p.?	SNV
STK11	c.475C>T	p.Q159*	SNV
STK11	c.580G>T	p.D194Y	SNV
STK11	c.595G>T	p.E199*	SNV
STK11	c.816C>T	p.Y272Y	SNV
STK11	c.842C>T	p.P281L	SNV
STK11	c.1062C>G	p.F354L	SNV
GNA11	c.547C>T	p.R183C	SNV
GNA11	c.626A>T	p.Q209L	SNV
GNA11	c.771C>T	p.T257T	SNV

Gene	Mutation CDS	Mutation AA	Mutation Type
GNAQ	c.606-66del41	p.?	DEL
GNAQ	c.548G>A	p.R183Q	SNV
GNAQ	c.523A>T	p.T175S	SNV
ABL1	c.742C>G	p.L248V	SNV
ABL1	c.749G>A	p.G250E	SNV
ABL1	c.757T>C	p.Y253H	SNV
ABL1	c.763G>A	p.E255K	SNV
ABL1	c.827A>G	p.D276G	SNV
ABL1	c.878_879insGCC	p.I293>MP	INS
ABL1	c.1052T>C	p.M351T	SNV
ABL1	c.1064A>G	p.E355G	SNV
ABL1	c.1075T>G	p.F359V	SNV
ABL1	c.1150C>A	p.L384M	SNV
ABL1	c.1187A>G	p.H396R	SNV

Gene	Mutation CDS	Mutation AA	Mutation Type
JAK3	c.2164G>A	p.V722I	SNV
JAK3	c.1715C>T	p.A572V	SNV
SRC	c.1460C>T	p.P487L	SNV
GNAS	c.489C>T	p.Y163Y	SNV
GNAS	c.601C>T	p.R201C	SNV
GNAS	c.680A>T	p.Q227L	SNV
SMARCB1	c.118C>T	p.R40*	SNV
SMARCB1	c.141C>A	p.Y47*	SNV
SMARCB1	c.157C>T	p.R53*	SNV
SMARCB1	c.472C>T	p.R158*	SNV
SMARCB1	c.566_567ins19	p.L191fs*26	INS
SMARCB1	c.601C>T	p.R201*	SNV
SMARCB1	c.607G>A	p.A203T	SNV
SMARCB1	c.601+66G>C	p.(=)	SNV
SMARCB1	c.1148delC	p.P383fs	DEL
